# Gene expression profiling of epidermal cell types in *C. elegans* using Targeted DamID

**DOI:** 10.1242/dev.199452

**Published:** 2021-09-03

**Authors:** Dimitris Katsanos, Mar Ferrando-Marco, Iqrah Razzaq, Gabriel Aughey, Tony D. Southall, Michalis Barkoulas

**Affiliations:** Department of Life Sciences, Imperial College London, London SW7 2AZ, UK

**Keywords:** DamID, Seam cells, Epidermis, RPB-6, EFL-3/E2F7, HDA-1/HDAC1/2

## Abstract

The epidermis of *Caenorhabditis elegans* is an essential tissue for survival because it contributes to the formation of the cuticle barrier as well as facilitating developmental progression and animal growth. Most of the epidermis consists of the hyp7 hypodermal syncytium, the nuclei of which are largely generated by the seam cells, which exhibit stem cell-like behaviour during development. How seam cell progenitors differ transcriptionally from the differentiated hypodermis is poorly understood. Here, we introduce Targeted DamID (TaDa) in *C. elegans* as a method for identifying genes expressed within a tissue of interest without cell isolation. We show that TaDa signal enrichment profiles can be used to identify genes transcribed in the epidermis and use this method to resolve differences in gene expression between the seam cells and the hypodermis. Finally, we predict and functionally validate new transcription and chromatin factors acting in seam cell development. These findings provide insights into cell type-specific gene expression profiles likely associated with epidermal cell fate patterning.

## Teaser

**Summary:** Targeted DamID is established in *C. elegans* for tissue-specific gene expression profiling without cell isolation and is used to identify genes playing a role in seam cell development.

## INTRODUCTION

Multicellular organisms consist of a plethora of differentiated cell types and tissues that perform specialised functions to support organismal physiology. All specialised cells are determined through the establishment of distinct patterns of gene expression (Ralston and Shaw, 2008). Thus, comparisons of gene expression profiles can uncover the mechanisms at play in establishing and maintaining cell identity. Understanding transcriptome changes with high spatiotemporal resolution is also central to stem cell biology. Transcriptional differences between multipotent progenitors and differentiated cells can reveal the molecular basis of the acquisition, maintenance and plasticity of cell fate determination (Han et al., 2018)

**Fig. 1.**
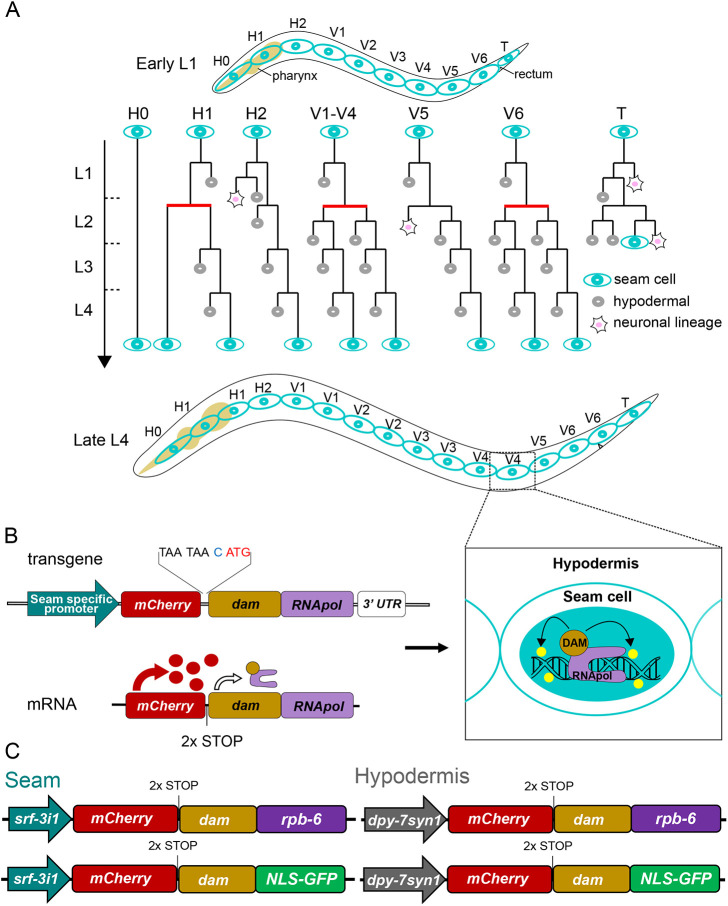
**A targeted DamID approach for cell type-specific gene expression profiling in the *C. elegans* epidermis.** (A) Postembryonic seam cell divisions for each of the initial ten seam cells of the hatched L1 animal. Symmetric divisions are denoted by horizontal red lines and asymmetric divisions by horizontal black lines. (B) The design for seam cell-specific gene expression profiling by TaDa includes a seam cell-specific promoter followed by a primary ORF of *mCherry*, two STOP codons, a frameshift (nucleotide in blue) and the coding sequence of the Dam-RNApol fusion. Expression from this transgene produces a bicistronic mRNA, which leads to very low levels of Dam-RNApol fusion protein by rare ribosomal re-initiation of translation at the secondary ORF. The result is seam cell-specific GATC methylation (yellow marks) within transcribed genes. (C) Key features of single-copy transgenes used in this study for RNApol occupancy probing by TaDa in seam cells (*srf-3i1::pes-10* promoter; left) and hypodermis (*dpy7syn1* promoter; right).

The *Caenorhabditis elegans* epidermis secretes the main constituents of the outer protective cuticle and facilitates developmental progression between larval stages and animal growth (Chisholm and Xu, 2012). A key cell type in the epidermis is the lateral seam cells, which display a stem cell-like behaviour during post-embryonic development (Joshi et al., 2010). In the newly hatched L1 larvae, there are ten seam cells on each lateral side. Three of those cells occupy the head region (H0-H2), six are in the midbody up to the rectum (V1-V6) and one is located near the tail (T) (Sulston and Horvitz, 1977). Throughout post-embryonic development, the seam cells perform a series of stem cell-like divisions. They divide both symmetrically at the L2 stage to expand their number, and asymmetrically at all larval stages, whereby one daughter cell acquires the hypodermal cell fate and fuses to the hyp7 syncytium, whereas the other maintains the seam cell fate (Fig. 1A) (Joshi et al., 2010; Sulston and Horvitz, 1977). These divisions generate 98 out of the 139 nuclei of the hyp7 syncytium (Altun and Hall, 2009), and a robust terminal seam cell number of 16 cells per lateral side is maintained, with minimal variation in the wild-type population (Boukhibar and Barkoulas, 2016; Katsanos et al., 2017).

Uncovering genes that may relate to stemness and differentiation within the epidermis requires first acquiring cell type-specific gene expression profiles. A commonly used approach to acquire cell type-specific gene expression profiles relies on cell isolation followed by RNA sequencing (RNA-seq) (Celniker et al., 2009). Although selection of cells from crude suspensions by methods such as fluorescence activated cell-sorting (FACS) has been achieved in *C. elegans* (Kaletsky et al., 2018; Spencer et al., 2011, 2014), cell isolation remains particularly challenging for some tissues, including the epidermis. This is because the epidermis is tightly attached to the cuticle and anchored to a basal lamina; therefore, cell sorting is difficult and has been achieved only in adult *C. elegans* (Kaletsky et al., 2018; Zhang and Kuhn, 2013). Other approaches, such as INTACT, rely on tissue-specific isolation of nuclei prior to mRNA extraction (Deal and Henikoff, 2011; Steiner et al., 2012); however, in this case, cytoplasmic mRNAs are not included in the identified transcriptome. Tissue-specific extraction of mRNA using transgenic expression of a poly(A)-binding protein (PABPC) and sequencing (PAT-seq) has been successfully used in *C. elegans*, but potential drawbacks relate to biases in the recovery of mRNAs based on their poly(A)-tail length and toxicity related to high levels of PABPC expression (Blazie et al., 2015, 2017; Yang et al., 2005). More recently, approaches based on single cell combinatorial indexing RNA-seq (sci-RNA-seq) have been developed (Cao et al., 2017). Despite these methods being information rich, they remain bioinformatically challenging and costly.

Targeted DamID (TaDa), first developed in flies, avoids cell isolation by revealing transcribed genes as genomic regions that associate with a fusion between Dam methyltransferase from *Escherichia coli* and an RNA polymerase subunit (Southall et al., 2013). This association leaves methylation marks on the DNA and, thus, permits the identification of genes in cells or tissues of interest for which specific promoters are available to drive the expression of Dam-RNA pol fusions. The levels of expression of the Dam fusions are crucial for the success of this approach (van Steensel and Henikoff, 2000). Constitutive or high levels of Dam expression saturate DNA with nonspecific methylation, reducing the power to identify genuine hits and causing toxicity (Southall et al., 2013; van Steensel and Henikoff, 2000). This has been addressed by making use of leaky expression of the Dam fusion from uninduced conditional promoters. However, in this case, gene identification is not tissue specific unless a recombinase-based system, such as FLP/FRT or CRE/lox, is implemented to permit expression from the uninduced promoter within a certain tissue (Gómez-Saldivar et al., 2020; Harr et al., 2020; Pindyurin et al., 2016). In TaDa, low levels of expression are achieved by using a tissue-specific promoter to drive expression of a bicistronic mRNA consisting of two consecutive open reading frames (ORFs) interrupted by two stop codons and a frameshift (Southall et al., 2013). The Dam fusion occupies the secondary ORF, which is translated very infrequently because of ribosomal re-initiation, resulting in low protein levels (Kozak, 2001; Southall et al., 2013). The frequency of the re-initiation is dependent on the size of the primary ORF (Kozak, 1987, 2001), with the length of *mCherry* found to be suitable for this application (Fig. 1B) (Southall et al., 2013). We have shown that the TaDa configuration for studies in the *C. elegans* epidermis successfully prevents toxicity and saturated methylation (Katsanos and Barkoulas, 2020 preprint). Here, we use TaDa to compare gene expression profiles between epidermal cell types in *C. elegans*. We compare the gene sets we generated with other published datasets to produce a comprehensive resource of epidermal transcriptomes for seam cells and hypodermis. Furthermore, we validate our TaDa datasets by identifying new transcription and chromatin factors as well as miRNAs involved in epidermal development.

## RESULTS

### Generation of epidermal cell type-specific Dam fusions for TaDa

To resolve cell type-specific gene expression profiles in the epidermis, we first sought to identify suitable promoters to drive TaDa transgene expression in the seam cells and hypodermis. With regard to the seam cells, we focussed on the nucleotide sugar transporter gene *srf-3*, which has been shown to be expressed in these cells by single-molecule fluorescence *in situ* hybridisation (smFISH) (Fig. S1A) and a reporter construct (Höflich et al., 2004). The *srf-3* promoter fragment used in this published construct included all the upstream region of the *srf-3* isoform b (*srf-3b*), from the 3′-untranslated region (UTR) of the upstream gene (*txt-19*) to the ATG of *srf-3b*, including the first exon and intron of *srf-3a* (Fig. S1B). We found that a seam cell-specific enhancer element is located within the first intron of *srf-3a* (named here *srf-3i1*). This element, fused to a minimal *pes-10* promoter, was sufficient to drive strong and specific GFP expression in the seam without any visible expression in other tissues, such as intestinal cells or the germline, in which expression was observed under the putative promoters of *srf-3a* and *srf-3b* isoforms (Fig. S1C). Concerning the hypodermis, the promoter of the collagen gene *dpy-7* is commonly used to drive expression in this tissue (Blazie et al., 2017; Brabin et al., 2011; Johnstone et al., 1992). However, careful microscopic observation revealed that *dpy-7p* also drives low expression levels in dividing seam cells (Fig. S1D). Thus, we constructed a synthetic version of this promoter (named *dpy-7syn1*) in which we replaced nucleotides flanking two existing GATA-binding sites from AGATA, a motif previously seen in promoters of seam cell-expressed genes (Katsanos et al., 2017), to TGATA sequences that have been associated with hypodermal expression as putative binding sites for the transcription factor ELT-3 (Gilleard and Mcghee, 2001; Shao et al., 2013). The expression driven by *dpy-7syn1* was confined to the hypodermis (Fig. S1E). Taking these results together, we conclude that *srf-3i1* and *dpy-7syn1* were the most suitable regulatory elements to drive the expression of transgenes for gene expression profiling in the epidermis.

Investigation of cell type-specific gene expression in TaDa relies on studying genome-wide RNA polymerase II occupancy. To acquire such occupancy profiles, Dam was fused to the largest RNA polymerase II subunit, *ama-1/POLR2A*, which has been successfully used in TaDa experiments in *Drosophila* (Southall et al., 2013), as well as *rpb-6/POLR2F*, which participates in all RNA polymerase complexes and has been successfully used in other DamID-based approaches (Filion et al., 2010). Transgenic lines were created to allow expression of these fusions in the epidermis at low levels under the TaDa transgene configuration (Fig. 1C). Similar constructs were made with GFP (*dam:NLS-GFP*) as control (Fig. 1C), which are able to capture background levels of methylation from accessible regions of the genome, and, thus, allow assessment of the RNApol occupancy profiles against background signal (Aughey and Southall, 2016). The expression of *mCherry* in the transgene cassette was used as proxy to confirm that all single-copy transgenes drove expression in the expected cell type (Fig. S2).

The methylation capacity of the fusions was tested by extraction and amplification of methylated genomic DNA. During these experiments, we found that the *dam:ama-1* fusion failed to produce sufficient methylation (average of 1.9 million unique mappable reads per sample), whereas *dam:rpb-6* fusions produced methylation in both cell types, with an average of 17.5 million mappable reads per sample. Therefore, we decided to pursue gene expression analysis using the *rpb-6* fusions.

### RPB-6 occupancy occurs within coding sequences and reveals spatiotemporal gene expression patterns

**Fig. 2.**
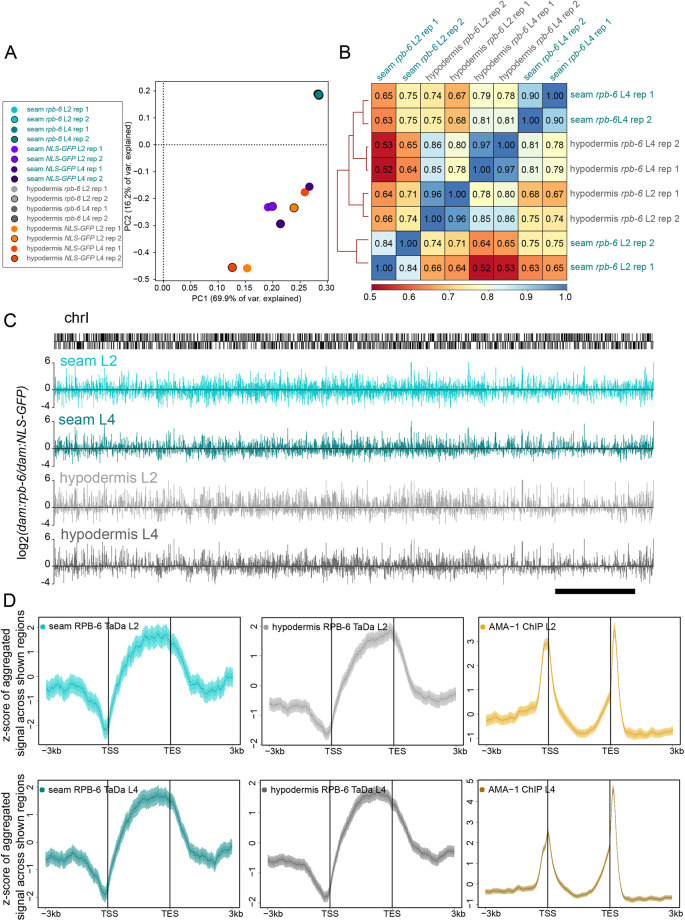
**Genome-wide RPB-6 occupancy profiles show high correlation and signal preference for gene bodies.** (A) Principal component analysis of normalised sequence-aligned read-count maps for all samples shows tight clustering of *dam:rpb-6* samples, which form a separate group from control fusion samples for both seam cells and hypodermis. (B) Summary heatmap of Pearson correlations between all *dam:rpb-6* samples based on averaged read-count normalised scores per protein-coding gene. (C) Examples of averaged signal enrichment profiles for *dam:rpb-6* occupancy across chromosome I (chrI) at the L2 and L4 stages. Locations of protein-coding genes are indicated by black vertical bars on chromosome I. (D) Aggregation plots showing either average TaDa RPB-6 signal for seam and hypodermis or the whole-animal ChIP-seq AMA-1 signal. Regions up to 3 kb upstream of the TSS to 3 kb downstream of the TES in 10 bp bins are shown. All protein-coding genes are pushed into a pseudo-length of 3 kb for the L2 and L4 samples. TaDa RPB-6 samples show increased average signal within gene sequences with preference for 3′ regions and depletion near the TSS, whereas the AMA-1 ChIP-seq signal shows peaks of increased average enrichment both over the TSS and towards the 3′ end of genes. Scale bar: 2 Mb.

To analyse gene expression profiles per cell type and stage, we sequenced samples from two larval stages (L2 and L4) from as few as ∼2000 individuals, and generated sequence alignment read count-normalised maps. To assess the overall binding profile, we compared the read-count scores of GATC fragments across the whole genome, which included intergenic areas with potentially lower signal levels if the RPB-6 fusions had retained their expected biological function. These comparisons showed separate clustering of *dam:rpb-6* and control samples based on Pearson correlation heat maps and principal component analysis (Fig. 2A; Fig. S3A,B). Correlation coefficient values were lower between controls compared with *dam:rpb-6* samples, likely because of the less-targeted nature of methylation in the absence of RPB-6. To evaluate the reproducibility between replicates at the level of genes that RPB-6 is expected to occupy, we also examined correlations restricted to GATC fragments that reside within protein-coding genes as well as read-count signal averaged across protein-coding genes. We found moderate-to-strong correlations between replicates based on average score per protein-coding gene (r=0.84-0.97; Fig. 2B) or per individual GATC fragment within genes (r=0.67-0.93; Fig. S3C), highlighting the reproducibility of genic signal acquisition as well as its reproducible distribution within protein-coding genes.

The RPB-6-occupancy TaDa signal can be calculated from stage- and promoter-matched *dam:rpb-6* and *dam:NLS-GFP* samples as normalised log_2_(*dam:rpb-6/dam:NLS-GFP*) scores per GATC fragment of the genome (see example across chromosome I in Fig. 2C). For all profiles and stages, we found consistent RPB-6 occupancy preference within genes rather than intergenic sequences at the genome-wide level (Fig. 2D). Interestingly, RPB-6 occupancy showed a preference towards the 3′ region of genes and a depletion at the 5′ region of genes, near the transcription start site (TSS). This differs from the average AMA-1 occupancy across genes seen by ChIP-seq, which shows increased occupancy near the TSS region (Fig. 2D). The difference is unlikely to reflect method-dependent biases, because AMA-1 TaDa in *Drosophila* also showed high average occupancy of TSS and transcription end sites (TES) (Southall et al., 2013). The *C. elegans* gene coordinates used to assess average occupancy here are based on the longest transcript produced by each gene; however, up to 94% of those genes also have other isoforms (Tourasse et al., 2017). Furthermore, the average positional occupancy by isoforms of annotated gene sequences shows a preference for a 3′ location (Fig. S4A), which could contribute to the difference in localisation between RPB-6 and AMA-1 occupancy. Therefore, we tested the occupancy signal across isoforms instead of genes. All TaDa and ChIP-seq profiles exhibited approximately the same patterns of average signal enrichment (Fig. S4B), suggesting that the difference in occupancy profiles may stem from the use of RPB-6 instead of AMA-1.

**Fig. 3.**
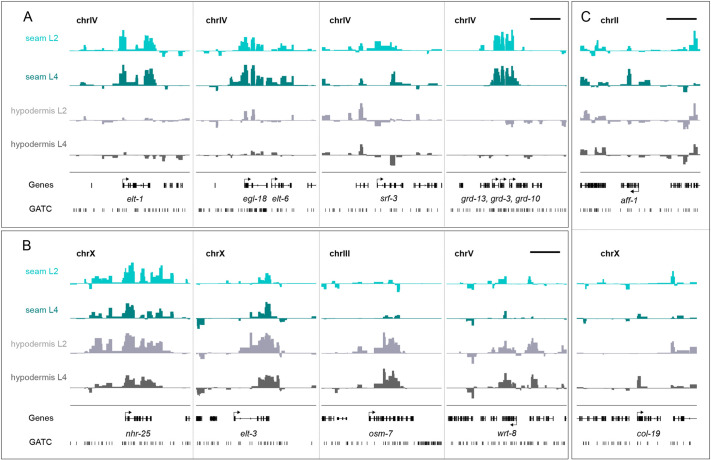
**Signal enrichment across genes with known cell-type specificity.** (A-C) Examples of the signal enrichment profiles over genes showing statistically significant RPB-6 occupancy (FDR<0.05). (A) *elt-1*, *egl-18/elt-6*, *srf-3*, and *grd13/grd-3/grd-10* show expression in seam cells. (B) *elt-3*, *osm-7* and *wrt-8* show expression mostly in hypodermis and *nhr-25* in both cell types. (C) Expression of *aff-1* and *col-19* is higher at L4, consistent with the temporal regulation of these genes. *y*-axes represent normalised log_2_*(dam:rpb-6*/*dam:NLS-GFP)* scores (data range: −2–4). Genes and GATC sites are shown in black. Scale bars: 5 kb.

To assess the potential biological relevance of the acquired occupancy signals, we focused on selected genes known to be expressed in the seam cells or hypodermis. For example, seam cell fate-promoting factors, such as *elt-1*, *egl-18* and *elt-6* (Gilleard and Mcghee, 2001; Gorrepati et al., 2013; Koh and Rothman, 2001), were found to be significantly expressed by TaDa in seam cells (Fig. 3A). Furthermore, *srf-3* and the groundhog genes *grd-13*, *grd-3* and *grd-10*, all known to be expressed in seam cells, but not hypodermis (Aspöck et al., 1999; Cao et al., 2017; Höflich et al., 2004), showed higher signal enrichment and significant occupancy in seam cells compared with hypodermis (Fig. 3A). By contrast, genes that are known to be enriched in the hypodermis, such as the transcription factor *elt-3*, the osmotic stress factor *osm-7* (Wheeler and Thomas, 2006) and the Warthog family member *wrt-8* (Aspöck et al., 1999), were enriched in hypodermal profiles (Fig. 3B). Other factors, such as *nhr-25*, which is known to be expressed in both tissues (Gissendanner and Sluder, 2000), showed signal enrichment and significant occupancy in all profiles (Fig. 3B). Finally, the seam cell-expressed terminal differentiation fusogen *aff-1*, which mediates the fusion of seam cells into a single syncytium at the late L4 stage (Sapir et al., 2007), was significantly occupied by RPB-6 at the L4 stage, but not at L2 (Fig. 3C). Similarly, the collagen gene *col-19*, which is primarily expressed in the early adult hypodermis (Liu et al., 1995), was found to show strong signal enrichment at the L4 stage (Fig. 3C). These results indicate that TaDa can be used to resolve spatiotemporal patterns of gene expression in *C. elegans*.

### TaDa-identified genes match epidermally expressed genes and relate to epidermal tissue functions

**Fig. 4.**
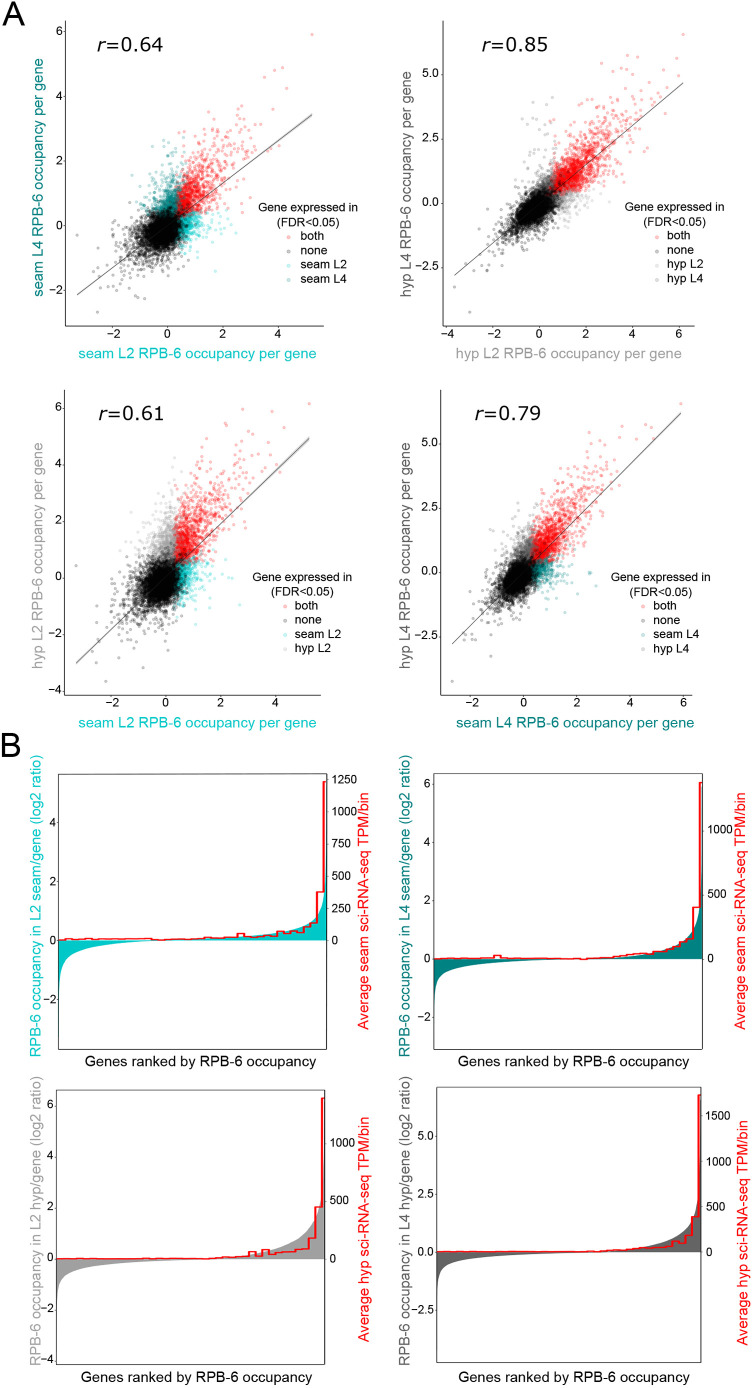
**Quantitative assessment of discovered transcriptomes.** (A) Correlation scatterplots between cell types and developmental stages of TaDa RPB-6-occupancy values per gene across all protein-coding genes. Dots correspond to individual genes and are coloured based on the sample in which they were expressed (FDR<0.05) as indicated. The Pearson's correlation coefficient *r* for each comparison is also shown. (B) Comparison of TaDa RPB-6-occupancy values for all cell types and developmental stages with cell type-matched sci-RNA-seq expression values (Cao et al., 2017). Protein-coding genes are ranked on the *x*-axis from left to right based on their RPB-6-occupancy value shown on the left *y*-axis. This ranking is compared with the averaged sci-RNA-seq expression value in bins of 500 genes shown on the right *y*-axis. The ranking is similar between TaDa and superimposed sci-RNA-seq expression levels.

To convert genome-wide TaDa signal into gene expression information, we performed gene calling to calculate an average RPB-6 occupancy value for every protein-coding gene. Given that higher frequency of transcription may produce more methylation on average, we reasoned that average occupancy values could reveal the levels of gene expression. A moderate-to-strong positive correlation was observed when comparing TaDa expression values across stages and cell types (Fig. 4A), which indicates some similarity across the TaDa-identified transcriptomes at a quantitative level. Quantitative datasets have been produced for seam cells and hypodermis by combinatorial barcoding and single-cell transcriptome clustering at the L2 stage (Cao et al., 2017). Interestingly, gene ranking by TaDa based on RPB-6 occupancy for all cell types and stages broadly aligned with the ranking of these genes based on cell type-matched sci-RNA-seq expression values (Fig. 4B).

**Fig. 5.**
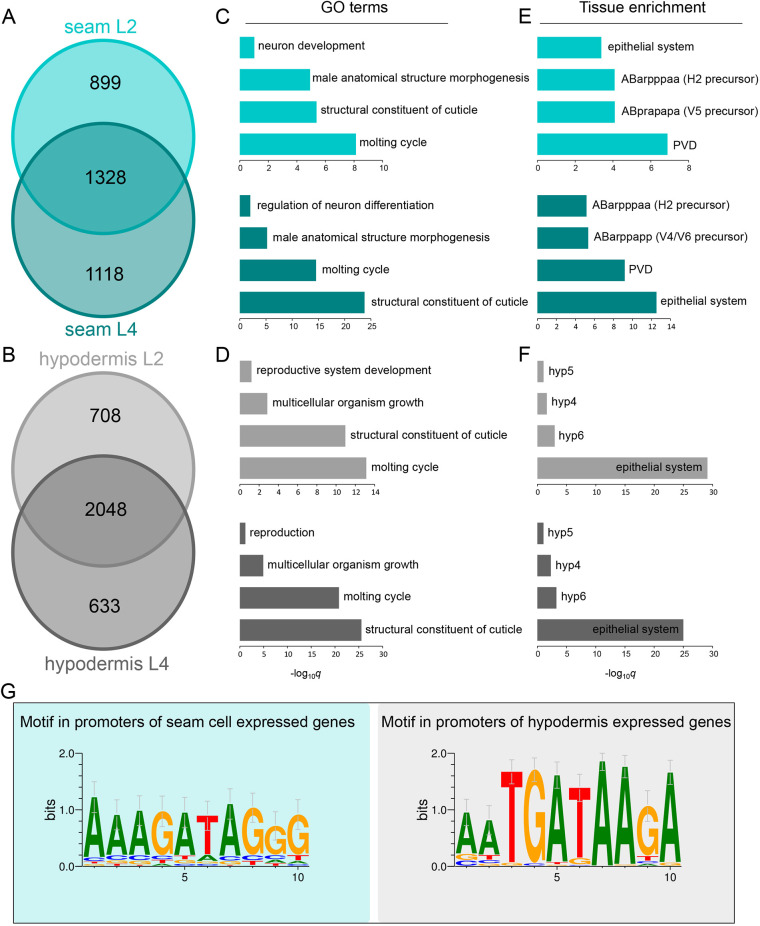
**TaDa-identified sets of expressed genes for seam cells and hypodermis are enriched for relevant GO terms and tissues.** (A,B) Venn diagram of sets of genes found to be expressed in seam (A) or hypodermis (B) by TaDa based on significant RPB-6 occupancy at L2 and L4 stages. (C,D) Plots of selected significantly enriched GO terms for seam (C) and hypodermis (D) for the L2 (top) and L4 (bottom) gene sets. (E,F) Plots of selected over-represented tissues with expression patterns significantly enriched for similarity to seam (E) or hypodermis (F) gene sets at the L2 (top) and L4 (bottom) stage. (G) *De novo*-identified motifs enriched in the sequences up to 2 kb upstream of the TSS of genes in the intersection between genes expressed at L2 and L4 for seam (left) and hypodermis (right). These motifs were found enriched only in the cell type for which they are presented.

Expressed genes per cell type and developmental stage were identified based on statistically significant RPB-6 occupancy across their sequence. This analysis revealed 2227 genes at the L2 and 2446 genes at the L4 stage for seam cells, and 2756 genes at the L2 stage and 2681 genes at the L4 stage for hypodermis (Fig. 5A,B; Table S1). In both cases, there was significant overlap in the gene sets between stages (Fig. 5A,B). To test whether TaDa identification of expressed genes is subject to bias, we investigated how expressed and nonexpressed genes may differ in their GATC numbers and gene length. We found that expressed genes were longer and had more GATCs, which is not surprising given the reliance of this method on available GATC sequences (Fig. S5A). However, the same tendency was seen when gene expression was determined based on sci-RNA-seq (Fig. S5B). This analysis also revealed that TaDa-identified genes showed higher expression based on RNA-seq (Fig. S5A) and that genes shown to be highly expressed by sci-RNA-seq showed higher TaDa expression values (Fig. S5B).

To assess the potential biological relevance of these gene sets, we pursued first Gene Ontology (GO) and tissue enrichment analysis. Both cell types were enriched for terms pertaining to the synthesis of the cuticle and moulting (Page, 2007) (Fig. 5C,D). The seam cell gene sets showed significant enrichment for neuronal GO terms, which are related to this cell type because some seam cell daughters generate neural precursors (Chisholm and Hsiao, 2012; Sulston and Horvitz, 1977) (Fig. 5C). The seam cells also give rise to sensory rays of the male tail (Sulston et al., 1980), which may be reflected by the significantly enriched term ‘male anatomical structure morphogenesis’. Likewise, the gene sets from the hypodermal *dpy-7syn1* expression domain showed enrichment for the term ‘multicellular organism growth’, which is consistent with the fact that the syncytial hypodermis is a major driver of growth in *C. elegans* (Chisholm and Hsiao, 2012) (Fig. 5D). Similarly, when assessing enrichment for tissues with known expression profiles by a tissue enrichment analysis, the ‘epithelial system’ was found to be significantly enriched for all gene sets (Chisholm and Hsiao, 2012) (Fig. 5E,F). For tissue over-representation terms, the ‘PVD’ neuron, which arises from the V5 seam cell, was found to be significantly enriched in the seam cell gene sets, along with the seam cell precursor cells ‘ABarpppaa’, ‘ABprapapa’ and ‘ABarppapp’, and ‘hyp4’, ‘hyp5’ and ‘hyp6’ in the case of hypodermis (Fig. 5E,F).

To search for putative motifs driving the expression of enriched genes, we used a region 2 kb upstream of the TSS of genes found in the intersections between the L2 and L4 profiles. Interestingly, a TGATAA motif was found to be significantly enriched in the hypodermal gene sets, which shows similarity to the binding motif for the hypodermal transcription factor ELT-3 (*P*=5.81×10^−6^) (Fig. 5G). For the seam cell datasets, we found an AGATAG motif (Fig. 5G), similar to that of the ELT-1-related human GATA2 factor (*P*=7.44×10^−4^). Notably, an AGATAG motif is found in the intron 1 of *srf-3*a as well as in the promoter region of *lin-22* and, when deleted, seam cell expression of *lin-22* is decreased (Katsanos et al., 2017). Taken together, the GO term enrichment and motif enrichment analyses suggest that the expression profiles acquired by TaDa represent genes transcribed in the epidermis.

### TaDa-determined gene lists show extensive overlap with other published datasets

We first compared the TaDa-acquired gene lists for seam cells and hypodermis to datasets derived from tissue-specific nuclear RNA experiments (Serizay et al., 2020). We found that our TaDa datasets contained ubiquitously expressed and epidermal genes, as expected (Fig. S6A). Therefore, we compared our data against transcriptomes for epidermal cell types derived via other methods, such as sci-RNA-seq and PAT-seq. In PAT-seq, epidermal tissue specificity was achieved by mRNA tagging via expression of a poly(A)-binding protein under a seam cell (*grd-10* in this case) or hypodermal (*dpy-7*) promoter (Blazie et al., 2017). All gene-set intersections for seam cells across methods were highly significant (*P*≤2.2×10^−162^ with a Fisher's exact test), with 72.4% of the TaDa-defined seam cell genes at L2 and 79% of the L4 genes being present in the sci-RNA-seq dataset (Fig. S6B). The overlaps with PAT-seq were smaller (33.8% for L2 and 33.9% for L4), but also significant (Fig. S6B). Similar observations were made upon comparing the hypodermal datasets, with 76.1% and 79.2% of TaDa genes overlapping with sci-RNA-seq and 67.4% and 71.3% overlapping with PAT-seq for the L2 and L4 gene-sets, respectively (Fig. S6C). Based on this considerable agreement, we present here detailed datasets for genes expressed in seam cells and hypodermis, as supported by TaDa alone or in combination with sci-RNA-seq and PAT-seq (Table S1).

Sci-RNA-seq and PAT-seq are thought to be quantitative methods that can reveal levels of expression for each transcript. Therefore, we examined correlations between sci-RNA-seq or PAT-seq and TaDa expression values. The assessment was performed for both seam cells and hypodermis using the L2 datasets, which were stage matched to the sci-RNA-seq datasets. Interestingly, all comparisons showed a statistically significant correlation with a weak positive association (Pearson's correlation test: *P*<0.05 for all; Fig. S6D). Therefore, it is interesting that expression levels from these methods tend to align, despite the overall differences in what these techniques measure.

### TaDa identifies new transcription factors and chromatin regulators acting in seam cell development

Next, we compared the TaDa-identified genes across cell types by performing multiple intersections. Interestingly, the largest subset was the overlap between all gene sets (1035 genes) with 37.5-46.4% of genes from each set present in the overlap (Fig. S7A). All pairwise and higher-order overlaps of gene sets across cell types and developmental stages were significant (Fig. S7B). Based on all intersections between TaDa expression profiles, we identified a set of genes putatively expressed in the seam cells, but not the hypodermis, and vice versa. Notably, these TaDa gene sets were enriched for genes identified as being specifically expressed in the same cell type using sci-RNA-seq (Fig. S7C,D).

**Fig. 6.**
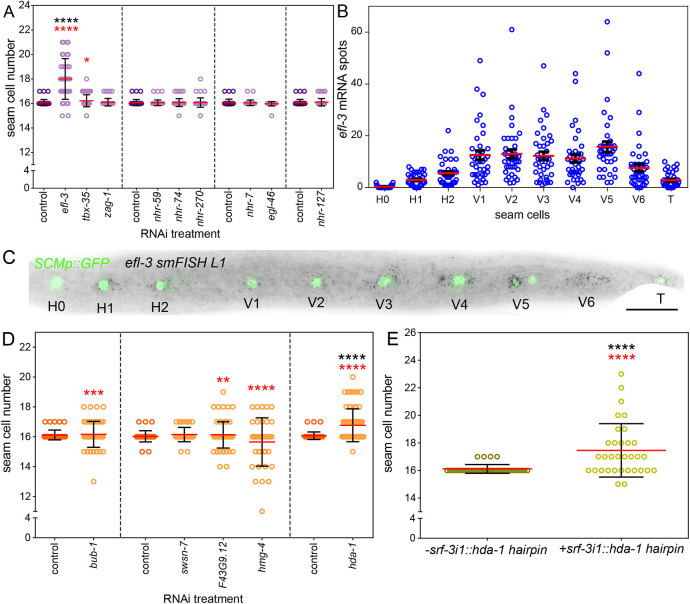
**TaDa identifies new regulators of seam cell patterning.** (A) Quantification of seam cell number in the late L4 stage of RNAi-treated animals carrying the seam cell marker *SCMp::GFP* (34≤*n*≤59 animals per treatment). (B) Quantification of *efl-3* smFISH mRNA spots per seam cell of late L1 animals (*n*=38). (C) Representative smFISH from three independent experiments in wild type at the late L1 stage, showing *efl-3* expression (black spots) in seam cells marked by *SCMp::GFP*. (D) Quantification of seam cell number in post-embryonic RNAi treatments for the chromatin factors indicated on the *x*-axis in late L4 stage animals carrying the *SCMp::GFP* reporter (36≤*n*≤73). (E) Expression of an *hda-1* hairpin in the seam cells (*n*=33) increases seam cell number compared with controls, which do not carry the hairpin (*n*=36). In A and D, sets of treatments performed on the same day are grouped by dashed lines. In A, B, D and E, the red line indicates the mean. Error bars represent s.d. in A, D and E, and s.e.m. in B. Seam cell scorings were repeated at least twice. Black asterisks indicate statistically significant differences to the mean with either a one-way ANOVA and a Dunnett's post-hoc (A,D) or a two-tailed *t*-test (E). Red asterisks indicate statistically significant differences in variance with a Levene's median test. In all cases, *P<0.05, **P<0.01, ***P<0.001, ****P<0.0001. Scale bar: 20 μm.

We argued that the seam cell list may contain factors that play a role in seam cell development; thus, we decided to compare this list against a list of known transcription and chromatin factors in *C. elegans* and to test the function of selected candidates (Table S2). We found 58 transcription factors in the overlap, including well-known transcription factors expressed in seam cells, including *elt-1*, *ceh-16*, *egl-18*, *elt-6* and *nhr-73* (Cassata et al., 2005; Koh and Rothman, 2001; Miyabayashi et al., 1999; Gorrepati et al., 2013; Huang et al., 2009; Smith et al., 2005). We selected a subset for which there was no prior evidence for a function in seam cell development to perform a small-scale RNAi screen using terminal seam cell number as the phenotypic readout, which reflects the fidelity of developmental patterning. Interestingly, two out of nine RNAi treatments were found to have a significant effect on seam cell number (Fig. 6A). The most striking was the knockdown of the E2F transcription factor *efl-3*, which caused a significant increase in average seam cell number from 16.07 in the control to 18 in the RNAi-treated animals (*P*<0.0001 with a one-way ANOVA) (Fig. 6A). To confirm whether *efl-3* is expressed in seam cells, we studied its expression pattern by smFISH. We found evidence for expression in seam cells, with stronger expression in V lineage cells (Fig. 6B,C). This observation validates *efl-3* as a seam cell-expressed regulator influencing epidermal development.

Within the set of 1090 seam cell genes, we found 35 putative chromatin factors (Table S2), a subset of which was also targeted by RNAi. Among the targeted factors, the kinetochore-binding factor *bub-1* was included as a control because of its known functions in multiple embryonic and post-embryonic lineages, including seam cells (Wang et al., 2009). Other factors included high mobility group factors (*hmg-11*, *hmg-1.1* and *hmg-4*), chromodomain helicases (*chd-1* and *chd-3*) and a histone deacetylase (*hda-1*), as well as the SWI/SNF chromatin remodelling complex factor *swsn-7*. The screen was initially attempted with onset of RNAi treatment at the L4 stage of the previous generation to the one that was scored, allowing for likely depletion of maternal deposition of the targeted genes. We did not find any significant change in the mean seam cell number or variance in the population (Fig. S8). However, for some treatments (*bub-1*, *hda-1*, *hmg-4*, *swsn-7* and *F43G9.12*), we observed embryonal lethality, which prompted us to perform post-embryonic RNAi for these factors. In this case, *bub-1*, *F43G9.12* and *hmg-4* RNAi showed a significant increase in seam cell number variance in the population (Fig. 6D). More strikingly, *hda-1* RNAi led to a significant increase in seam cell number (Fig. 6D). This is likely the result of a cell-autonomous role for *hda-1* in the seam, because seam cell number increase was reproduced with a seam cell-driven *hda-1* hairpin construct (Fig. 6E). Taken together, our results show that TaDa can be used to predict new factors influencing epidermal development.

### TaDa reveals miRNAs with potential epidermal functions

**Fig. 7.**
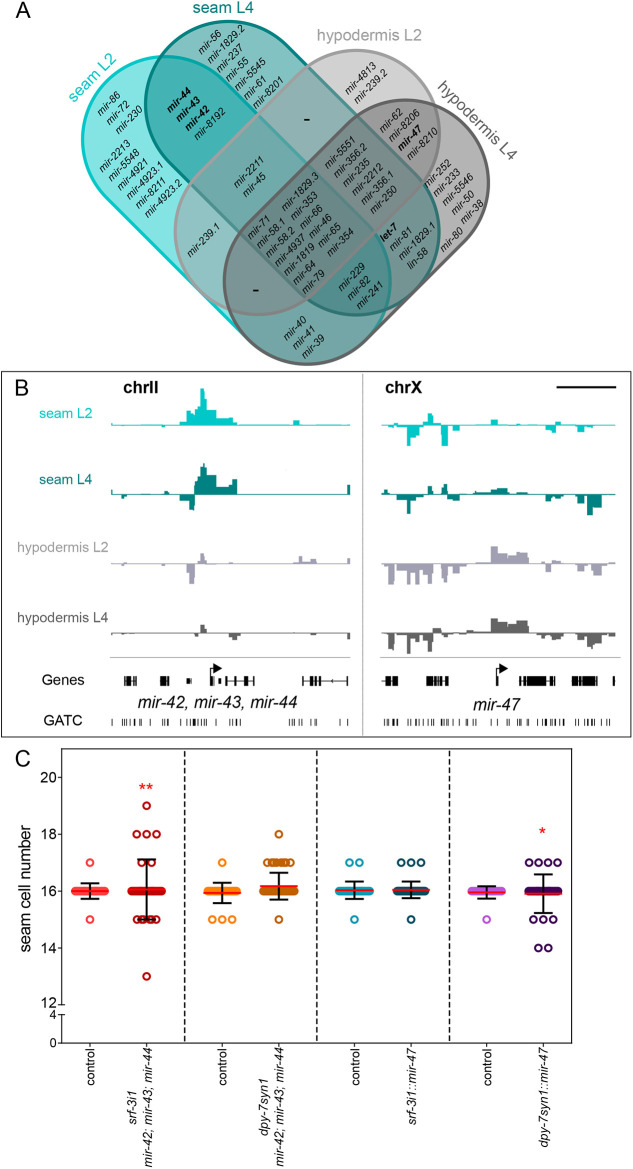
**TaDa identifies miRNAs expressed in the epidermis with putative developmental functions.** (A) Venn diagram listing all miRNAs found to be expressed by TaDa in each cell type and stage. (B) Signal enrichment in both cell types at the L4 stage around the locus of *mir-42*, *mir-43*, *mir-44* only in seam profiles and around *mir-47* only in hypodermis profiles. The *y*-axes represent log_2_*(dam:rpb-6*/*dam:NLS-GFP)* scores (data range: −2–4). (C) Quantification of seam cell number by counting *SCMp:GFP* nuclei at the late-L4 stage in animals overexpressing either *mir-47* or the *mir-42*, *mir-43*, *mir-44* cluster in both cell types. There is an increase in seam cell number variance upon overexpression within the native expression domain compared with controls without the extrachromosomal array (21≤*n*≤53 for controls, 31≤*n*≤47 for transgenics). Seam cell scorings were repeated twice. Red lines indicate the mean; error bars represent s.d. Red asterisks indicate statistically significant differences in variance with a Levene's median test. *P<0.05, **P<0.01. Scale bar: 5 kb.

Given that TaDa allows the discovery of small RNAs expressed in a tissue without any modification of the experimental protocol, we investigated miRNA expression in epidermal cell types. Of the 256 annotated mRNAs of the *C. elegans* genome, 64 showed expression in seam cells and/or hypodermis by TaDa. More specifically, 35 miRNA genes were found to be significantly occupied in the seam cell datasets at L2 and 39 at L4, compared with 28 in the hypodermal dataset at L2 and 39 at L4 (Fig. 7A). Intersections of those miRNA revealed that most were shared across expression domains, likely indicating housekeeping or broad epidermal functions, whereas others were uniquely expressed in one cell type (Fig. 7A). For example, the miRNA cluster *mir-42*, *mir-43* and *mir-44* was found to be expressed only in seam cells in both stages, whereas *mir-47* was found to be expressed only in hypodermis in both stages (Fig. 7B), which is consistent with previous reports of epidermal gene expression for these miRNAs based on transcriptional reporters (Kinser et al., 2021; Martinez et al., 2008). To address whether these miRNAs may have functions in epidermal development, we overexpressed them in the epidermis using the same promoters used for TaDa. The miRNAs *mir-42*, *mir-43*, *mir-44* form an operon on their genomic locus on chromosome II (Martinez et al., 2008); thus, all three miRNAs were overexpressed as a single unit. Interestingly, both the *mir-42*, *mir-43*, *mir-44* cluster and *mir-47* significantly increased seam cell number variance when overexpressed within their endogenous domain, and not when they were overexpressed in the other epidermal cell type (Fig. 7C). These findings underscore the value of TaDa in revealing not only protein-coding genes, but also miRNAs expressed in a tissue of interest.

## DISCUSSION

### RPB-6-occupancy profiles reveal genes expressed in closely related cell types

We introduce TaDa as a method to study tissue-specific gene expression profiles in *C. elegans*. This method is based on investigating genome-wide occupancy of RPB-6, a subunit that participates in all RNA polymerase complexes, as a proxy to identify transcribed genes in the tissue of interest without cell isolation. We focussed on the *C. elegans* epidermis, for which gene expression profiles are less studied compared with other tissues. We demonstrated that we can resolve gene expression profiles in epidermal cell types and used these data to predict new regulators influencing epidermal development.

Analysis of the RPB-6-occupancy profiles revealed enrichment within genes, with the average RPB-6 signal being depleted near the TSS and increased closer to the 3′ end. This localisation is consistent with a recent report (Gómez-Saldivar et al., 2020), but does not fully match AMA-1 occupancy peaks near the TSS and the TES of genes previously observed in ChIP-seq in *C. elegans* or TaDa in *Drosophila* (Araya et al., 2014; Southall et al., 2013). The AMA-1 *Drosophila* homologue RpII215 has produced methylation patterns in a fusion with Dam in the TaDa configuration (Southall et al., 2013). However, we found that *dam:ama-1* fusions produced very little methylation in *C. elegans*, which was also observed in attempts to express this transgene in other *C. elegans* tissues (Gómez-Saldivar et al., 2020). We speculate that inherent differences in the function of these subunits may explain the localisation patterns of methylation. RPB-6 is known to stabilise the transcribing polymerase on the DNA (Ishiguro et al., 2000); thus, the structural conformation of the initiation complex may obstruct DAM:RPB-6 from methylating GATCs in the vicinity of the start site. Instead, as transcription starts and the polymerase disengages from various components of the initiation complex (Hahn, 2004), the DAM:RPB-6 fusion may gain access to methylate DNA over the rest of the gene sequence.

### Strengths and weaknesses of TaDa as a method for tissue-specific gene expression profiling in *C. elegans*

Two other methods have produced so far comparable information for gene expression profiles in seam cells and hypodermis, namely PAT-seq and sci-RNA-seq (Blazie et al., 2017; Cao et al., 2017). Comparisons between these methods and TaDa revealed good agreement, with extensive qualitative overlaps; for example, ≥72.4% of the total genes identified by TaDa were also identified with sci-RNA-seq. This is encouraging especially because TaDa measures a distinct biological quantity as a proxy of gene expression and, thus, some differences between datasets were anticipated.

All methodologies have their own strengths and weaknesses. Both sci-RNA-seq and PAT-seq rely on a larger amount of starting material (Blazie et al., 2017; Cao et al., 2017). With TaDa, even for seam cells, which constitute a small fraction of the total cells (32/∼1000), we were able to acquire an average of 17.5 million unique reads starting from a moderately sized population of around 2000 individuals. Furthermore, the TaDa bicistronic mRNA design minimises Dam-fusion expression levels and, thus, overcomes transgene-associated animal toxicity (Katsanos and Barkoulas, 2020 preprint). This is a limitation in PAT-seq because of the high expression of a poly(A)-binding protein, which can influence the acquired gene expression profiles (Yang et al., 2005). Moreover, TaDa allows profiling of the tissue-specific expression of small RNAs within the same experiment, which cannot be achieved by RNA seq-based approaches without introducing specialised protocols for RNA isolation or small RNA tagging (Alberti et al., 2018; Lu et al., 2007). By contrast, DamID-based methods have limited ability to capture dynamic changes in gene expression because it is difficult to rule out methylation carry-over. Another key advantage of sci-RNA-seq over TaDa is that a single experiment allows the elucidation of transcriptomes for all cell types of *C. elegans*, which makes sci-RNA-seq cost effective relative to the wealth of information that it can create. Nevertheless, attribution of single-cell transcriptomes to specific cell types in sci-RNA-seq relies on specific gene markers and can be challenging for certain cell types that do not cluster sufficiently apart (Cao et al., 2017).

The total number of genes identified by TaDa per cell type and stage was lower compared with sci-RNA-seq. This may indicate decreased sensitivity of TaDa to detect small genes or genes with low expression. TaDa relies on the availability of GATC sites, which may be limited within certain genes. A key example is the bHLH Hes-like *lin-22* transcription factor, which is expressed in seam cells (Katsanos et al., 2017), but was not detected by TaDa. This gene only contains two GATC sites, 2 kb apart, and methylation of both sites would be required for the occupancy to pass the significance threshold. However, lack of available GATC sites within genes is unlikely to be pervasive in *C. elegans*, in which the average length of GATC fragments within protein-coding genes is only 518.4 bp and the median 183 bp. Furthermore, we showed that similar biases in detecting expressed genes as a function of gene length are also likely to exist in datasets generated using RNA-seq methods. Genes missing from high-throughput datasets are a common feature; for example, PAT-seq seam cell datasets do not include either core seam cell genes, such as *elt-1*, *ceh-16* and *srf-3*, or all new genes identified by TaDa and validated in this study.

### Functional validation of TaDa-predicted transcription factors, chromatin factors and miRNAs reveals previously unidentified regulators of epidermal development

We validated TaDa predictions using targeted RNAi screens. Although a partial RNAi effect could account for the absence of a significant phenotype for some of the transcription or chromatin factors targeted, we were able to identify new genes involved in epidermal development. Regarding transcription factors, we identified a new role for *efl-3* in seam cell patterning. Its human homologue, *E2F7*, is an atypical E2F that is thought to act as a repressor by competing with other E2F genes for binding to targets (Di Stefano et al., 2003; Endo-Munoz et al., 2009; Li et al., 2008). In *C. elegans*, EFL-3 is known to supress cell death in the VA and VB cells of the ventral cord (Winn et al., 2011). Seam cell daughters undergo endoreduplication before adopting the hypodermal fate, and E2F7 has been linked with the regulation of polyploidisation in specialised mammalian cells (Lammens et al., 2009). Terminal seam cell number counting lacks the resolution required to understand fully the developmental basis of the defect upon *efl-3* knockdown; thus, further experiments will be necessary to dissect the exact function of EFL-3 in the epidermis. Based on the observed increase in seam cell number upon RNAi knockdown, we speculate that EFL-3 is likely to facilitate cell differentiation in the epidermis.

In the case of chromatin factors, perturbation of *hmg-4*, *F43G9.12* and *hda-1* by RNAi was shown to affect terminal seam cell number. First, *hmg-4* is a member of the histone chaperone ‘facilitates chromatin transcription’ (FACT) complex, is a homologue of the human SSRP1, and has been shown to be expressed in multiple somatic tissues (Kolundzic et al., 2018; Suggs et al., 2018). The FACT complex has been shown to act as a cell fate barrier in *C. elegans* and mammalian systems (Kolundzic et al., 2018). In *C. elegans*, it has been mostly studied in the context of regulating cell cycle timing in the embryo, whereby *hmg-4* acts redundantly with its paralogue *hmg-3*, as well as in the intestine, where it is thought to maintain inaccessible chromatin states to prevent gene expression activation related to cell fate reprograming (Kolundzic et al., 2018; Suggs et al., 2018). *F43G9.12* is a homologue of the mammalian PAX3 and PAX7 binding protein 1 (PAXBP1). In mice, it has been shown to act by binding to the paired-box transcription factors Pax3 or Pax7 and the histone 3 lysine 4 (H3K4) methyltransferase complex, which are required for the proliferation of myoblasts by regulating cell cycle genes (Diao et al., 2012). It is interesting that these two chromatin factors appeared to modulate seam cell number variance without altering the mean, which may reflect regulation of a broad array of genes with potentially opposing functions in the epidermis. Finally, *hda-1* encodes a histone deacetylase and is the homologue of human *HDAC1* and *HDAC2*. Although *hda-1* has not been studied in seam cells, it is required for the development of the male sensory rays (Choy et al., 2007), which arise from seam cell divisions. Furthermore, *hda-1* has been shown to instruct correct transcriptional programmes during embryogenesis by associating with POP-1 (Calvo et al., 2001), which has established roles in seam cell development as a key effector of the Wnt/β-catenin pathway. HDA-1 is linked to differentiation events, for example during the transition of the anchor cell towards an invasive fate (Matus et al., 2015). Work on the mammalian *hda-1* homologues has highlighted diverse roles in haematopoiesis that are context and co-factor dependent (Wang et al., 2020). Therefore, it is conceivable that HDA-1 acts in the epidermis to drive cell differentiation or maintenance of the differentiated state.

Finally, we took advantage of the use of RPB-6 in TaDa, which allows us to capture total transcription (Gómez-Saldivar et al., 2020), to identify miRNAs expressed in specific epidermal cell types. For example, we found evidence that the *mir-42*, *mir-43*, *mir-44* cluster is specifically expressed in seam cells, whereas *mir-47* is expressed in hypodermis. Previous work indicated that *mir-42* and *mir-43* are predominantly expressed during embryonic development, whereas *mir-44* expression persists in larvae (Lau et al., 2001). Therefore, the overexpression phenotype in seam cells may stem from a shift in the timing of miRNA expression or may be due to a threshold effect. In the case of *mir-47*, it is particularly interesting that expression in hypodermis, but not seam cells, causes a seam cell phenotype in a non-cell-autonomous manner. Further investigation is required to identify the targets of these miRNAs and dissect their mode of action. Our findings highlight the potential of TaDa to contribute to our understanding of the tissue-specific gene networks underlying cell fate decisions in different developmental contexts.

## MATERIALS AND METHODS

### *C. elegans* maintenance

The *C. elegans* strains used in this study were maintained according to standard protocols (Corsi, 2006) on Nematode Growth Medium (NGM) plates, grown monoxenically on a lawn of *E. coli* OP50 at 20°C. For TaDa experiments, strains were grown on a lawn of a *dam^−^/dcm^−^ E. coli* mutant of the K12 strain (New England Biolabs, C2925). The laboratory reference N2 strain was used as the reference strain. A complete list of strains used in this study is available in Table S3.

### Molecular cloning

To test the specificity of hypodermal promoters, pIR6 (*pdpy-7::unc-54 3′UTR*) was digested with EcoRI and SmiI (ThermoFisher Scientific) to remove the *pdpy*-7 promoter and linearise the plasmid. Using oligos MBA270 and MBA271, the promoter of *dpy-7* was amplified while altering two GATA sites on the 5′ and the 3′ of the sequence to form the *dpy-7syn1* promoter, which was inserted by Gibson in the digested pIR6 to form the pIR16(*dpy-7syn1::unc-54 3′UTR*) plasmid. pIR16 was linearised with SmiI digestion and the sequence of *mCherry-H2B* was amplified using oligos DK46 and DK47 from a pre-existing pENTR mCherry-H2B plasmid and inserted by Gibson in pIR16 to form pDK18 (*dpy-7syn1::mCherry-H2B::unc-54 3′UTR*).

To study the expression pattern of the putative seam cell-specific promoter of *srf-3*, three versions of the promoter were amplified from N2 lysate. The promoter of isoform a was amplified using DK33 and DK34 oligos and was used in a Gibson assembly along with pCFJ151 as backbone, *C. elegans* optimized GFP amplified from JH01 (Heppert et al., 2016 preprint) with DK35 and DK36 oligos, H2B amplified from the pENTR mCherry-H2B plasmid mentioned above using oligos DK37 and DK38, and *unc-54* 3′UTR amplified with DK39 and DK40 oligos from pIR6. The resulting construct was pDK16 (*srf-3ap::GFPo-H2B::unc-54 3′UTR+cb-unc-119*). This was then digested with NheI/XmaJI (ThermoFisher Scientific) to remove the promoter and replace it with the isoform b promoter *srf-3bp*, amplified using either oligos DK33 and DK59 or the *srf-3* intron 1, amplified with oligos DK64 and DK65 and fused by fusion PCR to the *pes-10* minimal promoter amplified with DK66 and DK67 from L3135 (Fire Lab Vector Kit) to create pDK26(*srf-3bp::GFPo-H2B::unc-54 3′UTR*) and pDK32(*srf-3i1::pes-10::GFPo-H2B::unc-54 3′UTR*). In this study, the *srf-3i1:pes-10* promoter is generally referred to as *srf-3i1* for simplicity.

To build the constructs for epidermal RNApol TaDa, the *srf-3i1::pes-10* promoter was amplified with DK89 and DK90 from pDK32 and the *dpy-7syn1* with DK113 and DK114 from pDK18, and donor vectors were produced via a BP reaction (pDK44 and pDK61, respectively). pDK7 (Katsanos and Barkoulas, 2020 preprint) digested with PaeI was used to insert *rpb-6* amplified with DK27 and DK28 from N2 lysate and *NLS-GFP* amplified form pPD93_65 with DK43 and DK44, downstream and in-frame with *dam*. The two intermediate plasmids were inserted in parallel LR reactions with pDK44 and pDK61 to produce four different plasmids: pDK54(*srf-3i1::pes-10::mCherry::Dam-myc:NLS-GFP::unc-54 3′UTR+cb-unc-119*); pDK55(*srf-3i1::pes-10::mCherry::Dam-myc:rpb-6::unc-54 3′UTR+cb-unc-119*); pDK64(*dpy-7syn1::mCherry::Dam-myc:NLS-GFP::unc-54 3′UTR+cb-unc-119*); and pDK65(*dpy-7syn1::mCherry::Dam-myc:rpb-6::unc-54 3′UTR+cb-unc-119*). To test *ama-1* for TaDa, the p304(*cb-unc-1119+phsp-16::ama-1:Dam::unc-54 3′UTR*) plasmid (kindly donated by Peter Meister, University of Bern, Switzerland) was converted into a TaDa versatile vector with a gateway docking site for easy promoter insertion by digesting with XmaJI/PteI (ThermoFisher Scientific) to remove the existing promoter along with a part of *Dam*. From pDK7 using the primers DK41 and DK42, a compatible Gibson amplicon containing *attR4-L1::mCherry::dam(part)* was amplified and used in a Gibson assembly with the above vector to produce pDK20(*cb-119+attR4-L1::mcherry::Dam-myc::ama-1::unc-54 3′UTR*). pDK20 was used in LR reactions with pDK44 and pDK61, as above, to produce pDK46(*cb-unc-119+srf-3i1::pes-10::mCherry::Dam-myc:ama-1::unc-54 3′UTR*) and pDK62(*cb-unc-119+dpy-7syn1::mCherry::Dam-myc:ama-1::unc-54 3′UTR*).

To produce a seam cell *hda-1* hairpin construct, a fragment from the *hda-1* gene overlapping the second and third exons was amplified using oligos DK247 and DK248. The amplicon was inserted with a Golden Gate reaction in pDK157(*srf-3i1-mut::Δpes-10::outron::non-palGGBpiI::srf-3a intron5::non-palGGEsp3I::p10 3′UTR*) (Hintze et al., 2021) to produce pDK158(*srf-3i1-mut::Δpes-10::outron::>hda-1 fragment>::srf-3a intron5::<hda-1 fragment<::p10 3′UTR*).

To assemble the miRNA overexpression constructs, the vectors pDK127 carrying a *dpy-7syn1* promoter and *p10 3′UTR* and pDK82 carrying a *srf-3i1::Δpes-10* promoter and *p10 3′UTR* were both digested with XmaJI and PacI to remove sequences between the promoter and 3′UTR. The miRNAs *mir-42*, *mir-43* and *mir-44* were amplified together to ensure correct processing using oligos DK217 and DK218 as well as DK219 and DK218. The two amplicons were inserted in the above digested pDK82 and pDK127, respectively, to form pDK133(*srf-3i1::Δpes-10::mir-42-44::p10 3′UTR*) and pDK147(*dpy-7syn1::mir-42-44::p10 3′UTR*), respectively. Similarly, *mir-47* was amplified using the pairs of oligos DK220 and DK221 and oligos DK222 and DK221 to produce a pDK127- and a pDK82-compatible product that were inserted by Gibson assembly to create pDK139(*srf-3i1::Δpes-10:::mir-47::p10 3′UTR*) and pDK148(*dpy-7syn1::mir-47::p10 3′UTR*), respectively. A complete list of the oligos used in this study is shown in Table S4.

### Transgenesis

Transient transgenesis by formation of multicopy extrachromosomal arrays through microinjection was achieved following established protocols (Corsi, 2006; Evans, 2006; Mello et al., 1992). Stable transgenic lines with single-copy locus-specific inserted transgenes were produced for the purposes of this study using the *Mos1-*mediated single-copy insertion (MosSCI) method (Frøkjær-Jensen et al., 2014) and standard protocols (Nance and Frøkjær-Jensen, 2019). The EG6699 strain was used to obtain all single-copy insertions in this study. To improve screening, ‘reverse chunking’ was performed (O'Connell, 2010) and all resulting lines were molecularly confirmed for single-copy insertions using oligos NM3880 and NM3884. All the transgenes generated in this study as well as the make-up of injection mixes used are presented in Table S5.

### Microscopy

For phenotyping, live animals were mounted on fresh 2% agarose pads containing 100 μM NaN_3_ for immobilisation on glass slides. The slides were then imaged using an inverted Ti-eclipse fully motorised epifluorescence microscope (Nikon) with a metal halide light source fitted with an iKon M DU-934 camera (Andor) controlled via the NIS-Elements software (Nikon). Scoring of the terminal seam cell number phenotype was performed for the lateral side most proximal to the objective in late-L4 or early-adult animals carrying the *SCMp::GFP* marker.

To perform smFISH, animals were synchronised by bleaching and were subsequently grown at 20°C for 18 h to reach the late L1 and for 25 h for the L2 asymmetric seam cell division stage, confirmed by microscopy. Animals were fixed and smFISH was performed according to established protocols (Barkoulas et al., 2013; Katsanos et al., 2017) using probes made up of pools of 38-48 oligos fluorescently labelled with Cy5 (sequences of probe oligos are listed in Table S4). Imaging was performed using the set-up outlined above with settings as previously described (Katsanos et al., 2017), and analysis and probe signal quantification was performed using a custom MATLAB (MathWorks) pipeline (Barkoulas et al., 2013).

### Targeted DamID

Strains for TaDa experiments were separated into two biological replicates and were grown on *dam^−^/dcm^−^* plates. For each replicate, nine 55 mm *dam^−^/dcm^−^* plates fully populated with gravid adults were bleached to isolate embryos, which were then seeded on new *dam^−^/dcm^−^* plates and incubated at 20°C. Half of the resulting populations were collected in M9 buffer after 24 h at the L2 stage, and the other half after 48 h at the L4 stage. This was followed by thorough washing of the resulting animal pellets with M9, as previously described (Katsanos and Barkoulas, 2020 preprint). Genomic DNA was extracted from each sample, representing a biological replicate for each Dam fusion at a given developmental stage; GATC-methylated fragments were isolated and PCR amplified according to an adapted targeted DamID protocol for *C. elegans* (Katsanos and Barkoulas, 2020 preprint). Library preparation and Next Generation Sequencing on an Illumina HiSeq 4000 platform was performed by GENEWIZ on the resulting products.

### Calculation of TaDa signal profiles for RPB-6 occupancy and gene calling

FASTQ files representing single-end reads for each sample and replicate were mapped on the *C. elegans* genome. Sequence alignment read-count maps were generated and normalised log_2_(*dam:rpb-6/dam:NLS-GFP*) ratio scores were calculated per GATC fragment of the genome using the perl script damidseq_pipeline v1.4.5 (Marshall and Brand, 2015) (available at https://github.com/owenjm/damidseq_pipeline). The pipeline was used calling Bowtie 2 v2.3.4 (Langmead and Salzberg, 2012) to map reads on *C. elegans* bowtie indices from assembly WBcel235 (available from Illumina iGenomes page), Samtools v1.9 (Li et al., 2009) for alignment manipulations, and a GATC-fragment interval file across the *C. elegans* genome in GFF format (Katsanos and Barkoulas, 2020 preprint). For every cell type at a given developmental stage, each of the *dam:rpb-6* and *dam:NLS-GFP* fusions were represented by two replicates, permitting four pairwise genome-wide log_2_(*dam:rpb-6/dam:NLS-GFP*) calculations. Those were averaged into a single signal profile of log_2_(*dam:rpb-6/dam:NLS-GFP*) enrichment scores per GATC of the genome for every cell type and developmental stage, which were used for all downstream processing. Visualisation of the signal tracks and other genomic features presented in this study was performed using the SignalMap NimbleGen software (Roche) and Integrative Genomics Viewer (IGV) (Robinson et al., 2011).

To identify transcribed genes based on the enrichment of the RPB-6 occupancy signal over gene bodies, the Rscript polii.gene.call (Marshall and Brand, 2015) (available at https://github.com/owenjm/polii.gene.call) was used. The averaged signal profiles described above were used as input along with a genomic interval file listing genes and coordinates from the release 93 annotation of the WBcel235 assembly. A false discovery rate (FDR) lower than 0.05 was used as the threshold to call expressed genes. The outputted gene lists were updated to the Ensemble release 99 annotation post-processing (complete lists of expressed genes per cell type and developmental stage are presented in Table S1). To call expressed miRNAs using the RNA pol TaDa signal profiles, the miRNA genomic coordinates used here were extended 500 bp upstream and 500 bp downstream to expand their size for better FDR assignment.

### Pearson's correlation and principal component analysis

The Pearson's correlation between samples was assessed using the deeptools3 (Ramírez et al., 2016) multiBamSummary (--binSize 300 or using a bed file of GATC coordinates or coordinates of protein-coding genes) and plotCorrelation (--corMethod pearson, --whatToPlot heatmap or scatterplot, --skipZeros, --removeOutliers) tools on Galaxy (https://usegalaxy.eu/). Correlations here were calculated using mapped read-count signal either binned on genome-wide GATCs, genic GATC fragments or across protein-coding genes. Principal component analysis was performed using the deeptools3 plotPCA tool on the multiBamSummary matrices. For both analyses, regions with very high or zero values for both compared samples were excluded to prevent artificial inflation of correlation levels.

### Aggregation plots of signal localisation

Aggregation plots were generated using the SeqPlots GUI application (Stempor and Ahringer, 2016), with settings specified individually for each presented result. Aggregation plots represent signal averages for every 10 bp bins in regions of specified length around positional features of the genome. Start and end coordinates of genes based on the largest transcript were used as the TSS and TES, were anchored to two positions of the *x*-axis and their genic sequence was pushed or stretched to a specified pseudo-length. For each bin around the feature an average was calculated across all the features to generate the aggregation plot line. When a shaded area is shown, it represents the 95% confidence interval. When *z*-scores are presented on the *y*-axes, these have been calculated as deviations from the mean signal seen across the plotted region.

### Assessment of overlaps between gene sets

Statistical significance of overlaps between sets of coding genes was calculated using either a hypergeometric distribution test on http://nemates.org/MA/progs/overlap_stats.html or a Fisher's exact test using the R software package SuperExactTest (Wang et al., 2015). For both tests, the sampling pool was set to 20,191, the number of annotated coding genes in the Ensemble release 99 of the WBcel235 assembly. Representation of overlaps was either in the form of Venn diagrams generated using http://bioinformatics.psb.ugent.be/webtools/Venn/ or in the form of the output of the SuperExactTest package.

### Gene set enrichment analysis

Identification of enriched GO terms or association with tissue-specific expression for the gene sets identified in this study was performed using a tool available on Wormbase (Angeles-Albores et al., 2016, 2018) using a *q*-value threshold of 0.1. The *−*log_10_*q* value is plotted for significant GO terms presented here.

### Motif identification in promoters of genes found to be expressed by TaDa

To identify motifs associated with promoters of genes expressed in seam cells or hypodermis based on RPB-6 TaDa experiments, the respective identified gene sets were converted to NCBI Refseq ID names using SimpleMine. The Refseq IDs list was used as input for the perl script findMotifs.pl of the HOMER v4.11.1 platform (Heinz et al., 2010) using a prefabricated *C. elegans* promoter set and looking for motifs 6, 8 or 10 bp long (options: worm -len 6,8,10) within the 2 kb sequence upstream of the TSS of each gene. The homer-generated positional weight matrices were converted into transfac matrices using the RSAT (Nguyen et al., 2018) Metazoa convert matrix tool (http://rsat.sb-roscoff.fr/convert-matrix_form.cgi) and were imported to Weblogo3 (Crooks et al., 2004) (http://weblogo.threeplusone.com/) for logo drawing.

### RNAi by feeding

Knockdown of seam cell-expressed transcription and chromatin factor genes was performed by feeding animals on lawns of bacteria expressing double-strand (ds)RNA targeting each gene of interest (clones are commercially available from Source Bioscience and are listed in Table S6). Bacteria were grown overnight and then seeded directly onto NGM plates containing 1 μM IPTG (Promega), 25 μg/ml ampicillin (Sigma-Aldrich) and 6.25 μg/ml tetracycline (Sigma-Aldrich). Five L4 animals of the JR667 strain were transferred on RNAi plates and allowed to lay progeny, which were observed for phenotypic effects and scored for terminal seam cell number at the L4 and early-adult stage of the subsequent generation. Embryonic lethal or developmental-arresting treatments were performed post-embryonically by seeding synchronised by bleaching embryos on RNAi plates. Control treatments were performed in parallel, by feeding animals on lawns of the same strain of HT115 bacteria.

### Statistical analysis

Statistical analysis for comparisons between quantitative datasets was performed using GraphPad Prism 7 (www.graphpad.com). To test differences in the mean between seam cell scorings, an unpaired two-tailed *t*-test was performed when the comparison was between two datasets, and a one-way analysis of variance (ANOVA) was performed when multiple datasets were compared. One-way ANOVA was followed by a Dunnett's post-hoc test when the mean of multiple datasets was compared with that of a control. To compare distributions for which density plots are shown, a Kolmogorov–Smirnov two-sided test was performed in R. The significance level used throughout the study was *P*<0.05.

## Supplementary Material

10.1242/develop.199452_sup1 Supplementary information
