# Supplementary material for: Gene expression profiling of epidermal cell types in C. elegans using Targeted DamID
Source: Development. 2021 Sep 3;148(17):dev199452. doi: 10.1242/dev.199452 (PMC7613258; doi:10.1242/dev.199452)
Supplement: Supplementary information [file develop-148-199452-s1.pdf]

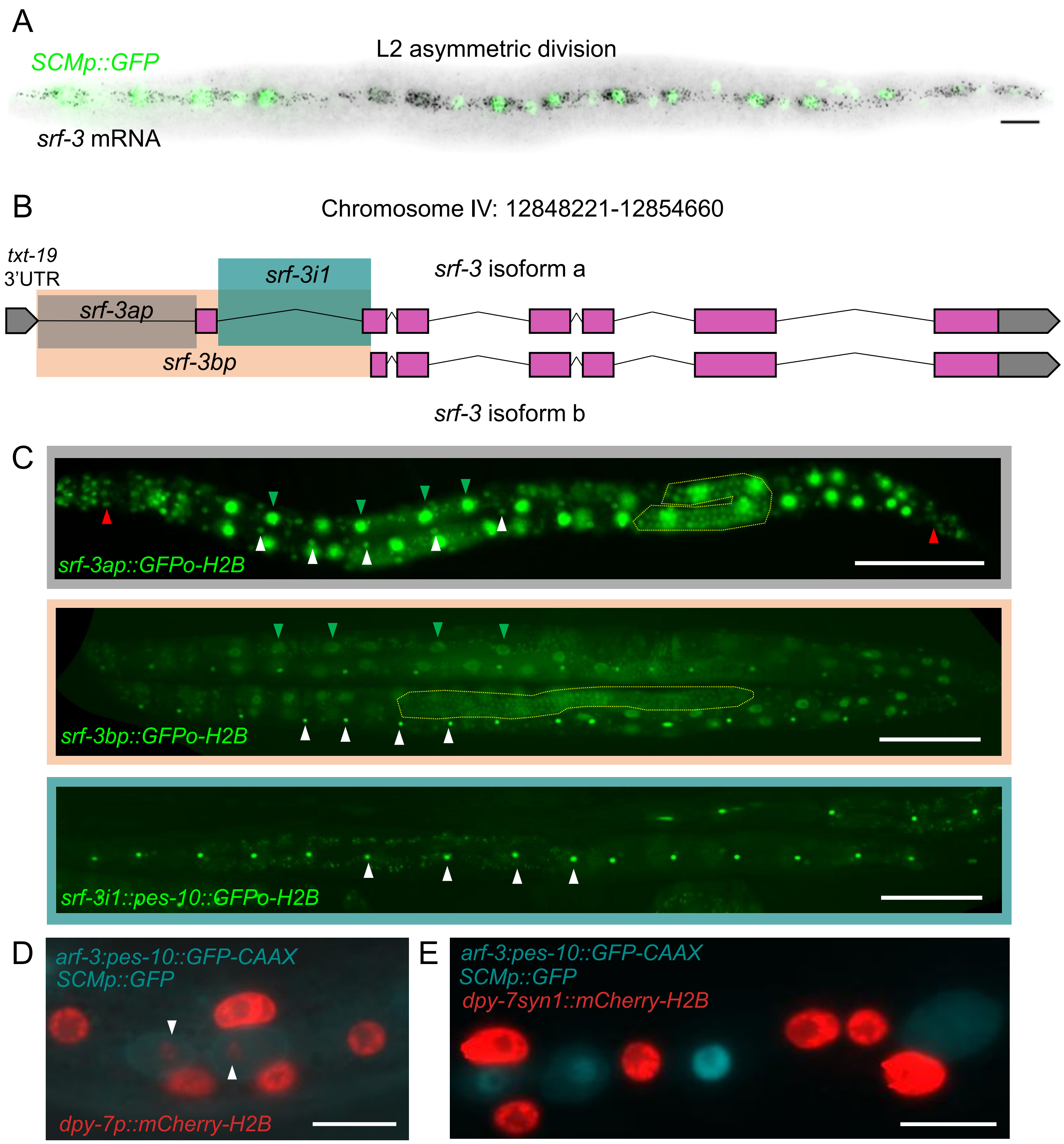
A
SCMp::GFP
L2 asymmetric division
srf-3 mRNA
B
Chromosome IV: 12848221-12854660
txt-19
3'UTR
srf-3i1
srf-3 isoform a
srf-3ap
srf-3bp
srf-3 isoform b
C
srf-3ap::GFPo-H2B
srf-3bp::GFPo-H2B
srf-3i1::pes-10::GFPo-H2B
D
arf-3:pes-10::GFP-CAAX
SCMp::GFP
dpy-7p::mCherry-H2B
E
arf-3:pes-10::GFP-CAAX
SCMp::GFP
dpy-7syn1::mCherry-H2B

**Fig. S1. Establishing seam cell and hypodermis-specific promoters for RNApol TaDa.**

**(A)** Representative image of *srf-3* smFISH at the L2 stage, during the asymmetric seam cell division, showing high levels of transcript in seam cells. Seam cell nuclei are marked by the *SCMp:GFP* reporter and *srf-3* mRNAs appear as black spots. **(B)** Illustration of the *srf-3* locus on chromosome IV. Pink blocks signify exon sequences and grey indicate 3' UTRs. Two isoforms of *srf-3* (isoform a and b) are shown. Shaded areas near the 5' mark putative promoter or regulatory sequences tested for seam cell specific expression. With grey, the 1093 bp putative promoter sequence of isoform a (*srf-3ap*), extending from the end of the upstream gene *txt-19* 3'UTR to the *srf-3* isoform a start codon. With peach, the 2246 bp sequence starting at the same position and extending to the start codon of isoform b (*srf-3bp*) and with teal, the 1081 bp first intron of isoform a (*srf-3i1*). **(C)** Representative fluorescence images of late L4 transgenic animals carrying single-copy transgenes of transcriptional reporters driving expression of *GFP-H2B* under the *srf-3ap* promoter (grey frame), the *srf-3bp* promoter (peach frame) and the *srf-3i1::pes-10* promoter (teal frame). White arrowheads show expression in seam cell nuclei, green in intestinal and red in hypodermal. Yellow outlined areas indicate further expression in the germline. **(D-E)** Representative fluorescence images showing expression of *mCherry-H2B* under the promoter of *dpy-7* from a single-copy transgene (D) and *dpy-7syn1::mCherry-H2B* from a multi-copy transgene (E) at the L3 asymmetric cell division stage. Seam cells are marked in cyan by membrane (*arf-3:pes10::GFP-CAAX*) and nuclear (*SCMp::GFP*) reporters. Note expression of *dpy-7p:mCherry-H2B* in seam cell nuclei, indicated by white arrowheads, which is more prominent during divisions and is abolished in *dpy-7syn1::mCherry-H2B* transgenics. Scale bars are 100 μm in C and 10 μm in D and E.

Development • Supplementary information

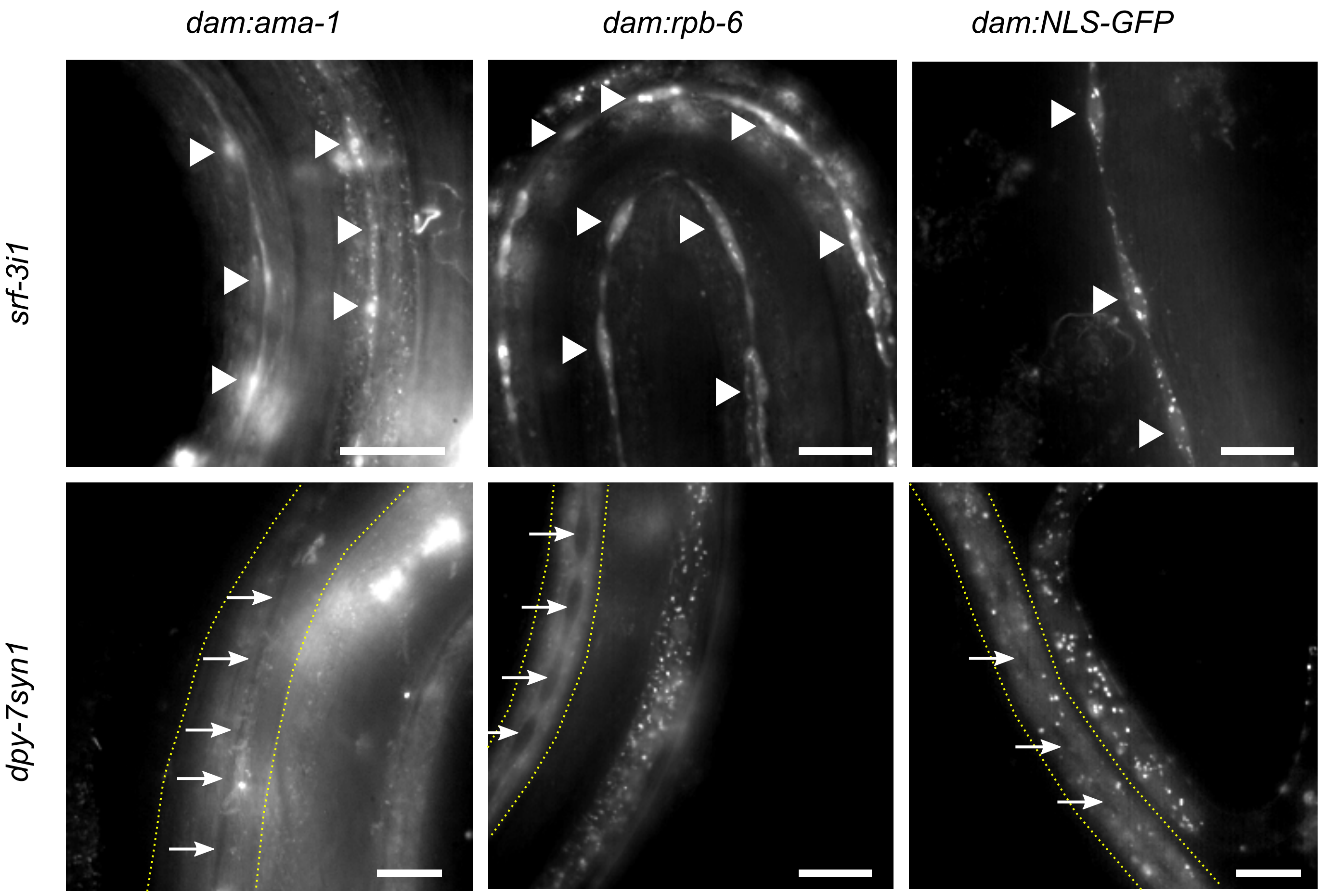


**Fig. S2. Validation of expression of the RNApol TaDa transgenes.**

Confirmation of single-copy transgene expression in the expected tissue of interest, for specified transgenes, using *mCherry* expression as a proxy. Animals were imaged at the L4 stage. White arrowheads indicate expression in the seam cells, while white arrows indicate absence of expression in the seam cells in animals expressing in the hypodermis, indicated by the yellow outline. Scale bars are 20 μm.

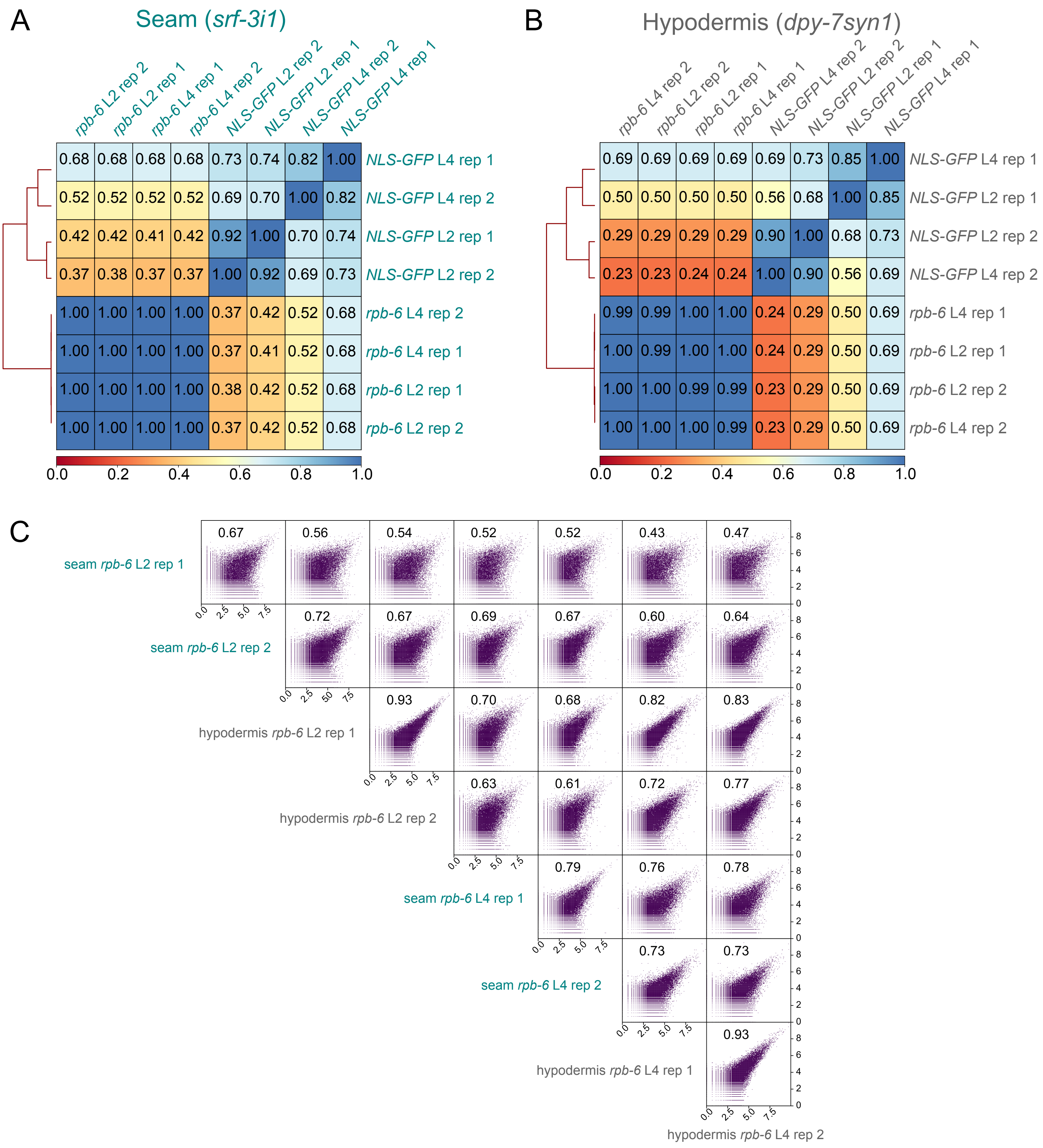


Development • Supplementary information

**Fig. S3. RPB-6 occupancy signatures are similar across cell types and different to controls.**

**(A-B)** Pearson correlation heatmaps based on genome-wide normalised aligned read count maps for the seam (A) and hypodermis (B). The correlation coefficient for each pairwise comparison is printed in each cell of the heatmap. For both promoters, the *dam:rpb-6* samples show higher correlation than the control samples. **(C)** Scatterplots and Pearson correlations across replicates, cell types and developmental stages based on read-count normalised scores of GATC fragments participating in protein-coding genes. The correlation coefficient *r* values are shown in each corresponding cell. Note that these values are not identical to those reported in Fig. 2B, likely due to differences in signal distribution across GATC fragments in protein-coding genes.

Development • Supplementary information

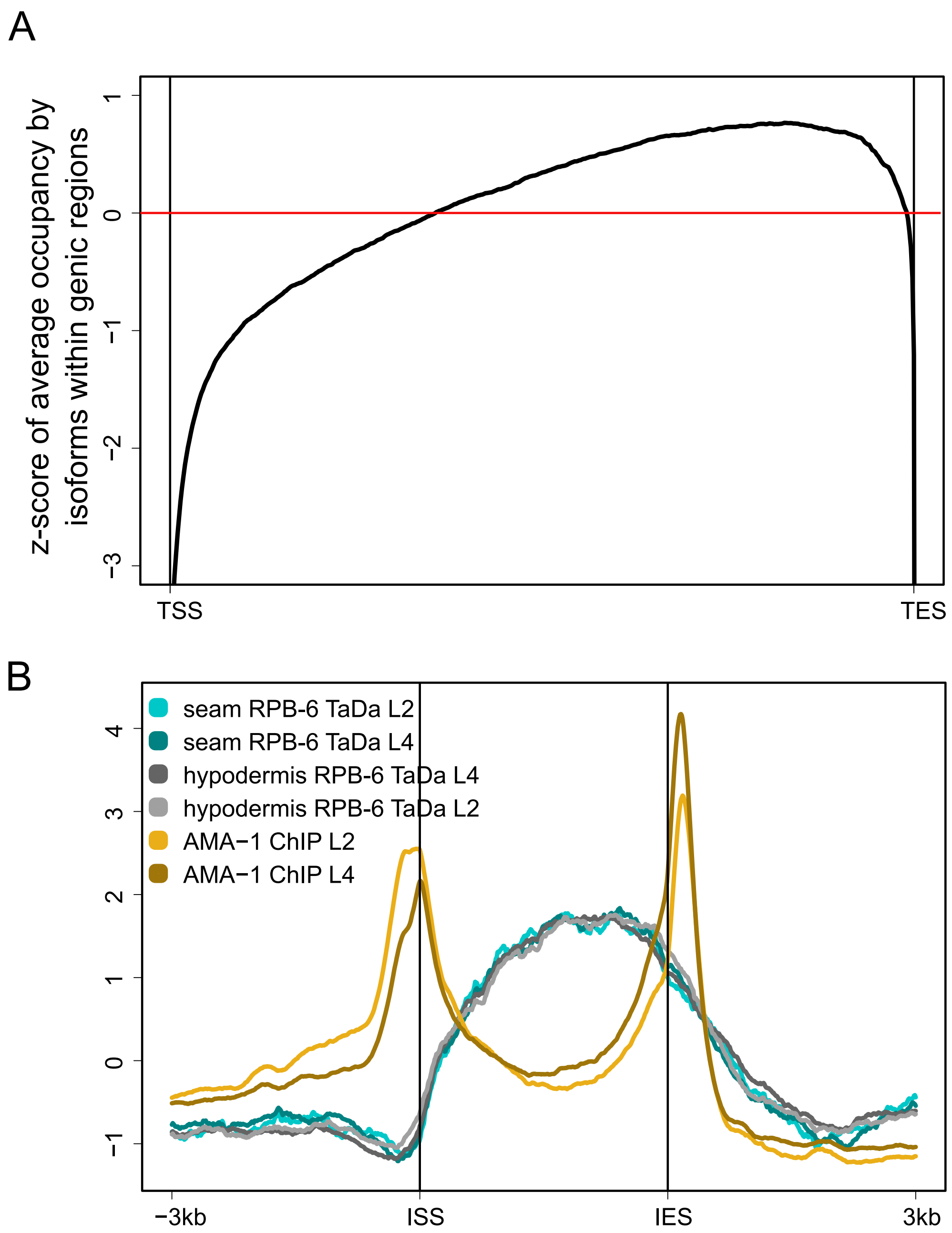


**Fig. S4. Isoform positional enrichment within genes does not explain the RPB-6 occupancy signal preference for 3' regions.**

**(A)** Deviation from the average occupancy of gene sequences by isoforms shows increased coverage of 3' regions compared to 5' regions of gene sequences annotated based on the largest transcript. Red line indicates the average isoform occupancy across the gene length. **(B)** Aggregation plot for the RPB-6 TaDa signal from the different cell types and developmental stages along with whole-animal AMA-1 ChIP-seq signal with the averaged signal anchored at the start and end sites (ISS and IES respectively) of all isoforms of *C. elegans* protein-coding genes, pushed into a pseudo-length of 3kb, along with a region 3kb upstream and 3kb downstream. In Y-axes show z-scores for the plotted sequence length in both cases.

Development • Supplementary information

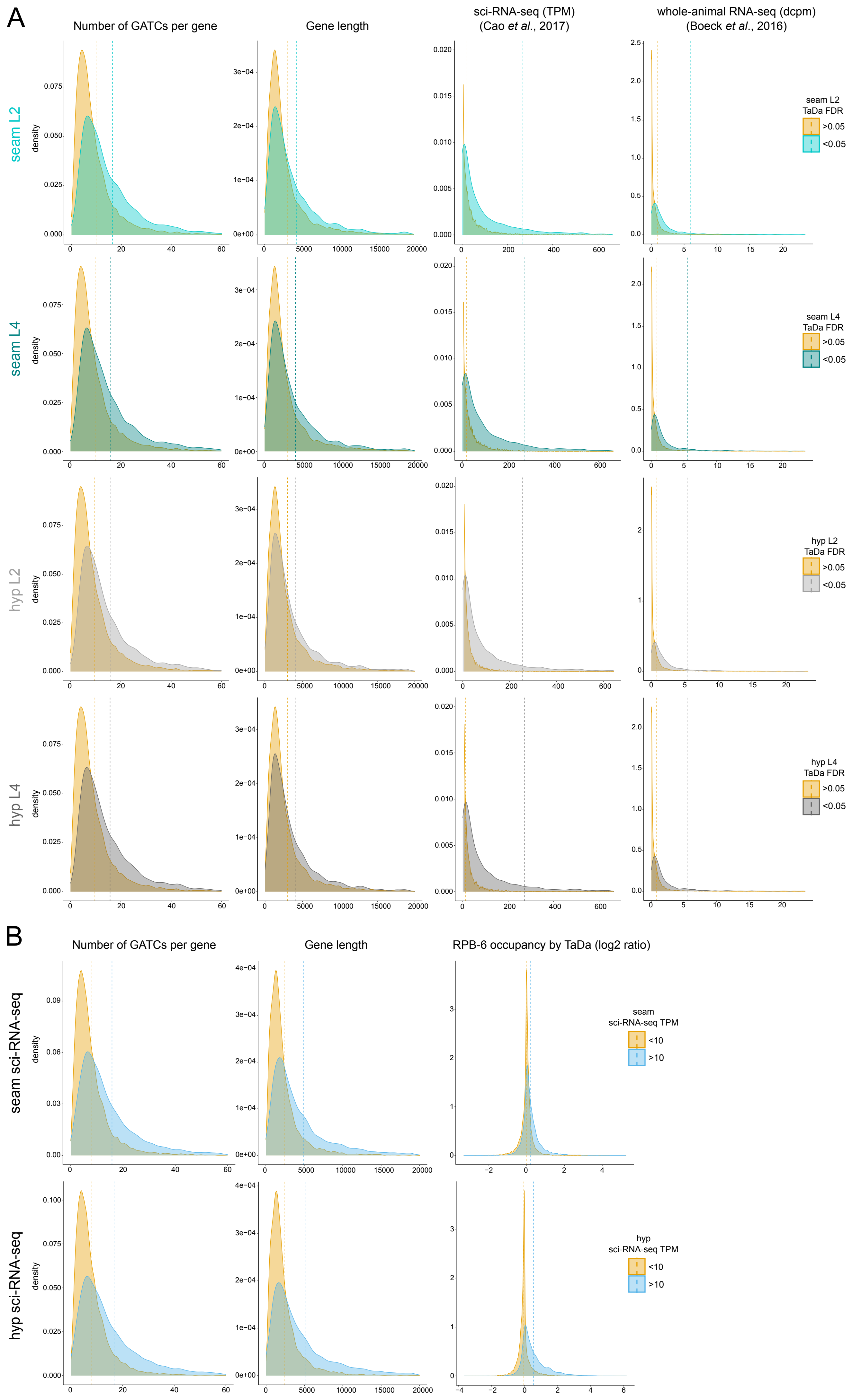


Development • Supplementary information

**Fig. S5. TaDa-identified expressed genes show higher expression in RNA-seq experiments and are biased for GATC availability.**

**(A)** Density plots of protein coding genes, separated in groups of expressed or non-expressed based on TaDa (FDR<0.05), per cell type and developmental stage plotted against: the number of GATCs within gene, gene length, sci-RNA-seq expression values (in TPM: transcripts per million) (Cao et al., 2017) and stage-matched whole-animal RNA-seq expression values (in dcpm: depth of coverage per base per million reads) (Boeck et al., 2016). For all cell types and stages TaDa expressed genes showed significantly increased sci-RNA-seq and whole-animal RNA-seq expression values compared to non-expressed, as well as higher numbers of GATCs and consequently longer gene lengths. **(B)** Density plots of protein coding genes, separated in groups of expressed or non-expressed based on sci-RNA-seq (TPM>10) (Cao et al., 2017), per cell type plotted against: the number of GATCs within gene, gene length and TaDa RPB-6 occupancy values ($\log_2$(*rpb-6:dam/NLS-GFP:dam*)). In both cell types expressed genes showed significantly longer gene length and likely consequently more GATCs than non-expressed, as well as higher RPB-6 occupancy values. In A and B dashed lines indicate the distribution mean and statistical significance of difference in the distributions was determined with a Kolmogorov-Smirnov test, $p < 2.2\times10^{-16}$. The X-axis is limited to the 99th percentile of values and for sci-RNA-seq the Y-axis is limited to 0.02 for visualisation.

Development • Supplementary information

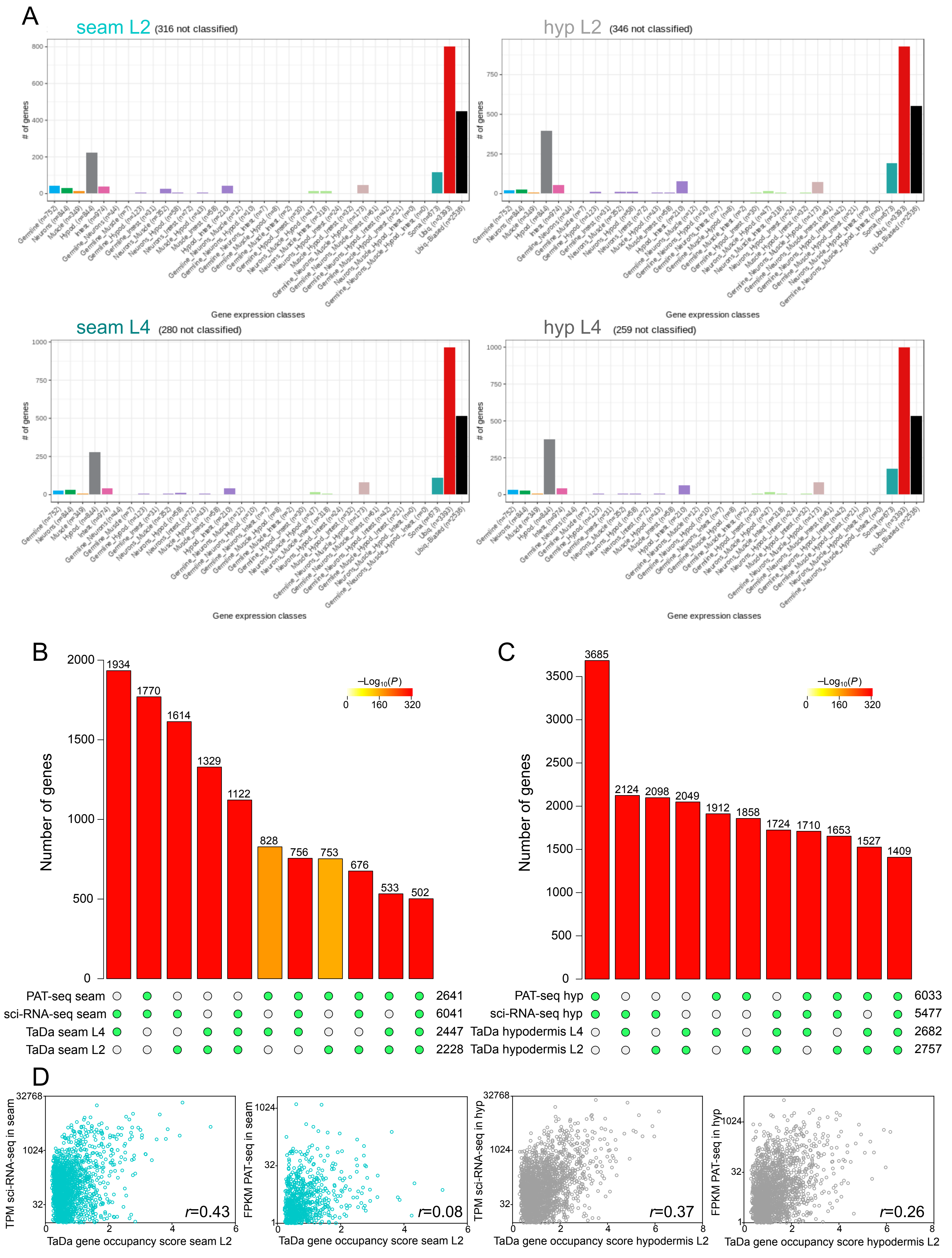


Development • Supplementary information

**Fig. S6. TaDa-identified expressed genes overlap significantly with other published transcriptomes.**

**(A)** Barplots of numbers of TaDa-identified expressed genes for each cell type and developmental stage that are in common with gene-sets classified based on their tissue specificity (Serizay et al., 2020). **(B-C)** Barplots of the sizes and statistical significance, assessed by a Fisher's exact test, of all possible intersections between TaDa, sci-RNA-seq genes over a 10 TPM threshold (Cao et al., 2017) and PAT-seq-identified sets of expressed genes (Blazie et al., 2017) in the seam cells (B) and the hypodermis (C). All overlaps are highly significant. **(D)** Correlation scatterplots of expression levels for L2 genes common between seam (left) or hypodermis (right) TaDa sets and the sci-RNA-seq or the PAT-seq datasets for seam cells and hypodermis. For TaDa the $\log_2$*(dam:rpb-6/dam:NLS-GFP)* scores are used as a measure of expression levels, for sci-RNA-seq the values are transcripts per million reads (TPM) and for PAT-seq fragments per kilobase of transcript per million reads (FPKM). All correlation analyses showed significant although weak correlation (indicated by the $r$ values) across methods with $p$<0.0001 for all.

Development • Supplementary information

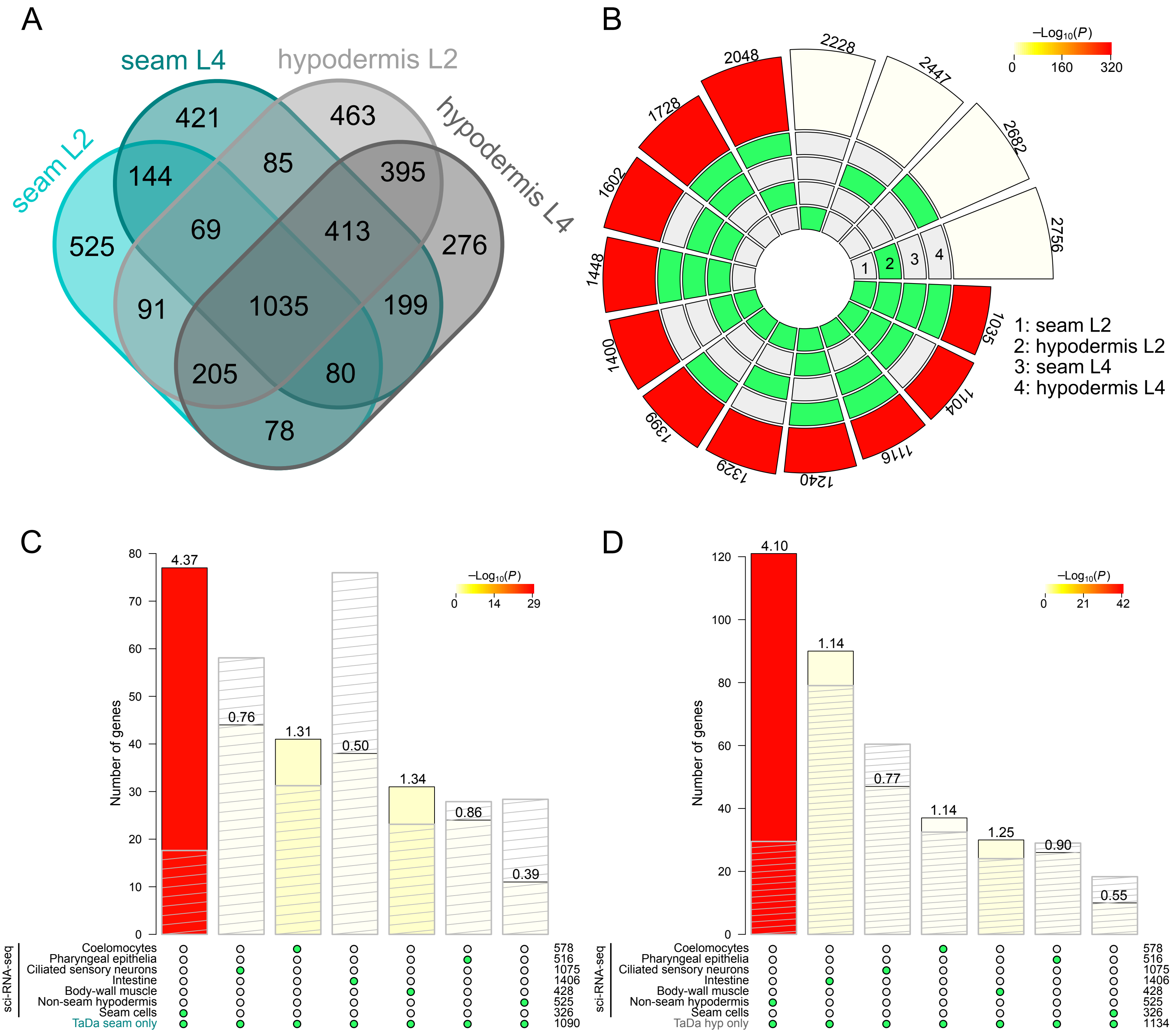
A
seam L4
hypodermis L2
seam L2
hypodermis L4
421
463
144
85
395
525
69
413
276
91
1035
199
205
80
78
B
–Log10(P)
0
160
320
2048
2228
2447
2682
2756
1728
1602
1448
1400
1399
1329
1240
1116
1104
1035
1: seam L2
2: hypodermis L2
3: seam L4
4: hypodermis L4
C
Number of genes
–Log10(P)
0
14
29
4.37
0.76
1.31
0.50
1.34
0.86
0.39
sci-RNA-seq
Coelomocytes
Pharyngeal epithelia
Ciliated sensory neurons
Intestine
Body-wall muscle
Non-seam hypodermis
Seam cells
TaDa seam only
578
516
1075
1406
428
525
326
1090
D
Number of genes
–Log10(P)
0
21
42
4.10
1.14
0.77
1.14
1.25
0.90
0.55
sci-RNA-seq
Coelomocytes
Pharyngeal epithelia
Ciliated sensory neurons
Intestine
Body-wall muscle
Non-seam hypodermis
Seam cells
TaDa hyp only
578
516
1075
1406
428
525
326
1134

**Fig. S7. Intersections of TaDa-identified expressed gene sets show significant overlap across cell types and reveal seam cell and hypodermal-specific genes.**
**(A-B)** Multiple intersections of all the acquired gene-sets expressed in the seam and hypodermis in both stages. The Venn diagram (A) presents the number of genes that are shared across datasets or are unique to the cell type and/or developmental stage. The circular plot (B) reports the sizes of all pairwise and higher-order intersections between the sets (indicated in green whether they are included in each comparison) and indicates that they are highly significant (in red) with a Fisher's exact test. **(C-D)** Barplots representing intersections between TaDa seam-only (C) or TaDa hypodermis-only (D) gene sets and cell-type-specific genes from 7 non-related cell types from sci-RNA-seq (genes with a TPM>10 and 5-fold higher than the $2^{nd}$ most expressing cell type) (Cao et al., 2017). The colour of the bar signifies statistical significance assessed by a Fisher's exact test, gridded bars indicate expected overlap based on gene set and genome size and the enrichment fold between observed and expected overlaps is printed on top of bars. The most significant intersections are those between gene sets specific for the same-cell type ($p \leq 7.06 \times 10^{-29}$).

Development • Supplementary information

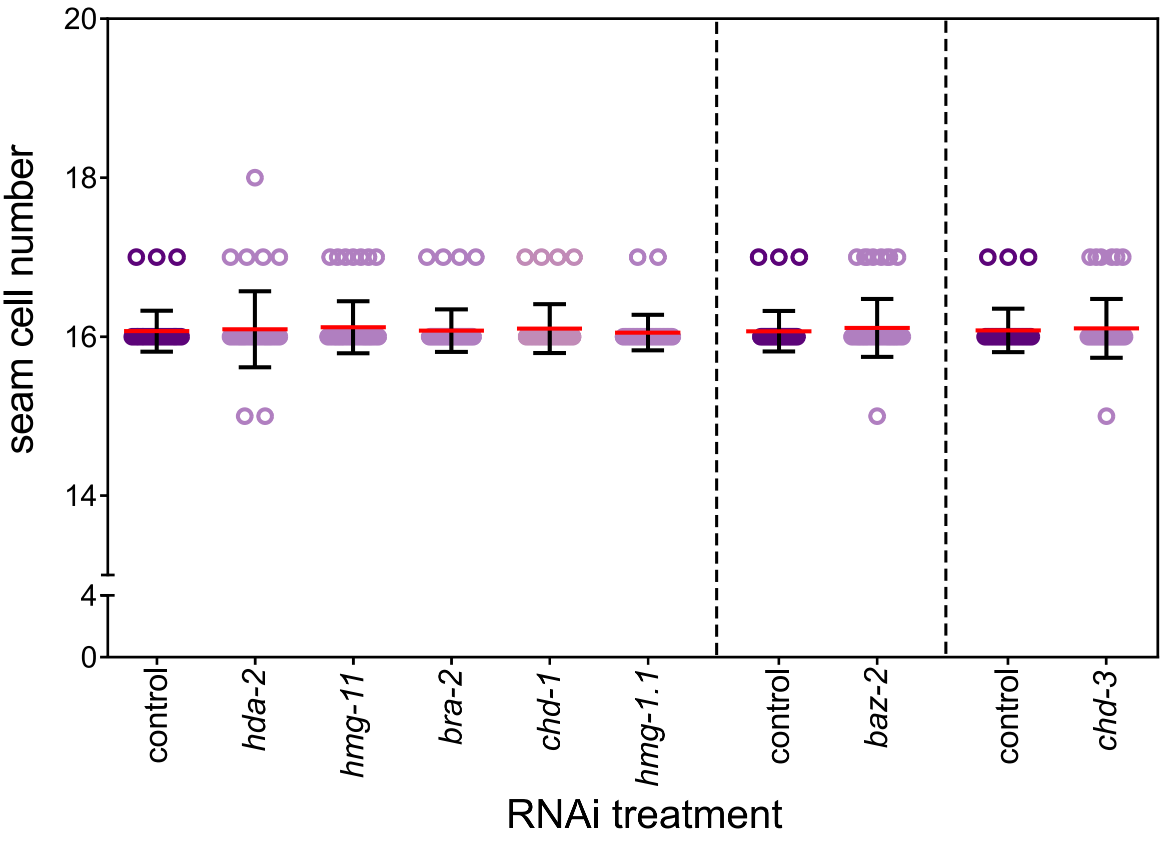


**Fig. S8. RNAi-screen for putative chromatin factors regulating seam cell patterning.** Quantification of seam cell number at the late L4 stage of RNAi treated animals carrying the *SCMp::GFP* reporter in a WT background (39≤n≤63 animals per treatment). Targeted chromatin factors are indicated on the X-axis and sets of treatments performed on the same day are separated from others by dashed lines, having their respective control. No significant changes to the mean or variance were observed in both backgrounds for the presented treatments.

Development • Supplementary information

**Table S1.** List of genes expressed in seam cells and hypodermis at L2 and L4 by TaDa and their overlap with sci-RNAseq and PAT-seq.

Click here to download Table S1

**Table S2.** List of genes (including transcription and chromatin factors) expressed in seam cells at L2 and L4 by TaDa.

Click here to download Table S2

Development • Supplementary information

**Table S3.** List of strains used in this study.

| Strain | Background | Genotype |
|---|---|---|
| N2 | N2 | wild isolate *C. elegans* from Bristol, UK |
| JR667 | N2 | *unc-119(e2498::Tc1) III; wIs51[SCMp::GFP + unc-119(+)] V* |
| EG6699 | N2 | *ttTi5605 II; unc-119(ed3) III; oxEx1578.* |
| MBA250 | N2 | *icbIs2[arf-3::pes-10::GFP:CAAX::unc-54] I; icbSi2[dpy-7p::mCherry:H2B::unc-54 3'UTR+cb-unc-119] IV; wIs51[SCMp::GFP+unc-119(+)] V* |
| MBA467 | N2 | *icbSi25[pDK16(srf-3ap::GFPo-H2B::unc-54 3'UTR + cb-unc-119)] II; unc-119(ed3) III* |
| MBA468 | N2 | *icbSi26[pDK1(psrf-3ap::GFPo-H2B::unc-54 3'UTR + cb-unc-119)] II; unc-119(ed3) III* |
| MBA488 | N2 | *icbEx121[pDK18(dpy-7syn1::mCherry-H2B::unc-54 3'UTR) pBJ36, pRF4]; icbIs2[arf-3::GFP:CAAX::unc-54 3'UTR]I; wIs51[SCMp::GFP + unc-119(+)] V* |
| MBA489 | N2 | *icbEx122[pDK18(dpy-7syn1::mCherry-H2B::unc-54 3'UTR) pBJ36, pRF4]; icbIs2[arf-3::GFP:CAAX::unc-54 3'UTR]I; wIs51[SCMp::GFP + unc-119(+)] V* |
| MBA490 | N2 | *icbEx123[pDK18(dpy-7syn1::mCherry-H2B::unc-54 3'UTR) pBJ36, pRF4]; icbIs2[arf-3::GFP:CAAX::unc-54 3'UTR]I; wIs51[SCMp::GFP + unc-119(+)] V* |
| MBA496 | N2 | *icbSi32[pDK26(srf-3bp::GFPo-H2B::unc-54 3'UTR +cb-unc-119)] II; unc-119(ed3) III* |
| MBA497 | N2 | *icbSi33[pDK26(srf-3bp::GFPo-H2B::unc-54 3'UTR +cb-unc-119)] II; unc-119(ed3) III* |
| MBA540 | N2 | *icbSi42[pDK32(srf-3i1::pes-10::GFPo-H2B::unc-54 3'UTR + cb-unc-119)] II; unc-119(ed3) III* |
| MBA541 | N2 | *icbSi43[pDK32(srf-3i1::pes-10::GFPo-H2B::unc-54 3'UTR + cb-unc-119)] II; unc-119(ed3) III* |
| MBA542 | N2 | *icbSi44[pDK32(srf-3i1::pes-10::GFPo-H2B::unc-54 3'UTR + cb-unc-119)] II; unc-119(ed3) III* |
| MBA687 | N2 | *icbSi71[pDK62(cb-unc-119 + dpy-7syn1::wormCherry::Dam-myc:ama-1::unc-54 3'UTR)] II; unc-119(ed3) III* |
| MBA688 | N2 | *icbSi72[pDK65(dpy-7syn1::wormCherry::Dam-myc:rpb-6::unc-54 3'UTR + cb-unc-119)] II; unc-119(ed3) III* |
| MBA692 | N2 | *icbSi76[pDK54(srf-3i1::pes-10::wormCherry::Dam-myc:NLS-GFP::unc-54 3'UTR + cb-unc-119)] II; unc-119(ed3) III* |
| MBA693 | N2 | *icbSi77[pDK64(dpy-7syn1::wormCherry::Dam-myc:NLS-GFP::unc-54 3'UTR + cb-unc-119)] II; unc-119(ed3) III* |
| MBA694 | N2 | *icbSi78[pDK46(cb-unc-119 + srf-3i1::pes-10::wormCherry::Dam-myc:ama-1::unc-54 3'UTR)] II; unc-119(ed3) III* |
| MBA698 | N2 | *icbSi82[pDK55(srf-3i1::pes-10::wormCherry::Dam-myc:rpb-6::unc-54 3'UTR + cb-unc-119)]; unc-119(ed3) III* |
| MBA1138 | N2 | *icbEx261[pDK133(srf-3i1::Δpes-10::mir-42-44::p10 3'UTR), myo-2::dsRed, pBJ36]; unc-119(e2498::Tc1) III; wIs51[SCMp::GFP + unc-119(+)] V* |
| MBA1139 | N2 | *icbEx262[pDK133(srf-3i1::Δpes-10::mir-42-44::p10 3'UTR), myo-2::dsRed, pBJ36]; unc-119(e2498::Tc1) III; wIs51[SCMp::GFP + unc-119(+)] V* |
| MBA1140 | N2 | *icbEx263[pDK133(srf-3i1::Δpes-10::mir-42-44::p10 3'UTR), myo-2::dsRed, pBJ36]; unc-119(e2498::Tc1) III; wIs51[SCMp::GFP + unc-119(+)] V* |
| MBA1141 | N2 | *icbEx267[pDK147(dpy-7syn1::mir-42-44::p10 3UTR), myo-2::dsRed, pBJ36]; unc-119(e2498::Tc1) III; wIs51[SCMp::GFP + unc-119(+)] V* |
| MBA1142 | N2 | *icbEx265[pDK139(srf-3i1::Δpes-10:::mir-47::p10 3'UTR), myo-2::dsRed, pBJ36]; unc-119(e2498::Tc1) III; wIs51[SCMp::GFP + unc-119(+)] V* |
| MBA1143 | N2 | *icbEx266[pDK139(srf-3i1::Δpes-10:::mir-47::p10 3'UTR), myo-2::dsRed, pBJ36]; unc-119(e2498::Tc1) III; wIs51[SCMp::GFP + unc-119(+)] V* |
| MBA1144 | N2 | *icbEx264[pDK139(srf-3i1::Δpes-10:::mir-47::p10 3'UTR), myo-2::dsRed, pBJ36]; unc-119(e2498::Tc1) III; wIs51[SCMp::GFP + unc-119(+)] V* |
| MBA1145 | N2 | *icbEx268[pDK148(dpy-7syn1::mir-47::p10 3UTR), myo-2::dsRed, pBJ36]; unc-119(e2498::Tc1) III; wIs51[SCMp::GFP + unc-119(+)] V* |
| MBA1146 | N2 | *icbEx269[pDK148(dpy-7syn1::mir-47::p10 3UTR), myo-2::dsRed, pBJ36]; unc-119(e2498::Tc1) III; wIs51[SCMp::GFP + unc-119(+)] V* |
| MBA1192 | N2 | *icbEx292[pDK158(srf-3i1-mut::Δpes-10::outron::>hda-1 fragment>::srf-3a intron5::<hda-1 fragment<::p10 3UTR), myo-2::dsRed, pBJ36]]; wIs51[SCMp::GFP + unc-119(+)] V* |

Development • Supplementary information

**Table S4.** Primers used in this study (general oligos and smFISH probes).

| Oligo name | Sequence 5' -> 3' |
|---|---|
| DK27 | CTCATCTCTGAAGAGGATCTGCGGCATGGAATGGCCGATGAGGACGATTATC |
| DK28 | CGAGATGGCTAGCACTTAATTAACTGCATGCTACCAATCAGCGAGTTGAA |
| DK33 | CTTACTTGCACTTATAATACGACTCACTAGGCTAGCTTTTCACTTTGGTTTTCACG |
| DK34 | TTCTCCTTTACTCATCCTAGGATTCCAGATGGTACCTGAAA |
| DK35 | GGTACCATCTGGAATCCTAGGATGAGTAAAGGAGAAGAATT |
| DK36 | CCGATCCCCCGGGCACTTAAGCTTGTAGAGCTCGTCCATTC |
| DK37 | GACGAGCTCTACAAGCTTAAGTGCCCGGGGGATCGGTGGAG |
| DK38 | CACGGGCGCGAGATGTTAATTAATTACTTGCTGGAAGTGTACT |
| DK39 | ACTTCCAGCAAGTAATTAATTAACATCTCGCGCCCGTGCCTCT |
| DK40 | GGCCCGGGCTACGTAATACGACTCACTTAAAAACAGTTATGTTTGGTATA |
| DK41 | AGGCCTTGACTAGAGGGTACCAGAGCTCACCAACTTTGTATAGAAAAGTT |
| DK42 | CCACTTCAAAAAAGCGCGATTTTTCTTCATCCTAGGGTTACTACTTATAC |
| DK43 | CTCATCTCTGAAGAGGATCTGCGGCATGCTATGACTGCTCCAAAGAAGAA |
| DK44 | GCGAGATGGCTAGCACTTAATTAATGCATGCTATTTGTATAGTTCATCCA |
| DK46 | ACATTTTGTTCTTGATAAGTTTAAACATTTATGGTCTCAAAGGGTGAAGA |
| DK47 | GTAATTGGACTTAGAAGTCAGAGGCAATTTTTACTTGCTGGAAGTGTACT |
| DK48 | GGGGACAAGTTTGTACAAAAAAGCAGGCTATGGTCAGCAAGGGAGAGGCA |
| DK49 | CTTGTAGAGCTCGTCCATTCCT |
| DK50 | AGGAATGGACGAGCTCTACAAGTGCCCGGGGGATCGGTGGAGC |
| DK59 | AATTCTTCTCCTTTACTCATCCTAGGATTTCCTTTTGATTGTGAAGATTC |
| DK64 | TTGCACTTATAATACGACTCACTAGGCTAGGTGAGTTCTTAACTTAACCT |
| DK65 | CGGGGATCCTCTAGAGTCGACCAAGGCCTGATTTCCTTTTGATTGTGAAG |
| DK66 | GGCCGTGAATCTTCACAATCAAAAGGAAATCAGGCCTTGGTCGACTCTAG |
| DK67 | TGAACAATTCTTCTCCTTTACTCATCCTAGCTCCAAGCAAGGGTCCTCCT |
| DK89 | GGGGACAACTTTGTATAGAAAAGTTGGTGAGTTCTTAACTTAACCT |
| DK90 | GGGGACTGCTTTTTTGTACAAACTTGCTCGAGCTCCAAGCAAGGGTCCTCCT |
| DK102 | CTCATTGACAATCAACCTGCCAATTGTTTCTCGGATGACCTAGGATGACGTCATTCCTGTGCTC |
| DK103 | GTAAGTCTTAACAAAAGTTCAATTGGCTCGATCAAGTAAAATTT |
| DK107 | GAACAATTCTTCTCCTTTACTCATCCTAGGCTGAAAGTTAAAAATTACAG |
| DK113 | GGGGACAACTTTGTATAGAAAAGTTGCGGGAATTCCTTTGATAACG |
| DK114 | GGGGACTGCTTTTTTGTACAAACTTGTTATCAAGAACAAAATGTAA |
| DK217 | TTATACTGTAATTTTTAACTTTCAGCCTAGTTCGCGGACCTTTGTGGGTG |
| DK218 | ATTTGTTATTTTAAAAACGATTCATTTAATATAAACCTTACAGGAACGGAAATAC |
| DK219 | TATTCTTACATTTTGTTCTTGATAACCTAGTTCGCGGACCTTTGTGGGTG |
| DK220 | TTATACTGTAATTTTTAACTTTCAGCCTAGGAGAGCCGACTGAAACTGAA |
| DK221 | ATTTGTTATTTTAAAAACGATTCATTTAATGATTACCGCACACCTTTGCC |
| DK222 | TATTCTTACATTTTGTTCTTGATAACCTAGGAGAGCCGACTGAAACTGAA |
| DK247 | TCGAAGACTAAGGTATGACTCGTTTTCACAGCGAC |
| DK248 | TCGAAGACTAAAGCTCGGTGATTCCATCGCGAAGT |
| MB100 | ATCGGGAGGCGAACCTAACTG |
| MB106 | AGGCAGAATGTGAACAAGACTCG |
| MB270 | CGGGAATTCCTTTGATAACGATAATTGGGTTATTGTGTC |
| MB271 | CGGGAATTCTCATTTATCACACCCTGTTCTT |

| *efl-3* probe (1:50) | | *srf-3* probe (1:50) | |
|---|---|---|---|
| agagccgaagatagttgtca | cgggattttctttgttccaa | atcaaaattgccgtcttcat | atccgacgaatggagattca |
| ccgggtggaatattttcttt | ggttcaggaccacagtaaac | actattctgtagtgttaacc | catgctacgacgacggctac |
| tctggatctgttgttcgatt | aaacatcgaagcttccggtt | tcacgagctcgagaatatct | tccagcaaatccagaaagac |
| ctggagatggtcttggaaca | gtgaactactcagaaggcgt | gtactaacaaacatggcgtc | tcctttgagaatcttttcga |
| gaagcatcggagagttgtga | caattttgtcagtctgtgct | cttcagttaaccaaacagct | tccaaagagatactggtgct |
| gtcacttctagatcgtcatc | ttttgttctgagagcgttct | ctcttcttgagctactaaga | actgccatttgaacatttct |
| cccaaactcttttcttttct | tgcgatttttggagtattcc | gcgtggatgaatcttctagg | atcgccgaaaatgatgctgg |
| ctgtctcttcattgatagca | tggtgatgatctgacagctg | ctctaggatttgagttcgta | cagttttcgaatcctgcata |
| attcattttccttgcaacgg | ccacctctgcgaacataaac | ctttcaatgtgtcatagggt | ccatacaacaatccatactc |
| cgtagattcgtcgtttttca | tatctcattttctcagctgc | tgtagatcatggcgggaatg | gtaccaaagtacagtaagcc |
| ttgttagttttctgcatggc | attgatgctactgatcccaa | ctgccacgtagaaaagatta | caacagagagacctccaaca |
| aagagactctaatccttgcc | gatgtcattggatgctgtga | gttgcggcgtctaaatgtga | tcggcgtacttgatgcaaac |
| ctctggaagaccttcttcaa | ggaagttgaagaggagccat | aagctgtgatgttatcatga | ggcaaaattcttggcaatgt |
| cacgtggtgatgacaactca | ggctttggaatctctggaag | gtgaaaatagccgccgtgaa | agagaatgatggcgactgaa |
| gagccgacaatatccttgaa | tgtaaactgcttttgagcct | agagatctgcgcaagatgat | aggaaaatggatccgattgt |
| ggtcggtgttgaagtacatg | attgaatcgaggcctagacg | tgcgaaccattgtgttcgat | aaatgacaagtgaggcgccg |
| acgagtgtcaactctcgatc | ttgcacaggtgtatagtcag | ctccgacaaataatacagct | caccatcgactgatgagatg |
| tcaaaaaccgtctgcacagc | agatacgagtgggcgtattg | ccttgaagttgaacgagaga | acggaatctcgccacgtaat |
| cttttcggatattcttcggg | gttgcgaagattcttgtgga | agatgattcctttgcttttg | agacaaaacgcctcttttgt |
| aactgtgctagctacatcga | aatgttttggcttgacgtct | | |
| aaaaccttctgtttccggat | cttcgattcgccaagaatgt | | |
| tataagcgtctgcatcgact | tgtgctcgaacgtattctga | | |
| aaccaggacattggcaatgt | cacctggaatgcggaagatg | | |
| accttttaatgagcccaag | ctccgaagactttttttggt | | |

**Table S5.** List of transgenes and injection mixes used in this study.

| Extrachromosomal array number | Genetic contents | Composition of injection mix |
|---|---|---|
| icbEx121 | [pDK18(dpy-7syn1::mCherry-H2B::unc-54 3'UTR) pBJ36, pRF4] | pDK18 10 ng/µl, pRF4 35 ng/µl, pBJ36 80 ng/µl |
| icbEx122 | [pDK18(dpy-7syn1::mCherry-H2B::unc-54 3'UTR) pBJ36, pRF4] | pDK18 10 ng/µl, pRF4 35 ng/µl, pBJ36 80 ng/µl |
| icbEx123 | [pDK18(dpy-7syn1::mCherry-H2B::unc-54 3'UTR) pBJ36, pRF4] | pDK18 10 ng/µl, pRF4 35 ng/µl, pBJ36 80 ng/µl |
| icbEx261 | [pDK133(srf-3i1::Δpes-10::mir-42-44::p10 3'UTR), myo-2::dsRed, pBJ36] | pDK133 50 ng/µl, myo-2::dsRed 5 ng/µl, pBJ36 70 ng/µl |
| icbEx262 | [pDK133(srf-3i1::Δpes-10::mir-42-44::p10 3'UTR), myo-2::dsRed, pBJ36] | pDK133 50 ng/µl, myo-2::dsRed 5 ng/µl, pBJ36 70 ng/µl |
| icbEx263 | [pDK133(srf-3i1::Δpes-10:::mir-42-44::p10 3'UTR), myo-2::dsRed, pBJ36] | pDK133 50 ng/µl, myo-2::dsRed 5 ng/µl, pBJ36 70 ng/µl |
| icbEx264 | [pDK139(srf-3i1::Δpes-10:::mir-47::p10 3'UTR), myo-2::dsRed, pBJ36] | pDK139 50 ng/µl, myo-2::dsRed 5 ng/µl, pBJ36 70 ng/µl |
| icbEx265 | [pDK139(srf-3i1::Δpes-10:::mir-47::p10 3'UTR), myo-2::dsRed, pBJ36] | pDK139 50 ng/µl, myo-2::dsRed 5 ng/µl, pBJ36 70 ng/µl |
| icbEx266 | [pDK139(srf-3i1::Δpes-10:::mir-47::p10 3'UTR), myo-2::dsRed, pBJ36] | pDK139 50 ng/µl, myo-2::dsRed 5 ng/µl, pBJ36 70 ng/µl |
| icbEx267 | [pDK147(dpy-7syn1::mir-42-44::p10 3UTR), myo-2::dsRed, pBJ36] | pDK147 50 ng/µl, myo-2::dsRed 5 ng/µl, pBJ36 70 ng/µl |
| icbEx268 | [pDK148(dpy-7syn1::mir-47::p10 3UTR), myo-2::dsRed, pBJ36] | pDK148 50 ng/µl, myo-2::dsRed 5 ng/µl, pBJ36 70 ng/µl |
| icbEx269 | [pDK148(dpy-7syn1::mir-47::p10 3UTR), myo-2::dsRed, pBJ36] | pDK148 50 ng/µl, myo-2::dsRed 5 ng/µl, pBJ36 70 ng/µl |
| icbEx292 | [pDK158(srf-3i1-mut:delta-pes-10::outron::>hda-1 fragment>::srf-3a intron5::<hda-1 fragment<::p10 3UTR),myo-2::dsRed, pBJ36] | pDK158 50 ng/ul, myo-2::dsRed 5 ng/ul, pBJ36 70 ng/ul |
| | | |
| Single-copy insertion number | Genetic contents | Composition of injection mix |
| icbSi25 | [pDK16(srf-3ap::GFPo-H2B::unc-54 3'UTR + cb-unc-119)] | pDK16 50 ng/µl, pCFJ601 50 ng/µl, pMA122 10 ng/µl, pGH8 10 ng/µl, myo-2;;dsRed 2,5 ng/µl, pCFJ104 5 ng/µl |
| icbSi26 | [pDK16(srf-3ap::GFPo-H2B::unc-54 3'UTR + cb-unc-119I | pDK16 50 ng/µl, pCFJ601 50 ng/µl, pMA122 10 ng/µl, pGH8 10 ng/µl, myo-2;;dsRed 2,5 ng/µl, pCFJ104 5 ng/µl |
| icbSi32 | [pDK26(srf-3bp::GFPo-H2B::unc-54 3'UTR +cb-unc-119)] | pDK26 50 ng/µl, pCFJ601 50 ng/µl, pMA122 10 ng/µl, pGH8 10 ng/µl, myo-2;;dsRed 2,5 ng/µl, pCFJ104 5 ng/µl |
| icbSi33 | [pDK26(srf-3bp::GFPo-H2B::unc-54 3'UTR +cb-unc-119)] | pDK26 50 ng/µl, pCFJ601 50 ng/µl, pMA122 10 ng/µl, pGH8 10 ng/µl, myo-2;;dsRed 2,5 ng/µl, pCFJ104 5 ng/µl |
| icbSi42 | [pDK32(srf-3i1::pes-10::GFPo-H2B::unc-54 3'UTR + cb-unc-119)] | pDK32 50 ng/µl, pCFJ601 50 ng/µl, pMA122 10 ng/µl, pGH8 10 ng/µl, myo-2;;dsRed 2,5 ng/µl, pCFJ104 5 ng/µl |
| icbSi43 | [pDK32(srf-3i1::pes-10::GFPo-H2B::unc-54 3'UTR + cb-unc-119)] | pDK32 50 ng/µl, pCFJ601 50 ng/µl, pMA122 10 ng/µl, pGH8 10 ng/µl, myo-2;;dsRed 2,5 ng/µl, pCFJ104 5 ng/µl |
| icbSi44 | [pDK32(srf-3i1::pes-10::GFPo-H2B::unc-54 3'UTR + cb-unc-119)] | pDK32 50 ng/µl, pCFJ601 50 ng/µl, pMA122 10 ng/µl, pGH8 10 ng/µl, myo-2;;dsRed 2,5 ng/µl, pCFJ104 5 ng/µl |
| icbSi71 | [pDK62(cb-unc-119 + dpy-7syn1::wormCherry::Dam-myc:ama-1::unc-54 3'UTR)] | pDK62 50 ng/µl, pCFJ601 50 ng/µl, pMA122 10 ng/µl, pGH8 10 ng/µl, myo-2;;dsRed 2,5 ng/µl, pCFJ104 5 ng/µl |
| icbSi72 | [pDK65(dpy-7syn1::wormCherry::Dam-myc:rpb-6::unc-54 3'UTR + cb-unc-119)] | pDK65 50 ng/µl, pCFJ601 50 ng/µl, pMA122 10 ng/µl, pGH8 10 ng/µl, myo-2;;dsRed 2,5 ng/µl, pCFJ104 5 ng/µl |
| icbSi76 | [pDK54(srf-3i1::pes-10::wormCherry::Dam-myc:NLS-GFP::unc-54 3'UTR + cb-unc-119)] | pDK54 50 ng/µl, pCFJ601 50 ng/µl, pMA122 10 ng/µl, pGH8 10 ng/µl, myo-2;;dsRed 2,5 ng/µl, pCFJ104 5 ng/µl |
| icbSi77 | [pDK64(dpy-7syn1::wormCherry::Dam-myc:NLS-GFP::unc-54 3'UTR + cb-unc-119)] | pDK64 50 ng/µl, pCFJ601 50 ng/µl, pMA122 10 ng/µl, pGH8 10 ng/µl, myo-2;;dsRed 2,5 ng/µl, pCFJ104 5 ng/µl |
| icbSi78 | [pDK46(cb-unc-119 + srf-3i1::pes-10::wormCherry::Dam-myc:ama-1::unc-54 3'UTR)] | pDK46 50 ng/µl, pCFJ601 50 ng/µl, pMA122 10 ng/µl, pGH8 10 ng/µl, myo-2;;dsRed 2,5 ng/µl, pCFJ104 5 ng/µl |
| icbSi82 | [pDK55(srf-3i1::pes-10::wormCherry::Dam-myc:rpb-6::unc-54 3'UTR + cb-unc-119)] | pDK55 50 ng/µl, pCFJ601 50 ng/µl, pMA122 10 ng/µl, pGH8 10 ng/µl, myo-2;;dsRed 2,5 ng/µl, pCFJ104 5 ng/µl |

**Table S6.** List of RNAi clones used in this study.

| WormBase Gene ID | Public Name | Sequence Name | Ahringer RNAi library Geneservice_location |
|---|---|---|---|
| WBGene00009899 | *efl-3* | *F49E12.6* | II-6K03 |
| WBGene00000275 | *bub-1* | *R06C7.8* | I-3H11 |
| WBGene00001834 | *hda-1* | *C53A5.3* | V-9F11 |
| WBGene00009672 | *F43G9.12* | *F43G9.12* | I-4C12 |
| WBGene00001974 | *hmg-4* | *T20B12.8* | III-3P10 |
| WBGene00010369 | *chd-1* | *H06O01.2* | I-3M20 |
| WBGene00003664 | *nhr-74* | *C27C7.3* | I-5P17 |
| WBGene00001976 | *hmg-11* | *T05A7.4* | II-3N12 |
| WBGene00006554 | *tbx-35* | *ZK177.10* | II-4O24 |
| WBGene00001835 | *hda-2* | *C08B11.2* | II-5N08 |
| WBGene00001971 | *hmg-1.1* | *Y48B6A.14* | II-9G13 |
| WBGene00017757 | *bra-2* | *F23H11.1* | III-1E22 |
| WBGene00001470 | *baz-2* | *ZK783.4* | III-4E11 |
| WBGene00006970 | *zag-1* | *F28F9.1* | IV-1P04 |
| WBGene00003606 | *nhr-7* | *F54D1.4* | IV-6C03 |
| WBGene00020062 | *nhr-270* | *R13D11.8* | V-1B09 |
| WBGene00003649 | *nhr-59* | *T27B7.1* | V-2M07 |
| WBGene00001210 | *egl-46* | *K11G9.4* | V-4L02 |
| WBGene00003717 | *nhr-127* | *T13F3.3* | V-10J08 |
| WBGene00000482 | *chd-3* | *T14G8.1* | X-6G01 |
| WBGene00007433 | *swsn-7* | *C08B11.3* | II-5N10 |
