# Supplementary material for: Gene expression profiling of epidermal cell types in C. elegans using Targeted DamID
Source: Development. 2021 Sep 3;148(17):dev199452. doi: 10.1242/dev.199452 (PMC7613258; doi:10.1242/dev.199452)
Supplement: Supplementary file 1 [file develop-148-199452_review_history.pdf]

Gene expression profiling of epidermal cell types in *C. elegans* using Targeted DamID

Dimitris Katsanos, Mar Ferrando-Marco, Iqrah Razzaq, Gabriel Aughey, Tony D Southall and Michalis Barkoulas
DOI: 10.1242/dev.199452

Editor: Swathi Arur

Review timeline

Original submission:	20 January 2021
Editorial decision:	9 March 2021
First revision received:	8 June 2021
Editorial decision:	12 July 2021
Second revision received:	2 August 2021
Accepted:	5 August 2021

Original submission

First decision letter

MS ID#: DEVELOP/2021/199452

MS TITLE: Gene expression profiling of epidermal cell types in C. elegans using Targeted-DamID

AUTHORS: Dimitris Katsanos, Mar Ferrando-Marco, Iqrah Razzaq, Gabriel Aughey, Tony D Southall, and Michalis Barkoulas

I have now received all the referees' reports on the above manuscript, and have reached a decision. The referees' comments are appended below, or you can access them online: please go to BenchPress and click on the 'Manuscripts with Decisions' queue in the Author Area.

As you will see, the referees express considerable interest in your work, but have some significant criticisms and recommend a substantial revision of your manuscript before we can consider publication. If you are able to revise the manuscript along the lines suggested by the reviewers with attention to quantitative analysis as suggested by reviewer 2 and the experimental analysis suggested by reviewer 1, I will be happy receive a revised version of the manuscript.

We are aware that you may be experiencing disruption to the normal running of your lab that make experimental revisions challenging. If it would be helpful, we encourage you to contact us to discuss your revision in greater detail. Please send us a point-by-point response indicating where you are able to address concerns raised (either experimentally or by changes to the text) and where you will not be able to do so within the normal timeframe of a revision. We will then provide further guidance. Please also note that we are happy to extend revision timeframes as necessary.

Please attend to all of the reviewers' comments and ensure that you clearly *highlight* all changes made in the revised manuscript. Please avoid using 'Tracked changes' in Word files as these are lost in PDF conversion. I should be grateful if you would also provide a point-by-point response detailing how you have dealt with the points raised by the reviewers in the 'Response to Reviewers' box. If you do not agree with any of their criticisms or suggestions please explain clearly why this is so.

© 2021. Published by The Company of Biologists under the terms of the Creative Commons Attribution License (https://creativecommons.org/licenses/by/4.0/).

Reviewer 1

Advance summary and potential significance to field

Katsanos and colleagues present the development of a new method to generate tissue level transcriptomic maps (named TaDa). Their method involves the expression under a specific promoter of an operon including the mCherry open reading frame followed by the dam methylase fused to a general transcription factor with two intervening stop codons and frameshift. Thereby the second part of the operon is rarely translated, which avoids toxicity. Using this method they map at the genome level the occupancy of the RBP-6 (RNAPolII subunit F) relative to gene transcription start and end sites), which allows them to define the gene sets expressed in two epidermal cell types at two different stages (L2 and L4 stages). They further focus on transcription factors, chromatin remodeling factors and microRNAs enriched in the epidermis to examine which ones modulate the stem cell-like lateral seam cell divisions. They thereby identify two transcription factors expressed in seam cells (EFL-3 and TBX-35), four chromatin remodeling factors (BUB-1, F43G9.12, HMG-4 and HDA-1, which acts in seam cells), and four miRNAs one of which acts in a non-autonomous manner. Interestingly, EFL-3 and HDA-1 knockdown affect the mean and variance of seam cell number. This TaDa method identified most of the main transcription factors known to be required for epidermis differentiation, arguing that it is a reliable method.

Altogether the main advance of this manuscript is the development of the TaDa method, and the demonstration that it can effectively identify novel significant genes. The issue tissue-specific transcriptomic maps is a difficult one, and their TaDa method seems very powerful. Another nice feature of this work is that they highlight that some genes are not only important to generate the right number of seam cells, but also to potentially ensure the robustness, since their knockdown affect the variance of this number.

This is a remarkable study, which presents very solid data. Together with their accompanying paper in bioRxiv, I got convinced that the TaDa approach is quite powerful. This must be accepted for publication in Development.

Comments for the author

The authors could perhaps improve the work by testing whether overexpression of mir-42/43/44 changes the expression of any of the genes affecting seam cell number or variance (tbx-35, efl-3, hda-1, bub-1, hmg-4, F43G9.12).

At this stage, I will not argue for testing the six genes mentioned above using bona fide mutations. However, the authors should highlight in the discussion that partial RNAi effect might explain why some of the other genes tested by RNAi seem not to be required for seam cell division or differentiation, and furthermore that a stronger/null allele of the six aforementioned genes might have generated more penetrant effects.

Other: please give the meaning of the abbreviations TSS and TES when it first appears (it is currently shown only late in the Methods section).

Reviewer 2

Advance summary and potential significance to field

Knowledge of tissue- and cell type-specific gene expression patterns is important for our understanding of the development and function of multicellular organisms. In C. elegans, this has been difficult to obtain, since classical dissection tools do not work well, and other approaches such as PAT-seq, INTACT or scRNA seq are either not very reliable or expensive. Katsanos et al. report the adaptation of an alternative approach Targeted-DamID/TaDa to C. elegans, where they use it to analyze the transcriptomes of two distinct epidermal compartments, the stem cell-like seam cells and the postmitotic hypodermal cells. They test some genes that they report to be seam cell-specific for a function in determining seam cell number, as a proxy for a function in “stemness”.

© 2021. Published by The Company of Biologists under the terms of the Creative Commons Attribution License (https://creativecommons.org/licenses/by/4.0/).

The approach reported is interesting and potentially powerful. At this point, however, we feel that the data presented do not provide sufficient validation that the method works as advertised, and the biological insight appears limited as the evidence for a stem cell function of the tested genes is weak.

Comments for the author

Major points:

1. We have a hard time understanding how two quite different biological samples, i.e., L2-stage seam and L4-stage hyp can correlate at r=0.99 (Fig. S3). This is a level of correlation usually difficult to obtain for RNA-seq among biological replicate experiments. It seems even more unexpected given that gene expression patterns in these particular tissues are known to be highly dynamic, with e.g. widespread oscillatory expression of collagen genes (e.g., Hendriks et al., Mol Cell 2014). Hence, even the question of distinct cell-types aside, it is difficult to understand how the samples from different stages can be so similar. One possibility is that TaDa yields a very "static" signature, i.e., once methylation is present, it will stay on for a long time. This clearly affects then the interpretation of TaDa data in general, because it might yield (for instance) a false impression of similarity between the transcriptomes from tissues derived from the same progenitor. We suggest that the authors take a handful of genes that are on early in development but off at a later time to compare their signatures.

2. Even allowing for the possibility of a "static" TaDA signal, we fail to reconcile the high levels of similarities with the fact that the authors report that hundreds of genes can be detected as specifically expressed in only one vs the other sample (e.g., in the comparison from above, >900 genes, according to Fig. S5 and assuming we did not miscount); overall between the two tissues 1090 genes (ll.329-330) – of fewer than 3,000 detected in each sample (ll.242-245). To us, this suggests that correlations are dominated by systematic noise, not by signal. We would like to see scatter plots with pairwise comparisons (between replicates, between stages, between tissues, respectively), specifically for "genic" signal to have some assurance that there is indeed some level of reproducibility in signal, and (for the comparison between tissues or stages) with an indication of which genes (dots in the plot) are called significantly different based on the tests the authors apply.

3. Related to the two points above, based on gels shown in Figure S2B, the authors conclude that the cell-type-specific rpb-6::Dam-fusions for TaDa produce robust methylation (ll. 170-172). It is difficult for us to observe any clear, reproducible patterns, the two replicates seem to differ greatly (for example sfr-3i1::dam::rpb-6 in L2 stage shows no bands or smear in r1 and low intensity bands in r2; same for different intensity of bands, e.g., dpy-7syn1::dam::rpb-6 in L4 stage show high intensity bands in r1 and low intensity bands in r2). Indeed, are the authors not concerned by the strong and discrete bands they observe in several samples? It is our understanding that methylation on thousands of genes should yield a smear instead. Could the authors please comment and explain how they conclude robustness from this experiment.

4. It is somewhat unclear to us what the dam:NLS-GFP control really controls for. The authors suggest that it causes stochastic methylation (l.185-187), but it seems normalization for systematic errors (e.g., DNA regions that get preferentially methylated, or erroneously called as methylated, even without an rbp-fusion) is more important. Yet, given that the gels in Fig. S2 appear mostly devoid of any signal (smear or discrete bands) for this control, we do wonder whether this control can see more than random noise, highly amplified.

5. The authors suggest that the high level of overlap in seam cell vs. hypodermis TaDa signal could reflect a common epidermal nature (ll.326-328). The above concerns about a "static" signal aside, it is unclear why detection of a TaDa signal would signify tissue-specific expression in the first place: conceptually, we would expect to see a signal as long as the gene of interest is expressed in a given cell, irrespective of whether expression occurs in only this tissue (i.e., is specific), or occurs in other, perhaps even all, tissues as well.
Hence, by default, "specificity" can only be demonstrated by intersecting data from different tissues. A statement on similarity would require examination of at least one other tissue, ideally of developmentally "remote" origin.

© 2021. Published by The Company of Biologists under the terms of the Creative Commons Attribution License (https://creativecommons.org/licenses/by/4.0/).

6. Although the authors claim that TaDa data agree overall well with other approaches aimed at obtaining tissue-specific gene expression data, we do not find these data convincing: In Figure S5A, B, the intersections between TaDa and either PAT-seq or sciRNA-seq are shown, with large numbers of intersecting genes apparently meant to show reproducible detection of tissue-specific expression. However, related to the previous point, in the way this is done, it is unclear whether the intersection represent overlap in tissue-specific or merely in broadly/ubiquitously expressed genes. We would argue that the analysis in S5C, if extended as described in the next point, would better serve the purpose. However, if the authors still wish to keep the type of analysis shown in S5A,B, it would be important that they show the overlap among genes called as tissue-specifically expressed in seam and hyp respectively in either data set (i.e., for Cao et al., use the criteria described in that paper, namely TPM cut-off plus ≥5-fold higher level in the highest than the second most highly expressing tissue). As a negative control, this overlap could then also be documented for tissues other than seam and hypodermis, e.g., VPC, rectum, intestine, neurons.

7. The above analysis, even if done properly, will only give a qualitative view of overlap, and suffers from an arbitrary choice of cut-offs. A more relevant analysis is provided in Figure S5C, where a correlation between quantitative signals between TaDa and the other approaches is tested. The authors conclude (ll. 314 – 316) that this reveals significant correlation. While this appears to be technically correct based on the statistical test used, it is also rather misleading, because the authors present a p-value for presence of any correlation rather than an r-value to quantify the extent of correlation. Looking at S5C, the extent of correlation to the current gold standard, sci-RNA-seq, appears minimal. Even for the correlation that is observed, it is unclear whether this reflects a signal of tissue-specificity, for which “cross”-correlations (e.g., seam TaDa vs. hyp sci-RNA-seq and its converse) would be needed.

8. More generally, the number of genes with TaDa signal in any condition appears quite low (~3,000); the authors need to assess whether this set is biased in any particular way (gene length, expression levels based on sci-RNA seq but also whole worm seq, etc.)

9. To test whether transcription or chromatin factors, or miRNAs, identified as tissue-specifically (seam or hyp) expressed function in seam cell development, the authors deplete some by RNAi or over-express them and count whether any changes in seam cell numbers (mean or variance) occur. This seems a rather crude assay, and it certainly falls short of delivering on what from the introduction appears to be the main motivation for establishing TaDa in seam and hyp cells, showing that any of these factors affect stem cell fates. (For instance, the migration of seam cell daughters or fusion with hyp7 could alternatively be affected).
If the authors feel that a more extensive characterization of at least one of these factors is currently out of their reach, they should tone down this point in their introduction and acknowledge the limitation of their seam cell-counting assay explicitly in the discussion.

Minor points
1. Although mature let-7 accumulates indeed mostly in L4, the let-7 gene is transcribed at all stages (e.g., Perales et al., PLoS Genet 2014, van Wynsberghe et al., Nat Struct Mol Biol 2011) and regulation is post-transcriptional, hence, the data in Fig. 6B would appear to argue against rather than in favor of the specificity of TaDa.

2. While the authors state “Moreover, the miRNA cluster mir-42, mir-43 and mir-44 was found to be expressed only in the seam cells in both stages while mir-47, was found to be expressed only in the hypodermis in both stages (Figure 6B), which matches previous reports based on transcriptional reporters (Martinez et al., 2008).”, this is what the reference states: “ Pmir-42-44: dorsal and ventral part of the embryo, including head. Late embryos show complex expression. Strong expression in seam cells and vulva.
Weaker expression in hypodermis. Also seeing in posterior intestine and rectum and dnc.” (our emphasis) and “Pmir-47: Expression detected from late embryos to adults. In embryos, expression is detected on lateral sides and in larval stages on, in all hypodermis, rectum and vulval cells.” (where, admittedly, the phrasing is not very clear but appears to suggest expression in both seam and other hypodermal compartments).

3. In Figure S1D and S1E, the developmental stages of the imaged worms need to be indicated

© 2021. Published by The Company of Biologists under the terms of the Creative Commons Attribution License (https://creativecommons.org/licenses/by/4.0/).

4. In Figure 2A-B, the ordering of the samples in the Pearson correlation heatmap is rather confusing to us; organization of samples from left to right and top to bottom (so that the diagonal of autocorrelation would go from top left to bottom right) would appear more conventional.

5. Please label the y-axis In Figure 2D.

6. In ll. 393-395 the authors state that they overexpress microRNAs in the epidermis. Please explain in the main text how overexpression was achieved.

7. In l. 179, the authors write that they used materials for sequencing from ~2000 individuals, instead in l. 430 they write “unique reads were acquired starting from a moderately-sized population of ~5000 individuals”. Please indicate the right number of individuals used.

Other:
1. The tissue-specific expression of constructs is difficult to appreciate from the images in Figure S2A.
Images at higher magnification would help; but for the future, the authors might do well to use mCherry::H2B or similar to concentrate signal in the nuclei of expressing cells, which will yield a better signal-to-noise ratio for expressing cells. In the specific case of seam vs hyp distinction, we also note that hypodermal nuclei derive from seam cells through asymmetric division. Hence, to demonstrate specificity, it seems preferable to score reporter expression immediately after one of the asymmetric divisions (ideally L2 or early L4), and not in late L4 as currently done. (We do note that since the stability of the reporter may differ from that of the Dam fusion protein, even this piece of evidence may be subject to challenge, but it seems to be the best evidence currently attainable.)

First revision

Author response to reviewers' comments

We thank both reviewers for providing thoughtful comments and suggestions. Here is our point-by-point response to all comments:

Reviewer 1, Advance Summary and Potential Significance to Field:

Katsanos and colleagues present the development of a new method to generate tissue level transcriptomic maps (named TaDa). Their method involves the expression under a specific promoter of an operon including the mCherry open reading frame followed by the dam methylase fused to a general transcription factor with two intervening stop codons and frameshift. Thereby the second part of the operon is rarely translated, which avoids toxicity. Using this method they map at the genome level the occupancy of the RBP-6 (RNAPolII subunit F) relative to gene transcription start and end sites), which allows them to define the gene sets expressed in two epidermal cell types at two different stages (L2 and L4 stages). They further focus on transcription factors, chromatin remodeling factors and microRNAs enriched in the epidermis to examine which ones modulate the stem cell- like lateral seam cell divisions. They thereby identify two transcription factors expressed in seam cells (EFL-3 and TBX-35), four chromatin remodeling factors (BUB-1, F43G9.12, HMG-4 and HDA-1, which acts in seam cells), and four miRNAs one of which acts in a non-autonomous manner. Interestingly, EFL-3 and HDA-1 knockdown affect the mean and variance of seam cell number. This TaDa method identified most of the main transcription factors known to be required for epidermis differentiation, arguing that it is a reliable method.

Altogether the main advance of this manuscript is the development of the TaDa method, and the demonstration that it can effectively identify novel significant genes. The issue tissue-specific transcriptomic maps is a difficult one, and their TaDa method seems very powerful. Another nice feature of this work is that they highlight that some genes are not only important to generate the right number of seam cells, but also to potentially ensure the robustness, since their knockdown

© 2021. Published by The Company of Biologists under the terms of the Creative Commons Attribution License (https://creativecommons.org/licenses/by/4.0/).

affect the variance of this number.

This is a remarkable study, which presents very solid data. Together with their accompanying paper in bioRxiv, I got convinced that the TaDa approach is quite powerful. This must be accepted for publication in Development.

We thank the reviewer for their summary and comments.

Reviewer 1. Comments for the Author:
The authors could perhaps improve the work by testing whether overexpression of mir- 42/43/44 changes the expression of any of the genes affecting seam cell number or variance (tbx-35, efl-3, hda-1, bub-1, hmg-4, F43G9.12).

Investigating the exact mechanism of action of the newly identified players and providing possible links between these and the rest of the epidermal gene network is certainly of interest. It is of note that TaDa will be a key method towards achieving this objective, as it can be used to identify transcription and chromatin factor targets in the tissue of interest.

We looked into what the reviewer suggested and indeed found a conserved binding site for *mir-44* in the *efl-3* 3' UTR. We investigated potential *efl-3* regulation in the lines overexpressing *mir-44* by smFISH, but found no significant difference in *efl-3* expression in seam cells. We have not included these data in the manuscript, as we prefer to do such experiments more systematically as part of our future work once we understand the developmental basis of the seam cell phenotypes observed in greater detail and repeat this experiment with stable mutants.

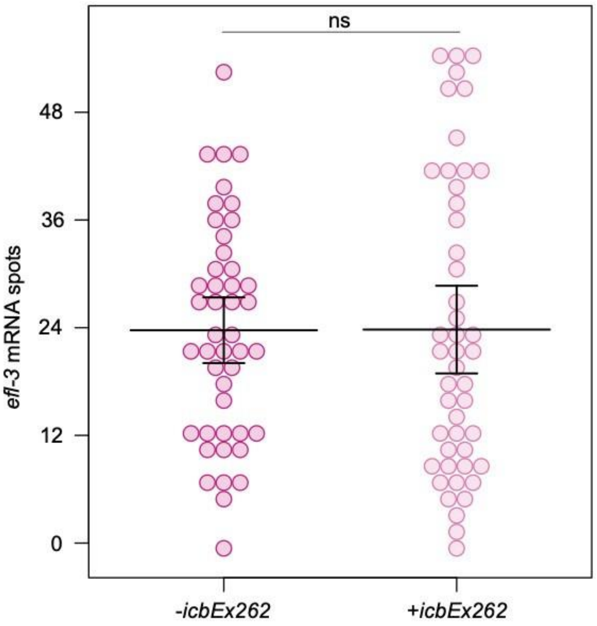

At this stage, I will not argue for testing the six genes mentioned above using bona fide mutations. However, the authors should highlight in the discussion that partial RNAi effect might explain why some of the other genes tested by RNAi seem not to be required for seam cell division or differentiation, and furthermore that a stronger/null allele of the six aforementioned genes might have generated more penetrant effects.

We added a comment to clarify this in the discussion. In some cases (e.g. *efl-3* or *hda-1*) these genes are also essential so our plan is to attempt tissue-specific knock-outs by making use of Cre-based recombination systems.

Other: please give the meaning of the abbreviations TSS and TES when it first appears (it is currently shown only late in the Methods section).

We have now provided the definition in the text in the first instance where the abbreviation appears.

Reviewer 2, Advance Summary and Potential Significance to Field:

Knowledge of tissue- and cell type-specific gene expression patterns is important for our understanding of the development and function of multicellular organisms. In C. elegans, this has

© 2021. Published by The Company of Biologists under the terms of the Creative Commons Attribution License (https://creativecommons.org/licenses/by/4.0/).

been difficult to obtain, since classical dissection tools do not work well, and other approaches such as PAT-seq, INTACT or scRNA seq are either not very reliable or expensive. Katsanos et al. report the adaptation of an alternative approach, Targeted- DamID/TaDa to C. elegans, where they use it to analyze the transcriptomes of two distinct epidermal compartments, the stem cell-like seam cells and the postmitotic hypodermal cells. They test some genes that they report to be seam cell-specific for a function in determining seam cell number, as a proxy for a function in "stemness". The approach reported is interesting and potentially powerful. At this point, however, we feel that the data presented do not provide sufficient validation that the method works as advertised, and the biological insight appears limited as the evidence for a stem cell function of the tested genes is weak.

We thank the reviewer for their comments and feedback. We have followed the suggestions for all additional data analysis to strengthen our manuscript. We have toned down statements about stemness to clarify to the reader that the aim of this study is to establish TaDa for gene expression analysis and produce comprehensive datasets (in combination with data generated via other methods) to allow in the future more mechanistic studies on epidermal cell types.

Reviewer 2, Comments for the Author:
Major points:
1. We have a hard time understanding how two quite different biological samples, i.e., L2- stage seam and L4-stage hyp can correlate at r=0.99 (Fig. S3). This is a level of correlation usually difficult to obtain for RNA-seq among biological replicate experiments. It seems even more unexpected given that gene expression patterns in these particular tissues are known to be highly dynamic, with e.g. widespread oscillatory expression of collagen genes (e.g., Hendriks et al., Mol Cell 2014). Hence, even the question of distinct cell-types aside, it is difficult to understand how the samples from different stages can be so similar. One possibility is that TaDa yields a very "static" signature, i.e., once methylation is present, it will stay on for a long time. This clearly affects then the interpretation of TaDa data in general, because it might yield (for instance) a false impression of similarity between the transcriptomes from tissues derived from the same progenitor. We suggest that the authors take a handful of genes that are on early in development but off at a later time to compare their signatures.

The correlations in DamID experiments are not directly comparable to transcriptome correlations. The key difference is that DamID correlations rely on the scores of all the~269000 GATC fragments across the whole genome, therefore they include GATC fragments in intergenic areas (~70000 GATC fragments) that are expected to be mostly devoid of signal in the case of genuine RPB-6 binding. While this is informative for assessing the overall binding characteristics of the TaDa fusions, this positional preference leads to the high correlation values observed, and is similar to what is reported for other DamID datasets (e.g. Cheetham et al., Development 2018, Senet al., eLife 2019, Hassan et al., eLife 2020, van de Walle., PLoS One 2020, Saldivar et al., Genetics 2020). In addition to the above, since RPB-6 participates in all 3 RNA polymerase complexes, ubiquitous methylation patterns from RNApol I and RNApol III activity (Saldivar et al., 2020) may also somewhat increase the correlation values.

We emphasise in the discussion that all methods have their strengths and weaknesses. When it comes to capturing dynamics of expression like gene expression oscillations, DamID is generally not well-suited to this task as methylation is likely to be maintained at least until the next cell division. We now mention this limitation in the discussion. This is not likely to be a problem in the seam cells, which divide during post-embryonic development possibly leading to methylation signal "reset". Although we cannot rule out some methylation carry-over as in all DamID experiments, the fact that we see many distinct peaks at L4 compared to L2 is encouraging.

Regarding the point about artificial similarity between tissue-profiles based on progenitor transcriptomes, we avoided methylation of genes in progenitor cells by identifying new enhancers that drive expression specifically in the post-embryonic seam cells and exclusively in the post-mitotic hypodermis. With this experimental design, we exclude expression of the Dam fusions within a common cell progenitor, so we do not anticipate technical similarities due to common lineage.

© 2021. Published by The Company of Biologists under the terms of the Creative Commons Attribution License (https://creativecommons.org/licenses/by/4.0/).

2. Even allowing for the possibility of a "static" TaDA signal, we fail to reconcile the high levels of similarities with the fact that the authors report that hundreds of genes can be detected as specifically expressed in only one vs the other sample (e.g., in the comparison from above, >900 genes, according to Fig. S5 and assuming we did not miscount); overall between the two tissues 1090 genes (ll.329-330) – of fewer than 3,000 detected in each sample (ll.242-245). To us, this suggests that correlations are dominated by systematic noise, not by signal. We would like to see scatter plots with pairwise comparisons (between replicates, between stages, between tissues, respectively), specifically for "genic" signal to have some assurance that there is indeed some level of reproducibility in signal, and (for the comparison between tissues or stages) with an indication of which genes (dots in the plot) are called significantly different based on the tests the authors apply.

As outlined in the response to the previous comment, DamID correlations are not correlations between transcriptomes but compare distributions of genome-wide signal per GATC fragment across samples. Genes specifically found in certain stages and cell-types are only identified after gene-calling using the genome-wide signals. This analysis relies on very small proportion of the total genome-wide GATCs that are used for the calculation of correlations (we estimated this to be between 1.3% - 2.5% of all GATC fragments in our samples), therefore these GATC fragments contribute much less to the overall level of similarity.

To make this distinction clearer and address the reviewer's comment, we have performed the correlation analysis across replicates, cell types and stages restricting to only those GATC fragments that participate in protein coding genes. As expected, we find that the correlation values are somewhat deflated once the intergenic regions are removed. These data are presented in the form of scatterplots in the revised Figure S3.

We stress that these data simply reflect reproducibility of methylation patterns albeit in a more restricted proportion of the genome, and therefore point to similarity in how genic regions are occupied by RPB-6. These data are not equivalent to gene expression correlations e.g. via RNA-seq. The equivalent of such correlation analysis for TaDa would have to be performed after gene-calling, which converts the signal profiles to gene expression data with an assigned FDR and a TaDa expression value. We performed the new analysis requested by the reviewer in the form of scatterplots now presented in the revised Figure 4. These results still show good correlation across stages and cell-types ruling out that correlations are driven by noise in the data.

3. Related to the two points above, based on gels shown in Figure S2B, the authors conclude that the cell-type-specific rpb-6::Dam-fusions for TaDa produce robust methylation (ll. 170-172). It is difficult for us to observe any clear, reproducible patterns, the two replicates seem to differ greatly (for example sfr-3i1::dam::rpb-6 in L2 stage shows no bands or smear in r1 and low intensity bands in r2; same for different intensity of bands, e.g., dpy-7syn1::dam::rpb-6 in L4 stage show high intensity bands in r1 and low intensity bands in r2). Indeed, are the authors not concerned by the strong and discrete bands they observe in several samples? It is our understanding that methylation on thousands of genes should yield a smear instead. Could the authors please comment and explain how they conclude robustness from this experiment.

The gels in Figure S2B of the previous version of our manuscript were loaded with the same volume of PCR product (rather than the same DNA concentration) therefore the discrepancies in intensity of bands or smears were likely down to the varying DNA amounts in each sample. Such bands may represent highly methylated regions of the genome, for example highly expressed genes, but do not interfere with the downstream analysis. Our comment about robustness was mostly empirical – we have observed qualitatively the same banding pattern over multiple experiments for various different fusions we have profiled, so these patterns appear to be fusion-specific and presumably non-random.

To look into this further, we investigated coverage files based on raw mapped read data to find regions of the genome with substantially larger read counts than the average. We did find cases that may correspond to these visible bands. For example, we discovered that a~4 kb region on the telomere of chromosome I (~15064000-15068000) overlapping ribosomal RNA genes (*rrm-2.1* and *rrm-3.1*) had an average coverage ranging between 3419x and 5763x that of the genome-wide

© 2021. Published by The Company of Biologists under the terms of the Creative Commons Attribution License (https://creativecommons.org/licenses/by/4.0/).

average at L2. Since RPB-6 participates in the RNApol I complex, this very high signal enrichment is possible to be biologically relevant. Since we cannot formally prove that these areas with high coverage fully correspond to the bands we observed on the gel, we have removed Fig. S2B from the revised manuscript to avoid any confusion.

4. It is somewhat unclear to us what the dam:NLS-GFP control really controls for. The authors suggest that it causes stochastic methylation (l.185-187), but it seems normalization for systematic errors (e.g., DNA regions that get preferentially methylated, or erroneously called as methylated, even without an rbp-fusion) is more important. Yet, given that the gels in Fig. S2 appear mostly devoid of any signal (smear or discrete bands) for this control, we do wonder whether this control can see more than random noise, highly amplified.

We have changed the text to clarify that control fusions capture background levels of methylation, for example occurring in accessible regions of the genome. Since the control fusions are subject to the same technical biases as the RPB-6 fusions, they allow to compare the RNApol occupancy profiles against background signal. In general, we do see a smear in control samples too, as expected. Again, lack of smear in some wells in Figure S2B in the previous version of the manuscript was due to varying amounts of DNA loading on the gel.

5. The authors suggest that the high level of overlap in seam cell vs. hypodermis TaDa signal could reflect a common epidermal nature (ll.326-328). The above concerns about a “static” signal aside, it is unclear why detection of a TaDa signal would signify tissue- specific expression in the first place: conceptually, we would expect to see a signal as long as the gene of interest is expressed in a given cell, irrespective of whether expression occurs in only this tissue (i.e., is specific), or occurs in other, perhaps even all, tissues as well. Hence, by default, “specificity” can only be demonstrated by intersecting data from different tissues. A statement on similarity would require examination of at least one other tissue, ideally of developmentally “remote” origin.

Intersections of gene sets is a commonly used method to assess the similarity of newly acquired gene expression data to already available datasets. We agree with the reviewer and we did not mean to infer that the identified overlaps necessarily correspond to genes expressed tissue specifically. The default hypothesis is that these overlaps should contain a mix of ubiquitously expressed and epidermal genes. To confirm this hypothesis, we used the gene set analysis tool from Serizay et al., Genome Research 2020 to compare our dataset to tissue-classified gene sets. We do find that our gene sets contain multiple ubiquitously expressed genes and substantial overlap with the hypodermis (there is no seam cell dataset in this reference). Acquiring TaDa profiles for other tissues is certainly of interest as part of our future work. We have included these new data in revised Figure S6.

6. Although the authors claim that TaDa data agree overall well with other approaches aimed at obtaining tissue-specific gene expression data, we do not find these data convincing: In Figure S5A, B, the intersections between TaDa and either PAT-seq or sciRNA-seq are shown, with large numbers of intersecting genes apparently meant to show reproducible detection of tissue-specific expression. However, related to the previous point, in the way this is done, it is unclear whether the intersection represent overlap in tissue-specific or merely in broadly/ubiquitously expressed genes. We would argue that the analysis in S5C, if extended as described in the next point, would better serve the purpose. However, if the authors still wish to keep the type of analysis shown in S5A,B, it would be important that they show the overlap among genes called as tissue-specifically expressed in seam and hyp respectively in either data set (i.e., for Cao et al., use the criteria described in that paper, namely TPM cut-off plus ≥5-fold higher level in the highest than the second most highly expressing tissue). As a negative control, this overlap could then also be documented for tissues other than seam and hypodermis, e.g., VPC, rectum, intestine, neurons.

We agree with the reviewer that it would be valuable to know how the genes that we identify here as expressed only in the seam cells or only in the hypodermis compare to tissue- specific gene sets determined in Cao et al 2017. To this end, we performed additional intersections with gene sets specific for the seam cells, the hypodermis and other non- related cell-types from that study and present these in the revised Figure S7. Reassuringly, the largest overlap and most significant

© 2021. Published by The Company of Biologists under the terms of the Creative Commons Attribution License (https://creativecommons.org/licenses/by/4.0/).

enrichment is found in intersections with gene sets from the same cell type between TaDa and Cao et al. (seam cell-specific or hypodermis- specific respectively).

7. The above analysis, even if done properly, will only give a qualitative view of overlap, and suffers from an arbitrary choice of cut-offs. A more relevant analysis is provided in Figure S5C, where a correlation between quantitative signals between TaDa and the other approaches is tested. The authors conclude (ll. 314 – 316) that this reveals significant correlation. While this appears to be technically correct based on the statistical test used, it is also rather misleading, because the authors present a p-value for presence of any correlation rather than an r-value to quantify the extent of correlation. Looking at S5C, the extent of correlation to the current gold standard, sci-RNA-seq, appears minimal. Even for the correlation that is observed, it is unclear whether this reflects a signal of tissue-specificity, for which “cross”-correlations (e.g., seam TaDa vs. hyp sci-RNA-seq and its converse) would be needed.

The reviewer is right to point out that despite the significant correlation, there is only weak positive association. We have included the r values in the updated figure so that they are available to the reader. However, it is important to stress that this was more of an exploratory analysis to assess whether TaDa signals can capture any useful quantitative information other than pinpointing genes expressed in a given tissue. Transcription is captured in TaDa as methylation, the level of which is likely to be influenced by many factors beyond the frequency of transcription. It is not therefore given that TaDa results can be treated in a fully quantitative manner. On this basis, the fact that we find TaDa expression values to correlate to some extent to those of a *bona fide* quantitative method like sci-RNA-seq is noteworthy, especially considering that seam cell transcriptomes from the quantitative sci-RNA-seq and PAT-seq methods fail the same statistical test for correlation.

To strengthen the potential quantitative aspect further, we investigated the relationship of ranked TaDa-identified expression values to sci-RNA-seq expression. The results show a substantial overlap that further supports the similarity to sci-RNA-seq findings. This new analysis is now included as Figure 4B.

With this analysis we did not attempt to make any claims about tissue-specificity as some of the genes used to calculate the correlations are common across cell-types. Therefore, cross-tissue correlations presented below also show the same pattern (significant correlation with weak positive association).

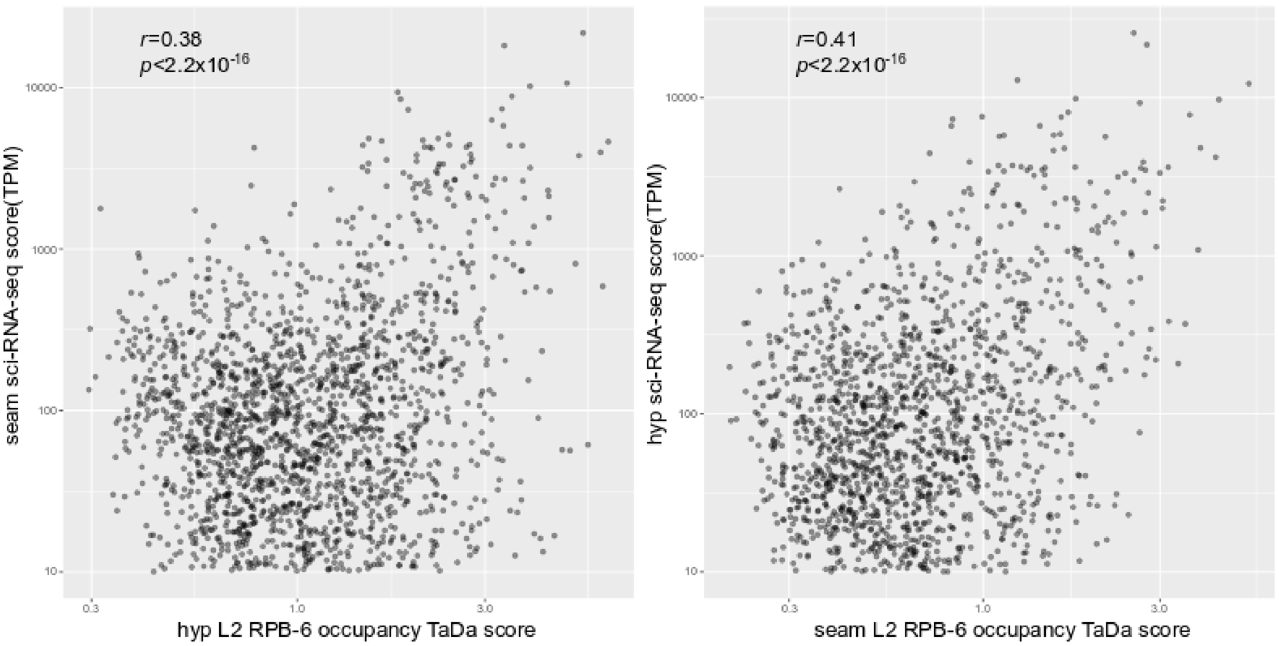

© 2021. Published by The Company of Biologists under the terms of the Creative Commons Attribution License (https://creativecommons.org/licenses/by/4.0/).

8. More generally, the number of genes with TaDa signal in any condition appears quite low (~3,000); the authors need to assess whether this set is biased in any particular way (gene length, expression levels based on sci-RNA seq but also whole worm seq, etc.)

The number of genes identified are indeed lower than those identified by sci-RNA-seq (although not significantly different to those identified for example for the seam cells by PAT-seq). We do discuss in the manuscript potential TaDa biases that could be driving the difference to sci-RNA-seq, mostly pertaining to GATC availability and low expression levels.

Following the reviewer's suggestion, we have separated all protein coding genes according to whether they are expressed in the cell type of interest or not and generated density plots for the number of GATCs that make up a gene, gene length and expression levels based on cell type-matched sci-RNA-seq and stage-matched whole-animal RNA-seq. We find that genes identified by TaDa as expressed for all cell-types and stages show also higher expression values in RNA-seq. We also find that TaDa expressed genes are biased towards higher GATC numbers and gene length, which is not very surprising due to the reliance of this method on GATC sequences. Interestingly, the same bias was revealed the in sci-RNA- seq data for higher expression when gene length increases. These data have been added to the manuscript as Figure S5.

9. To test whether transcription or chromatin factors, or miRNAs, identified as tissue- specifically (seam or hyp) expressed function in seam cell development, the authors deplete some by RNAi or over-express them and count whether any changes in seam cell numbers (mean or variance) occur. This seems a rather crude assay, and it certainly falls short of delivering on what from the introduction appears to be the main motivation for establishing TaDa in seam and hyp cells, showing that any of these factors affect stem cell fates. (For instance, the migration of seam cell daughters or fusion with hyp7 could alternatively be affected). If the authors feel that a more extensive characterization of at least one of these factors is currently out of their reach, they should tone down this point in their introduction and acknowledge the limitation of their seam cell-counting assay explicitly in the discussion.

The reviewer is right to point out that seam cell number quantification is a crude yet useful read-out, given the consistency of developmental patterning in *C. elegans*. We have added a sentence in the discussion to explicitly say that the seam cell counting assay lacks resolution to dissect the developmental basis of the observed phenotypes. Understanding the exact mechanism of action of the TaDa-identified E2F (*efl-3*) or chromatin factor (*hda-1*) will be the focus of our future work.

Minor points
1. Although mature let-7 accumulates indeed mostly in L4, the let-7 gene is transcribed at all stages (e.g., Perales et al., PLoS Genet 2014, van Wynsberghe et al., Nat Struct Mol Biol 2011) and regulation is post-transcriptional, hence, the data in Fig. 6B would appear to argue against rather than in favor of the specificity of TaDa.

We thank the reviewer for pointing this out. This might reflect the oscillations reported for *let-7* expression in all developmental stages. Since this example is not central to our story, we have removed it from the figure.

2. While the authors state "Moreover, the miRNA cluster mir-42, mir-43 and mir-44 was found to be expressed only in the seam cells in both stages while mir-47, was found to be expressed only in the hypodermis in both stages (Figure 6B), which matches previous reports based on transcriptional reporters (Martinez et al., 2008).", this is what the reference states: " Pmir-42-44: dorsal and ventral part of the embryo, including head. Late embryos show complex expression. Strong expression in seam cells and vulva. Weaker expression in hypodermis. Also seeing in posterior intestine and rectum and dnc." (our emphasis) and "Pmir-47: Expression detected from late embryos to adults. In embryos, expression is detected on lateral sides and in larval stages on, in all hypodermis, rectum and vulval cells." (where, admittedly, the phrasing is not very clear but appears to suggest expression in both seam and other hypodermal compartments).

We have corrected the text to be more precise. Previous reports supported epidermal expression in general but did not focus on epidermal cell-type-specific expression.

© 2021. Published by The Company of Biologists under the terms of the Creative Commons Attribution License (https://creativecommons.org/licenses/by/4.0/).

3. In Figure S1D and S1E, the developmental stages of the imaged worms need to be indicated
OK, added.

4. In Figure 2A-B, the ordering of the samples in the Pearson correlation heatmap is rather confusing to us; organization of samples from left to right and top to bottom (so that the diagonal of autocorrelation would go from top left to bottom right) would appear more conventional.

This is the default of a commonly used tool for correlation analysis in deepTools so we prefer to leave as it is.

5. Please label the y-axis In Figure 2D.

OK, added.

6. In ll. 393-395 the authors state that they overexpress microRNAs in the epidermis. Please explain in the main text how overexpression was achieved.

OK

7. In l. 179, the authors write that they used materials for sequencing from ~2000 individuals, instead in l. 430 they write “unique reads were acquired starting from a moderately-sized population of ~5000 individuals”. Please indicate the right number of individuals used.

Corrected (from as little as 2000 is the right number).

Other:
1. The tissue-specific expression of constructs is difficult to appreciate from the images in Figure S2A.
Images at higher magnification would help; but for the future, the authors might do well to use mCherry::H2B or similar to concentrate signal in the nuclei of expressing cells, which will yield a better signal-to-noise ratio for expressing cells. In the specific case of seam vs hyp distinction, we also note that hypodermal nuclei derive from seam cells through asymmetric division. Hence, to demonstrate specificity, it seems preferable to score reporter expression immediately after one of the asymmetric divisions (ideally L2 or early L4), and not in late L4 as currently done. (We do note that since the stability of the reporter may differ from that of the Dam fusion protein, even this piece of evidence may be subject to challenge, but it seems to be the best evidence currently attainable.)

The specificity of the promoters is shown in Figure S1. We did use L2-L4 asymmetric division to assess the expression driven by the *dpy-7* promoter to exclude expression in seam cells. We have considered adding an H2B tag to facilitate imaging, but as discussed in the introduction the exact length of the primary ORF is important for TaDa to produce the right amount of ribosomal reinitiation. Therefore, adding this tag would require establishing first its influence on the levels of secondary ORF expression.

<u>Second decision letter</u>

MS ID#: DEVELOP/2021/199452

MS TITLE: Gene expression profiling of epidermal cell types in C. elegans using Targeted-DamID

AUTHORS: Dimitris Katsanos, Mar Ferrando-Marco, Iqrah Razzaq, Gabriel Aughey, Tony D Southall, and Michalis Barkoulas

© 2021. Published by The Company of Biologists under the terms of the Creative Commons Attribution License (https://creativecommons.org/licenses/by/4.0/).

I have now received all the referees' reports on the above manuscript, and have reached a decision. As you will see from the reviewer comments, the reviewers disagree on some key aspects of the study. Mainly, as Reviewer 2 states, some of the key aspects of the data are currently in the supplement, and this could be because they show low correlation. However, for the tool to be used by the field, it is essential for the field to be aware of all of its strengths as well as weaknesses. Having consulted with additional members of the editorial board on this aspect, and given Development's commitment to rigor in a study, I ask that you address reviewer 2's point on data reorganization and presentation, including a clear discussion of the strengths and weaknesses of the tool presented, and why it may be complementary to, if not better than, PAT-Seq.If you are able to revise the manuscript along the lines suggested by Reviewer 2 I will be happy receive a revised version of the manuscript.

Please attend to all of the reviewers' comments and ensure that you clearly highlight all changes made in the revised manuscript. Please avoid using 'Tracked changes' in Word files as these are lost in PDF conversion. I should be grateful if you would also provide a point-by-point response detailing how you have dealt with the points raised by the reviewers in the 'Response to Reviewers' box. If you do not agree with any of their criticisms or suggestions please explain clearly why this is so.

Reviewer 1

Advance summary and potential significance to field

This work introduces the method TaDa in C. elegans, which is based on a genome-wide genomic analysis of the RBP-6 RNA Pol subunit under the expression of tissue-specific promoters. This method enabled the authors to identify genes known to be expressed in the larval epidermis and seam cells, as well as novel genes that had not previously been linked to the epidermis/seam.

Comments for the author

I am satisfied with the revisions and clarifications brought by the authors and confirm my initial support for this manuscript. Their discussion does a nice job at comparing the advantages of the genome-wide methods available to identify genes acting in a given tissue, and I remain convinced that this TaDa approach is of great interest.

One note, which I leave to the decision of the Editor, there are nearly 100 references and the authors may want to consider reducing this number.

Reviewer 2

Advance summary and potential significance to field

Knowledge of tissue- and cell type-specific gene expression patterns is important for our understanding of the development and function of multicellular organisms. In C. elegans, this has been difficult to obtain, since classical dissection tools do not work well, and other approaches such as PAT-seq, INTACT or scRNA seq are either not very reliable or expensive. Katsanos et al. report the adaptation of an alternative approach, Targeted-DamID/TaDa to C. elegans, where they use it to analyze the transcriptomes of two distinct epidermal compartments, the stem cell-like seam cells and the postmitotic hypodermal cells. They test some genes that they report to be seam cell-specific for a function in determining seam cell number, as a proxy for a function in “stemness”.

Comments for the author

The authors have addressed the points that we raised previously through clarifications and additional analysis. Importantly, and as requested, they now analyzed the reproducibility of TaDa in calling protein gene expression from biological replicates. The results are somewhat sobering, with a correlation of as low as 0.67 for the two L2-stage seam cell samples. There may be reasons for this, and other samples look much better but this is not discussed. Indeed, what concerns me most about the current manuscript is that the authors appear to make every effort to hide the

© 2021. Published by The Company of Biologists under the terms of the Creative Commons Attribution License (https://creativecommons.org/licenses/by/4.0/).

shortcomings of the method rather than being candid about them undermining their own credibility. Thus, although they promote TaDa as a gene expression profiling tool, they hide the above data in a supplemental figure and merely mention that correlations are “somewhat weaker” than what is shown in the main figure, looking genome-wide (where correlations are 0.98 and higher, but, as stated in the rebuttal letter, correlation is mainly driven by intergenic fragments devoid of signal). While low correlation may not completely invalidate the approach (though I would not want to invest my lab’s time or money into trying it out), at least readers should know what to expect. For this, I suggest that the current Fig. 2 becomes a supplemental figure and that a new Fig. 2 contain a) a summary heatmap as in the current 2A and B, but now for GATCs at protein-coding genes only and b) the scatter plots currently shown in Fig. S3C.

Concerning their discussion of tissue-specific small RNA profiling, the authors also may wish to mention MIME-seq, Alberti et al. Nature Methods 2018.

Second revision

Author response to reviewers' comments

Reviewer 1 comments:
I am satisfied with the revisions and clarifications brought by the authors and confirm my initial support for this manuscript. Their discussion does a nice job at comparing the advantages of the genome-wide methods available to identify genes acting in a given tissue, and I remain convinced that this TaDa approach is of great interest. One note, which I leave to the decision of the Editor, there are nearly 100 references and the authors may want to consider reducing this number.
We have kept the same number of references as per the guidelines of the journal.

Reviewer 2 comments:

The authors have addressed the points that we raised previously through clarifications and additional analysis. Importantly, and as requested, they now analyzed the reproducibility of TaDa in calling protein gene expression from biological replicates. The results are somewhat sobering, with a correlation of as low as 0.67 for the two L2-stage seam cell samples. There may be reasons for this, and other samples look much better, but this is not discussed.
We thank the reviewer for reading our manuscript once again and providing further suggestions. The correlation values in Fig. S3C are likely to be sensitive to the exact localisation of the signal on specific GATC fragments within protein-coding genes. For example, genuinely expressed genes may show quantitative differences in the methylation signal from one GATC bin to another or differences in how much the signal spreads. Such differences in signal distribution across GATC fragments may influence correlation levels although they may not convey useful biological information when it comes to defining the expression of genes. To address this hypothesis, we produced new Pearson correlation heatmaps for GATCs within protein-coding genes, but this time using the average signal across protein-coding genes. Interestingly, the correlation values on the gene level are even stronger than before, with a minimum value of 0.84 (see revised Fig. 2B). Therefore, signal on a per protein-coding gene basis is reproducible across replicates.

Indeed, what concerns me most about the current manuscript is that the authors appear to make every effort to hide the shortcomings of the method rather than being candid about them, undermining their own credibility. Thus, although they promote TaDa as a gene expression profiling tool, they hide the above data in a supplemental figure and merely mention that correlations are “somewhat weaker” than what is shown in the main figure, looking genome-wide (where correlations are 0.98 and higher, but, as stated in the rebuttal letter, correlation is mainly driven by intergenic fragments devoid of signal). While low correlation may not completely invalidate the approach (though I would not want to invest my lab’s time or money into trying it out), at least readers should know what to expect.
We attempted to integrate many interesting findings based on the initial suggestions by the reviewer, so inevitably some parts of the analysis had to be included in the supplement. We would

© 2021. Published by The Company of Biologists under the terms of the Creative Commons Attribution License (https://creativecommons.org/licenses/by/4.0/).

like to stress that correlations using all GATC fragments of the genome are not trivial and they are necessary to evaluate the overall binding characteristics of the *dam:rpb-6* fusions, which have the capacity to cause genome-wide methylation. We have reorganised the discussion to stress further the strengths and weakness of TaDa in comparison to other methods previously used for the same purpose.

For this, I suggest that the current Fig. 2 becomes a supplemental figure and that a new Fig. 2 contain a) a summary heatmap as in the current 2A and B, but now for GATCs at protein- coding genes only and b) the scatter plots currently shown in Fig. S3C. We understand that the high correlations reported when all GATC sites are considered can be confusing. Based on the reviewer's suggestion, we have moved these heatmaps to the supplement and use the PCA analysis to demonstrate the distinct clustering between control and *dam:rpb-6* fusions. We also include our new heatmap showing Pearson correlations for all samples based on average signal per protein-coding gene. We finally report in the main text the exact correlation values in the analysis of signal per GATC bin of protein-coding genes and average signal per gene to ensure the reader has easy access to this information. This way we feel the presentation of results is succinct and at the same time we have managed to keep the signal tracks and signal distribution around genes that some readers may also want to see for this newly established technique.

Concerning their discussion of tissue-specific small RNA profiling, the authors also may wish to mention MIME-seq, Alberti et al. Nature Methods 2018.
Added, thank you.

<u>Third decision letter</u>

MS ID#: DEVELOP/2021/199452

MS TITLE: Gene expression profiling of epidermal cell types in C. elegans using Targeted-DamID

AUTHORS: Dimitris Katsanos, Mar Ferrando-Marco, Iqrah Razzaq, Gabriel Aughey, Tony D Southall, and Michalis Barkoulas
ARTICLE TYPE: Research Article

I am happy to tell you that your manuscript has been accepted for publication in Development, pending our standard ethics checks.

© 2021. Published by The Company of Biologists under the terms of the Creative Commons Attribution License (https://creativecommons.org/licenses/by/4.0/).
