# Supplementary material for: Gene expression profiling of epidermal cell types in C. elegans using Targeted-DamID
Source: Development. Author manuscript; Available in PMC 2022 Aug 8. (PMC7613258; doi:10.1242/dev.199452)
Supplement: Supplementary file 1 [file EMS151619-supplement-Supplementary_information.pdf]

Gene expression profiling of epidermal cell types in *C. elegans*

using Targeted-DamID

Dimitris Katsanos[1], Mar Ferrando-Marco[1], Iqrah Razzaq[1], Gabriel Aughey[1], Tony Southall[1] and Michalis Barkoulas[1]★

[1]Department of Life Sciences, Imperial College, London SW7 2AZ, United Kingdom.

★Corresponding Author: m.barkoulas@imperial.ac.uk

Supplemental Figures S1-S8

Supplemental Tables S1-S6

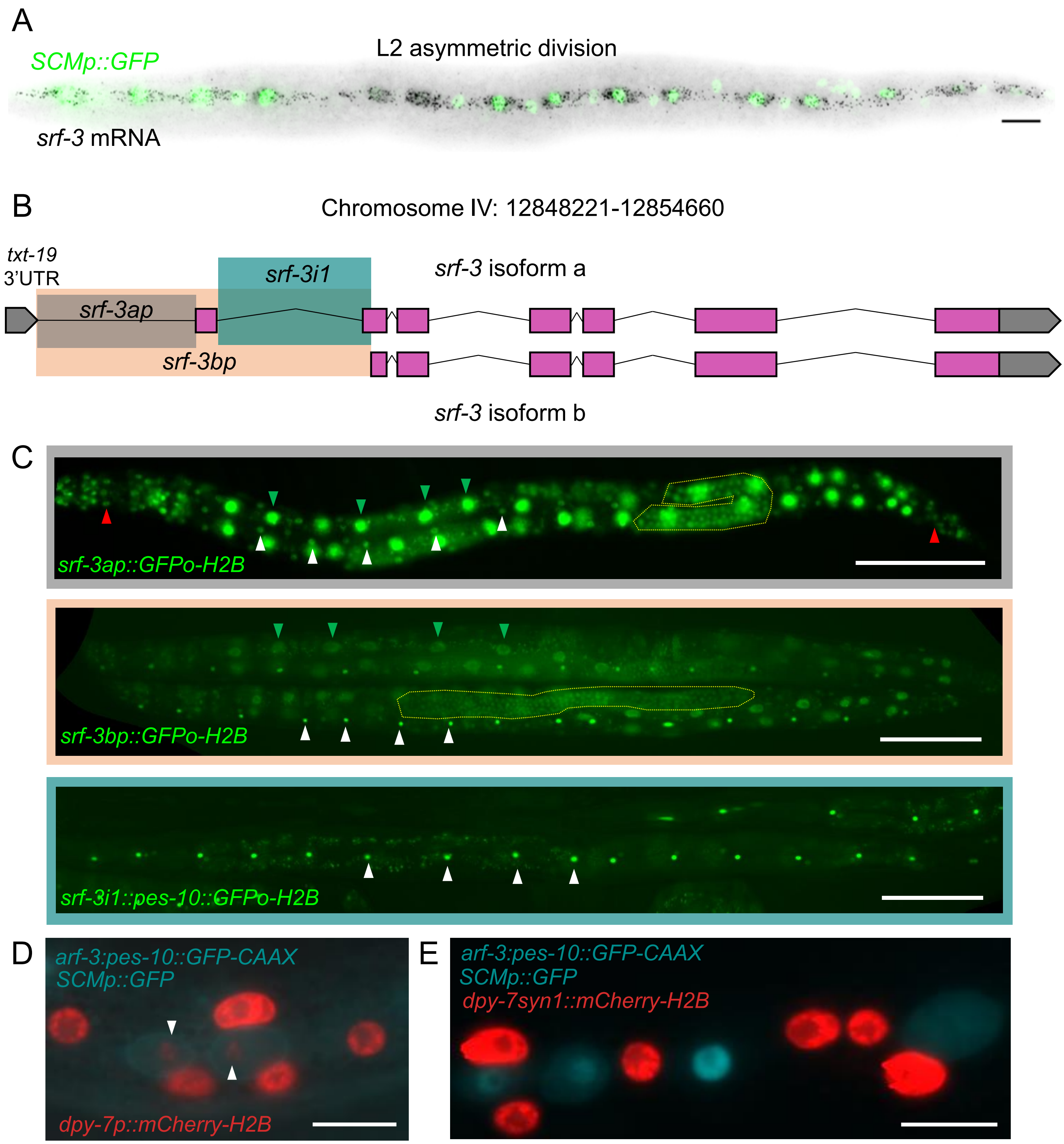
A
SCMp::GFP
L2 asymmetric division
srf-3 mRNA
B
Chromosome IV: 12848221-12854660
txt-19
3'UTR
srf-3i1
srf-3 isoform a
srf-3ap
srf-3bp
srf-3 isoform b
C
srf-3ap::GFPo-H2B
srf-3bp::GFPo-H2B
srf-3i1::pes-10::GFPo-H2B
D
arf-3:pes-10::GFP-CAAX
SCMp::GFP
dpy-7p::mCherry-H2B
E
arf-3:pes-10::GFP-CAAX
SCMp::GFP
dpy-7syn1::mCherry-H2B

Figure S1

Figure S1: Establishing seam cell and hypodermis-specific promoters for RNApol TaDa.

(A) Representative image of *srf-3* smFISH at the L2 stage, during the asymmetric seam cell division, showing high levels of transcript in seam cells. Seam cell nuclei are marked by the *SCMp:GFP* reporter and *srf-3* mRNAs appear as black spots. **(B)** Illustration of the *srf-3* locus on chromosome IV. Pink blocks signify exon sequences and grey indicate 3' UTRs. Two isoforms of *srf-3* (isoform a and b) are shown. Shaded areas near the 5' mark putative promoter or regulatory sequences tested for seam cell specific expression. With grey, the 1093 bp putative promoter sequence of isoform a (*srf-3ap*), extending from the end of the upstream gene *txt-19* 3'UTR to the *srf-3* isoform a start codon. With peach, the 2246 bp sequence starting at the same position and extending to the start codon of isoform b (*srf-3bp*) and with teal, the 1081 bp first intron of isoform a (*srf-3i1*). **(C)** Representative fluorescence images of late L4 transgenic animals carrying single-copy transgenes of transcriptional reporters driving expression of *GFP-H2B* under the *srf-3ap* promoter (grey frame), the *srf-3bp* promoter (peach frame) and the *srf-3i1::pes-10* promoter (teal frame). White arrowheads show expression in seam cell nuclei, green in intestinal and red in hypodermal. Yellow outlined areas indicate further expression in the germline. **(D-E)** Representative fluorescence images showing expression of *mCherry-H2B* under the promoter of *dpy-7* from a single-copy transgene (D) and *dpy-7syn1::mCherry-H2B* from a multi-copy transgene (E) at the L3 asymmetric cell division stage. Seam cells are marked in cyan by membrane (*arf-3:pes10::GFP-CAAX*) and nuclear (*SCMp::GFP*) reporters. Note expression of *dpy-7p:mCherry-H2B* in seam cell nuclei, indicated by white arrowheads, which is more prominent during divisions and is abolished in *dpy-7syn1::mCherry-H2B* transgenics. Scale bars are 100 μm in C and 10 μm in D and E.

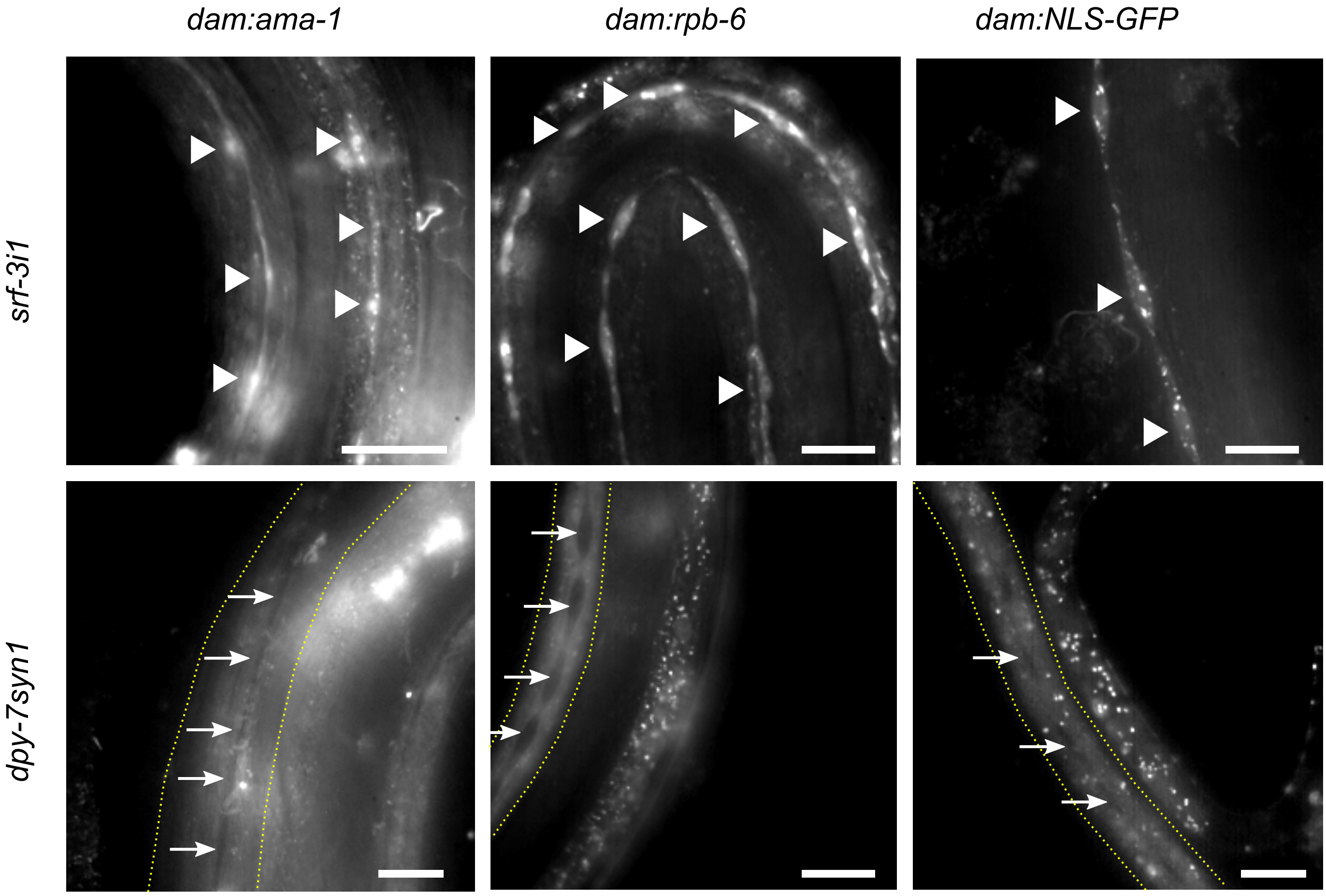

Figure S2

Figure S2: Validation of expression of the RNApol TaDa transgenes.

Confirmation of single-copy transgene expression in the expected tissue of interest, for specified transgenes, using *mCherry* expression as a proxy. Animals were imaged at the L4 stage. White arrowheads indicate expression in the seam cells, while white arrows indicate absence of expression in the seam cells in animals expressing in the hypodermis, indicated by the yellow outline. Scale bars are 20 μm.

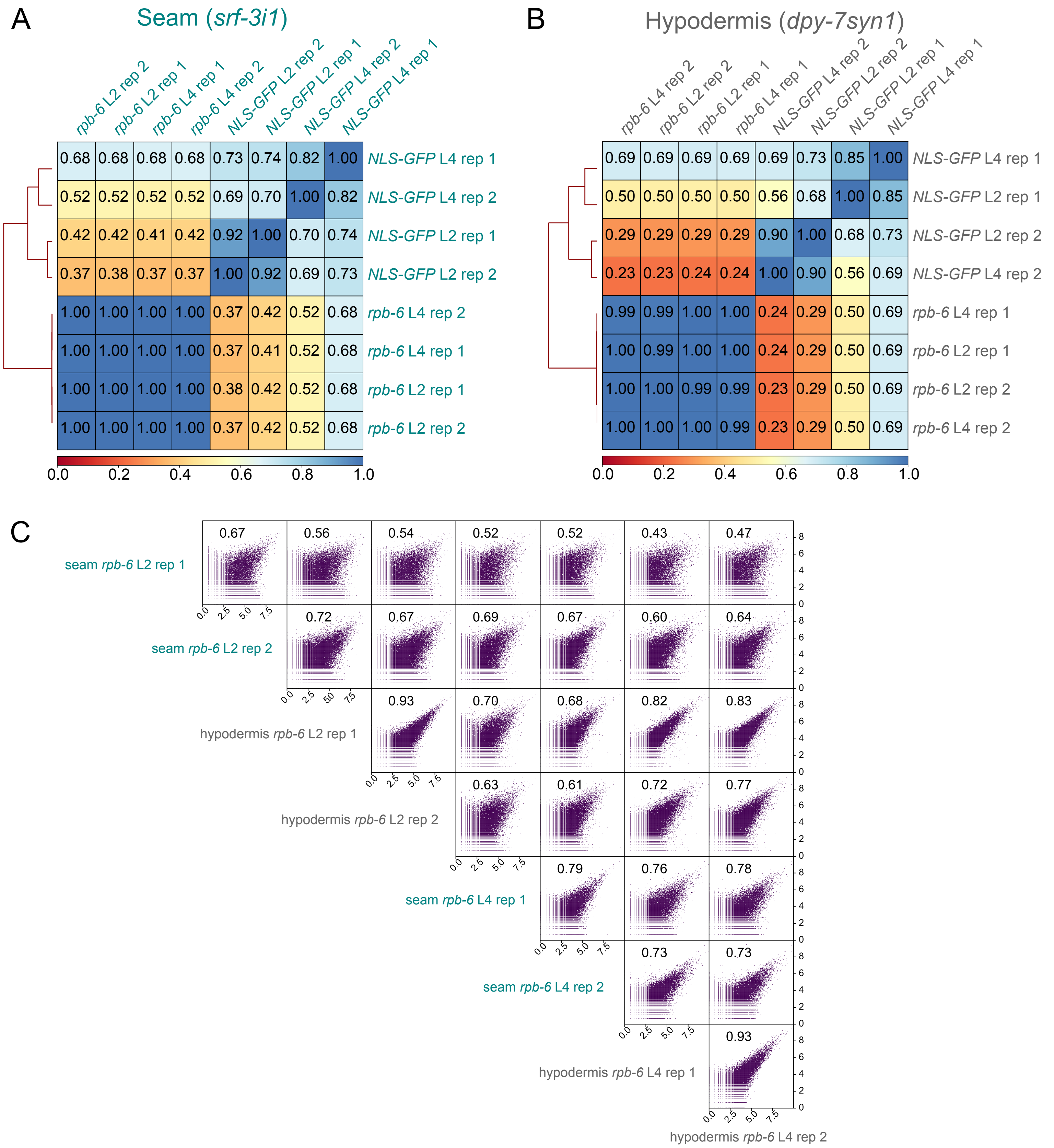
A
Seam (srf-3i1)
B
Hypodermis (dpy-7syn1)
C
seam rpb-6 L2 rep 1
seam rpb-6 L2 rep 2
hypodermis rpb-6 L2 rep 1
hypodermis rpb-6 L2 rep 2
seam rpb-6 L4 rep 1
seam rpb-6 L4 rep 2
hypodermis rpb-6 L4 rep 1
hypodermis rpb-6 L4 rep 2

Figure S3

Figure S3: RPB-6 occupancy signatures are similar across cell types and different to controls.

(A-B) Pearson correlation heatmaps based on genome-wide normalised aligned read count maps for the seam (A) and hypodermis (B). The correlation coefficient for each pairwise comparison is printed in each cell of the heatmap. For both promoters, the *dam:rpb-6* samples show higher correlation than the control samples. **(C)** Scatterplots and Pearson correlations across replicates, cell types and developmental stages based on read-count normalised scores of GATC fragments participating in protein-coding genes. The correlation coefficient *r* values are shown in each corresponding cell. Note that these values are not identical to those reported in Fig. 2B, likely due to differences in signal distribution across GATC fragments in protein-coding genes.

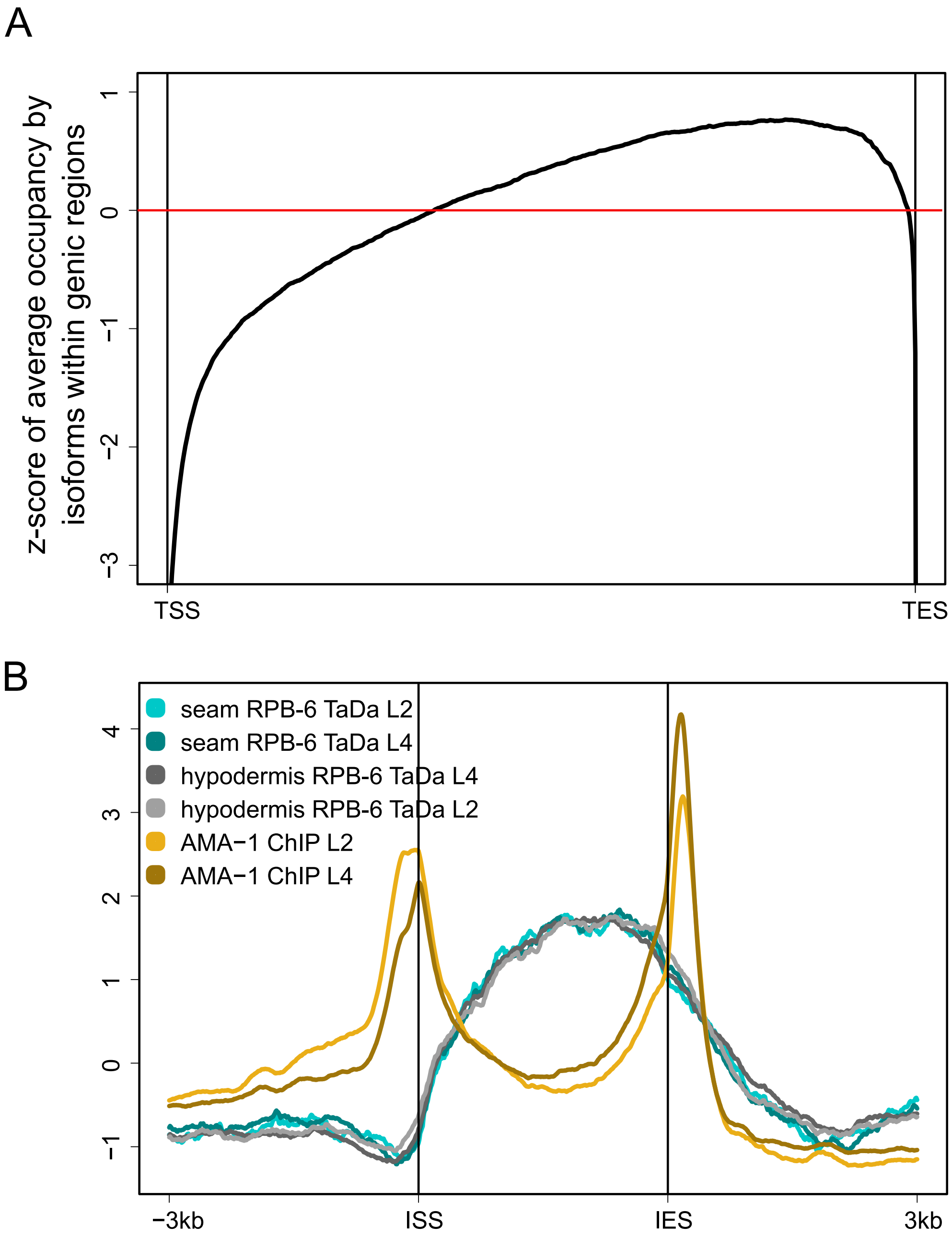
A
z-score of average occupancy by isoforms within genic regions
1
0
−1
−2
−3
TSS
TES
B
seam RPB-6 TaDa L2
seam RPB-6 TaDa L4
hypodermis RPB-6 TaDa L4
hypodermis RPB-6 TaDa L2
AMA−1 ChIP L2
AMA−1 ChIP L4
4
3
2
1
0
−1
−3kb
ISS
IES
3kb

Figure S4

Figure S4: Isoform positional enrichment within genes does not explain the RPB-6 occupancy signal preference for 3' regions.

(A) Deviation from the average occupancy of gene sequences by isoforms shows increased coverage of 3' regions compared to 5' regions of gene sequences annotated based on the largest transcript. Red line indicates the average isoform occupancy across the gene length. **(B)** Aggregation plot for the RPB-6 TaDa signal from the different cell types and developmental stages along with whole-animal AMA-1 ChIP-seq signal with the averaged signal anchored at the start and end sites (ISS and IES respectively) of all isoforms of *C. elegans* protein-coding genes, pushed into a pseudo-length of 3kb, along with a region 3kb upstream and 3kb downstream. In Y-axes show z-scores for the plotted sequence length in both cases.

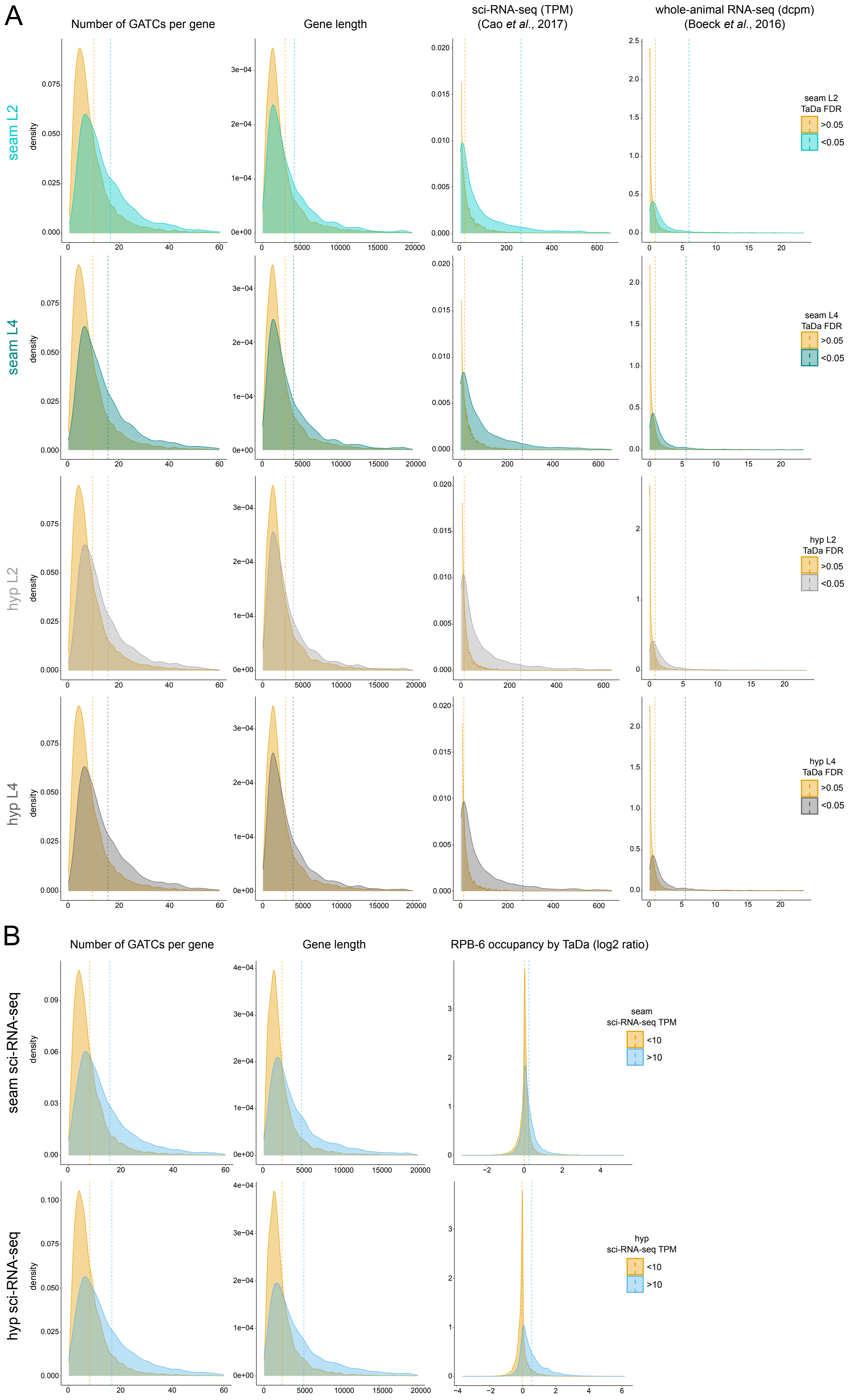
A
Number of GATCs per gene
Gene length
sci-RNA-seq (TPM) (Cao et al., 2017)
whole-animal RNA-seq (dcpm) (Boeck et al., 2016)
seam L2
seam L4
hyp L2
hyp L4
density
seam L2 TaDa FDR
>0.05
<0.05
seam L4 TaDa FDR
>0.05
<0.05
hyp L2 TaDa FDR
>0.05
<0.05
hyp L4 TaDa FDR
>0.05
<0.05
B
Number of GATCs per gene
Gene length
RPB-6 occupancy by TaDa (log2 ratio)
seam sci-RNA-seq
hyp sci-RNA-seq
seam sci-RNA-seq TPM
<10
>10
hyp sci-RNA-seq TPM
<10
>10

Figure S5

Figure S5: TaDa-identified expressed genes show higher expression in RNA-seq experiments and are biased for GATC availability.

(A) Density plots of protein coding genes, separated in groups of expressed or non-expressed based on TaDa (FDR<0.05), per cell type and developmental stage plotted against: the number of GATCs within gene, gene length, sci-RNA-seq expression values (in TPM: transcripts per million) (Cao et al., 2017) and stage-matched whole-animal RNA-seq expression values (in dcpm: depth of coverage per base per million reads) (Boeck et al., 2016). For all cell types and stages TaDa expressed genes showed significantly increased sci-RNA-seq and whole-animal RNA-seq expression values compared to non-expressed, as well as higher numbers of GATCs and consequently longer gene lengths. **(B)** Density plots of protein coding genes, separated in groups of expressed or non-expressed based on sci-RNA-seq (TPM>10) (Cao et al., 2017), per cell type plotted against: the number of GATCs within gene, gene length and TaDa RPB-6 occupancy values ($\log_2$(*rpb-6:dam/NLS-GFP:dam*)). In both cell types expressed genes showed significantly longer gene length and likely consequently more GATCs than non-expressed, as well as higher RPB-6 occupancy values. In A and B dashed lines indicate the distribution mean and statistical significance of difference in the distributions was determined with a Kolmogorov-Smirnov test, $p < 2.2\times10^{-16}$. The X-axis is limited to the 99th percentile of values and for sci-RNA-seq the Y-axis is limited to 0.02 for visualisation.

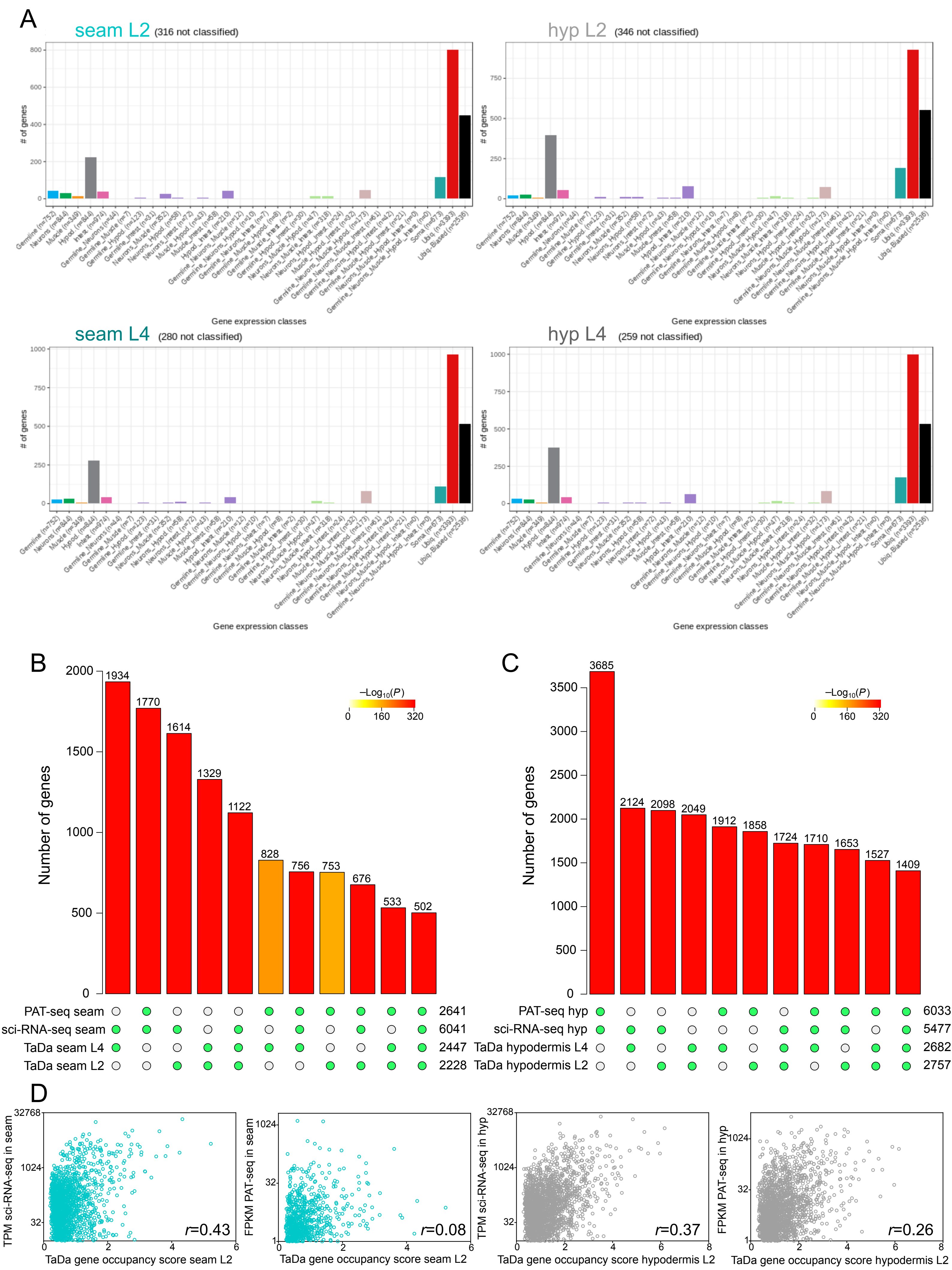
A
seam L2 (316 not classified)
hyp L2 (346 not classified)
seam L4 (280 not classified)
hyp L4 (259 not classified)
# of genes
Gene expression classes
Germline (n=752)
Neurons (n=844)
Muscle (n=349)
Hypod (n=844)
Intest (n=974)
Soma (n=673)
Ubiq (n=3393)
Ubiq-Biased (n=2536)
B
C
Number of genes
–Log10(P)
0
160
320
1934
1770
1614
1329
1122
828
756
753
676
533
502
3685
2124
2098
2049
1912
1858
1724
1710
1653
1527
1409
PAT-seq seam 2641
sci-RNA-seq seam 6041
TaDa seam L4 2447
TaDa seam L2 2228
PAT-seq hyp 6033
sci-RNA-seq hyp 5477
TaDa hypodermis L4 2682
TaDa hypodermis L2 2757
D
TPM sci-RNA-seq in seam
FPKM PAT-seq in seam
TPM sci-RNA-seq in hyp
FPKM PAT-seq in hyp
TaDa gene occupancy score seam L2
TaDa gene occupancy score hypodermis L2
r=0.43
r=0.08
r=0.37
r=0.26

Figure S6

Figure S6: TaDa-identified expressed genes overlap significantly with other published transcriptomes.

(A) Barplots of numbers of TaDa-identified expressed genes for each cell type and developmental stage that are in common with gene-sets classified based on their tissue specificity (Serizay et al., 2020). **(B-C)** Barplots of the sizes and statistical significance, assessed by a Fisher's exact test, of all possible intersections between TaDa, sci-RNA-seq genes over a 10 TPM threshold (Cao et al., 2017) and PAT-seq-identified sets of expressed genes (Blazie et al., 2017) in the seam cells (B) and the hypodermis (C). All overlaps are highly significant. **(D)** Correlation scatterplots of expression levels for L2 genes common between seam (left) or hypodermis (right) TaDa sets and the sci-RNA-seq or the PAT-seq datasets for seam cells and hypodermis. For TaDa the $\log_2$*(dam:rpb-6/dam:NLS-GFP)* scores are used as a measure of expression levels, for sci-RNA-seq the values are transcripts per million reads (TPM) and for PAT-seq fragments per kilobase of transcript per million reads (FPKM). All correlation analyses showed significant although weak correlation (indicated by the *r* values) across methods with $p<0.0001$ for all.

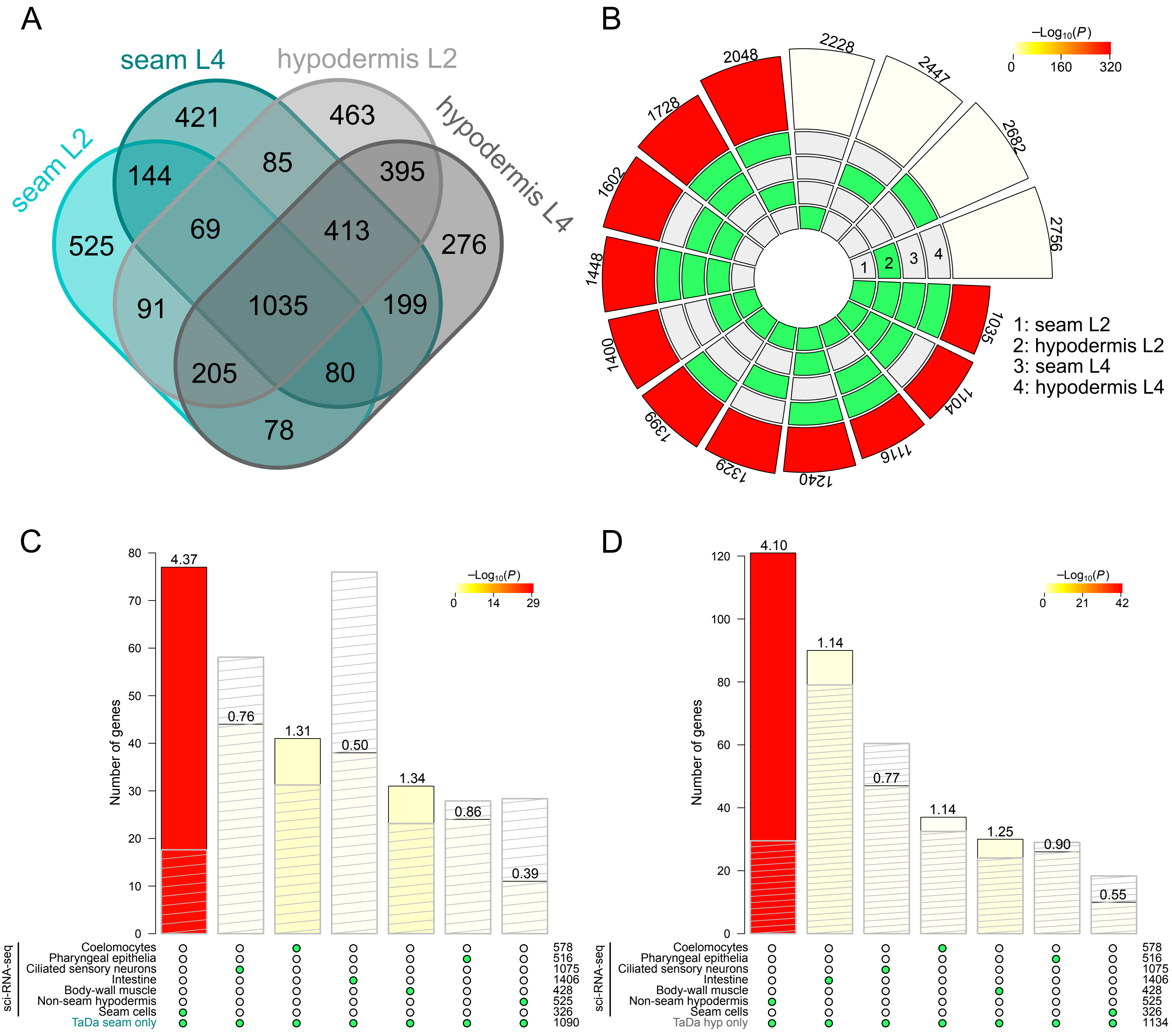
A
seam L2
seam L4
hypodermis L2
hypodermis L4
525
421
463
276
144
85
395
69
413
91
1035
199
205
80
78
B
−Log10(P)
0
160
320
2048
2228
2447
2682
2756
1728
1602
1448
1400
1399
1329
1240
1116
1104
1035
1
2
3
4
1: seam L2
2: hypodermis L2
3: seam L4
4: hypodermis L4
C
Number of genes
−Log10(P)
0
14
29
4.37
0.76
1.31
0.50
1.34
0.86
0.39
sci-RNA-seq
Coelomocytes
Pharyngeal epithelia
Ciliated sensory neurons
Intestine
Body-wall muscle
Non-seam hypodermis
Seam cells
TaDa seam only
578
516
1075
1406
428
525
326
1090
D
Number of genes
−Log10(P)
0
21
42
4.10
1.14
0.77
1.14
1.25
0.90
0.55
sci-RNA-seq
Coelomocytes
Pharyngeal epithelia
Ciliated sensory neurons
Intestine
Body-wall muscle
Non-seam hypodermis
Seam cells
TaDa hyp only
578
516
1075
1406
428
525
326
1134

Figure S7

Figure S7: Intersections of TaDa-identified expressed gene sets show significant overlap across cell types and reveal seam cell and hypodermal-specific genes.

(A-B) Multiple intersections of all the acquired gene-sets expressed in the seam and hypodermis in both stages. The Venn diagram (A) presents the number of genes that are shared across datasets or are unique to the cell type and/or developmental stage. The circular plot (B) reports the sizes of all pairwise and higher-order intersections between the sets (indicated in green whether they are included in each comparison) and indicates that they are highly significant (in red) with a Fisher's exact test. **(C-D)** Barplots representing intersections between TaDa seam-only (C) or TaDa hypodermis-only (D) gene sets and cell-type-specific genes from 7 non-related cell types from sci-RNA-seq (genes with a TPM>10 and 5-fold higher than the 2nd most expressing cell type) (Cao et al., 2017). The colour of the bar signifies statistical significance assessed by a Fisher's exact test, gridded bars indicate expected overlap based on gene set and genome size and the enrichment fold between observed and expected overlaps is printed on top of bars. The most significant intersections are those between gene sets specific for the same-cell type ($p \leq 7.06 \times 10^{-29}$).

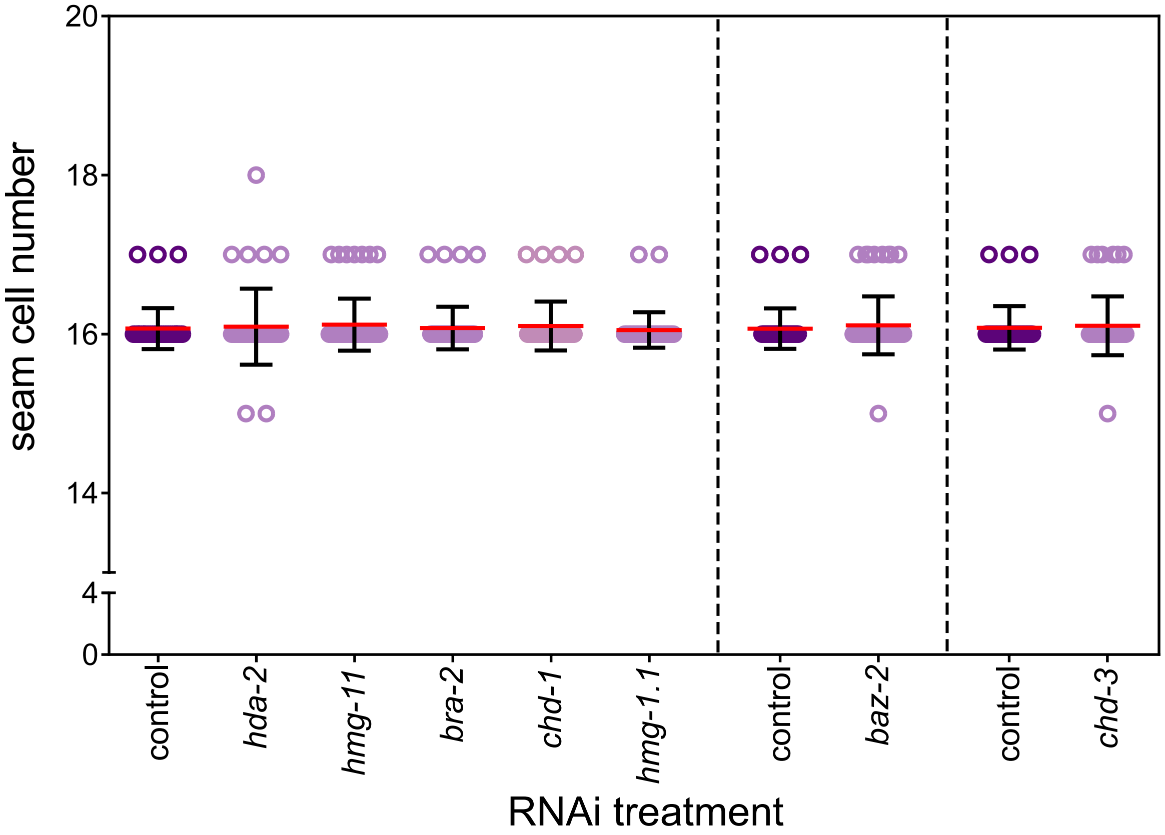

20
18
16
14
4
0
seam cell number
control
hda-2
hmg-11
bra-2
chd-1
hmg-1.1
control
baz-2
control
chd-3
RNAi treatment

Figure S8

Figure S8: RNAi-screen for putative chromatin factors regulating seam cell patterning.

Quantification of seam cell number at the late L4 stage of RNAi treated animals carrying the *SCMp::GFP* reporter in a WT background ($39 \leq n \leq 63$ animals per treatment). Targeted chromatin factors are indicated on the X-axis and sets of treatments performed on the same day are separated from others by dashed lines, having their respective control. No significant changes to the mean or variance were observed in both backgrounds for the presented treatments.

Supplemental Tables

Table S1. List of genes expressed in seam cells and hypodermis at L2 and L4 by TaDa and their overlap with sci-RNAseq and PAT-seq.

Table S2. List of genes (including transcription and chromatin factors) expressed in seam cells at L2 and L4 by TaDa.

Table S3. List of strains used in this study.

Strain	Background	Genotype
N2	N2	wild isolate *C. elegans from Bristol, UK*
JR667	N2	*unc-119(e2498::Tc1) III; wIs51[SCMp::GFP + unc-119(+)] V*
EG6699	N2	*ttTi5605 II; unc-119(ed3) III; oxEx1578.*
MBA250	N2	*icbIs2[arf-3::pes-10::GFP:CAAX::unc-54] I; icbSi2[dpy-7p::mCherry:H2B::unc-54 3'UTR+cb-unc-119] IV; wIs51[SCMp::GFP+unc-119(+)] V*
MBA467	N2	*icbSi25[pDK16(srf-3ap::GFPo-H2B::unc-54 3'UTR + cb-unc-119)] II; unc-119(ed3) III*
MBA468	N2	*icbSi26[pDK1(psrf-3ap::GFPo-H2B::unc-54 3'UTR + cb-unc-119)] II; unc-119(ed3) III*
MBA488	N2	*icbEx121[pDK18(dpy-7syn1::mCherry-H2B::unc-54 3'UTR) pBJ36, pRF4]; icbIs2[arf-3::GFP:CAAX::unc-54 3'UTR]I; wIs51[SCMp::GFP + unc-119(+)] V*
MBA489	N2	*icbEx122[pDK18(dpy-7syn1::mCherry-H2B::unc-54 3'UTR) pBJ36, pRF4]; icbIs2[arf-3::GFP:CAAX::unc-54 3'UTR]I; wIs51[SCMp::GFP + unc-119(+)] V*
MBA490	N2	*icbEx123[pDK18(dpy-7syn1::mCherry-H2B::unc-54 3'UTR) pBJ36, pRF4]; icbIs2[arf-3::GFP:CAAX::unc-54 3'UTR]I; wIs51[SCMp::GFP + unc-119(+)] V*
MBA496	N2	*icbSi32[pDK26(srf-3bp::GFPo-H2B::unc-54 3'UTR +cb-unc-119)] II; unc-119(ed3) III*
MBA497	N2	*icbSi33[pDK26(srf-3bp::GFPo-H2B::unc-54 3'UTR +cb-unc-119)] II; unc-119(ed3) III*
MBA540	N2	*icbSi42[pDK32(srf-3i1::pes-10::GFPo-H2B::unc-54 3'UTR + cb-unc-119)] II; unc-119(ed3) III*
MBA541	N2	*icbSi43[pDK32(srf-3i1::pes-10::GFPo-H2B::unc-54 3'UTR + cb-unc-119)] II; unc-119(ed3) III*
MBA542	N2	*icbSi44[pDK32(srf-3i1::pes-10::GFPo-H2B::unc-54 3'UTR + cb-unc-119)] II; unc-119(ed3) III*
MBA687	N2	*icbSi71[pDK62(cb-unc-119 + dpy-7syn1::wormCherry::Dam-myc:ama-1::unc-54 3'UTR)] II; unc-119(ed3) III*
MBA688	N2	*icbSi72[pDK65(dpy-7syn1::wormCherry::Dam-myc:rpb-6::unc-54 3'UTR + cb-unc-119)] II; unc-119(ed3) III*
MBA692	N2	*icbSi76[pDK54(srf-3i1::pes-10::wormCherry::Dam-myc:NLS-GFP::unc-54 3'UTR + cb-unc-119)] II; unc-119(ed3) III*
MBA693	N2	*icbSi77[pDK64(dpy-7syn1::wormCherry::Dam-myc:NLS-GFP::unc-54 3'UTR + cb-unc-119)] II; unc-119(ed3) III*
MBA694	N2	*icbSi78[pDK46(cb-unc-119 + srf-3i1::pes-10::wormCherry::Dam-myc:ama-1::unc-54 3'UTR)] II; unc-119(ed3) III*
MBA698	N2	*icbSi82[pDK55(srf-3i1::pes-10::wormCherry::Dam-myc:rpb-6::unc-54 3'UTR + cb-unc-119)]; unc-119(ed3) III*
MBA1138	N2	*icbEx261[pDK133(srf-3i1::Δpes-10::mir-42-44::p10 3'UTR), myo-2::dsRed, pBJ36]; unc-119(e2498::Tc1) III; wIs51[SCMp::GFP + unc-119(+)] V*
MBA1139	N2	*icbEx262[pDK133(srf-3i1::Δpes-10::mir-42-44::p10 3'UTR), myo-2::dsRed, pBJ36]; unc-119(e2498::Tc1) III; wIs51[SCMp::GFP + unc-119(+)] V*
MBA1140	N2	*icbEx263[pDK133(srf-3i1::Δpes-10:::mir-42-44::p10 3'UTR), myo-2::dsRed, pBJ36]; unc-119(e2498::Tc1) III; wIs51[SCMp::GFP + unc-119(+)] V*
MBA1141	N2	*icbEx267[pDK147(dpy-7syn1::mir-42-44::p10 3UTR), myo-2::dsRed, pBJ36]; unc-119(e2498::Tc1) III; wIs51[SCMp::GFP + unc-119(+)] V*
MBA1142	N2	*icbEx265[pDK139(srf-3i1::Δpes-10:::mir-47::p10 3'UTR), myo-2::dsRed, pBJ36]; unc-119(e2498::Tc1) III; wIs51[SCMp::GFP + unc-119(+)] V*
MBA1143	N2	*icbEx266[pDK139(srf-3i1::Δpes-10:::mir-47::p10 3'UTR), myo-2::dsRed, pBJ36]; unc-119(e2498::Tc1) III; wIs51[SCMp::GFP + unc-119(+)] V*
MBA1144	N2	*icbEx264[pDK139(srf-3i1::Δpes-10:::mir-47::p10 3'UTR), myo-2::dsRed, pBJ36]; unc-119(e2498::Tc1) III; wIs51[SCMp::GFP + unc-119(+)] V*
MBA1145	N2	*icbEx268[pDK148(dpy-7syn1::mir-47::p10 3UTR), myo-2::dsRed, pBJ36]; unc-119(e2498::Tc1) III; wIs51[SCMp::GFP + unc-119(+)] V*
MBA1146	N2	*icbEx269[pDK148(dpy-7syn1::mir-47::p10 3UTR), myo-2::dsRed, pBJ36]; unc-119(e2498::Tc1) III; wIs51[SCMp::GFP + unc-119(+)] V*
MBA1192	N2	*icbEx292[pDK158(srf-3i1-mut::Δpes-10::outron::>hda-1 fragment>::srf-3a intron5::<hda-1 fragment<::p10 3UTR), myo-2::dsRed, pBJ36]]; wIs51[SCMp::GFP + unc-119(+)] V*

Table S4. Primers used in this study (general oligos and smFISH probes).

Oligo name	Sequence 5' -> 3'
DK27	CTCATCTCTGAAGAGGATCTGCGGCATGGAATGGCCGATGAGGACGATTATC
DK28	CGAGATGGCTAGCACTTAATTAACTGCATGCTACCAATCAGCGAGTTGAA
DK33	CTTACTTGCACTTATAATACGACTCACTAGGCTAGCTTTTCACTTTGGTTTTCACG
DK34	TTCTCCTTTACTCATCCTAGGATTCCAGATGGTACCTGAAA
DK35	GGTACCATCTGGAATCCTAGGATGAGTAAAGGAGAAGAATT
DK36	CCGATCCCCCGGGCACTTAAGCTTGTAGAGCTCGTCCATTC
DK37	GACGAGCTCTACAAGCTTAAGTGCCCGGGGGATCGGTGGAG
DK38	CACGGGCGCGAGATGTTAATTAATTACTTGCTGGAAGTGTACT
DK39	ACTTCCAGCAAGTAATTAATTAACATCTCGCGCCCGTGCCTCT
DK40	GGCCCGGGCTACGTAATACGACTCACTTAAAAACAGTTATGTTTGGTATA
DK41	AGGCCTTGACTAGAGGGTACCAGAGCTCACCAACTTTGTATAGAAAAGTT
DK42	CCACTTCAAAAAAGCGCGATTTTTCTTCATCCTAGGGTTACTACTTATAC
DK43	CTCATCTCTGAAGAGGATCTGCGGCATGCTATGACTGCTCCAAAGAAGAA
DK44	GCGAGATGGCTAGCACTTAATTAATGCATGCTATTTGTATAGTTCATCCA
DK46	ACATTTTGTTCTTGATAAGTTTAAACATTTATGGTCTCAAAGGGTGAAGA
DK47	GTAATTGGACTTAGAAGTCAGAGGCAATTTTTACTTGCTGGAAGTGTACT
DK48	GGGGACAAGTTTGTACAAAAAAGCAGGCTATGGTCAGCAAGGGAGAGGCA
DK49	CTTGTAGAGCTCGTCCATTCCT
DK50	AGGAATGGACGAGCTCTACAAGTGCCCGGGGGATCGGTGGAGC
DK59	AATTCTTCTCCTTTACTCATCCTAGGATTTCCTTTTGATTGTGAAGATTC
DK64	TTGCACTTATAATACGACTCACTAGGCTAGGTGAGTTCTTAACTTAACCT
DK65	CGGGGATCCTCTAGAGTCGACCAAGGCCTGATTTCCTTTTGATTGTGAAG
DK66	GGCCGTGAATCTTCACAATCAAAAGGAAATCAGGCCTTGGTCGACTCTAG
DK67	TGAACAATTCTTCTCCTTTACTCATCCTAGCTCCAAGCAAGGGTCCTCCT
DK89	GGGGACAACTTTGTATAGAAAAGTTGGTGAGTTCTTAACTTAACCT
DK90	GGGGACTGCTTTTTTGTACAAACTTGCTCGAGCTCCAAGCAAGGGTCCTCCT
DK102	CTCATTGACAATCAACCTGCCAATTGTTTCTCGGATGACCTAGGATGACGTCATTCCTGTGCTC
DK103	GTAAGTCTTAACAAAAGTTCAATTGGCTCGATCAAGTAAAATTT
DK107	GAACAATTCTTCTCCTTTACTCATCCTAGGCTGAAAGTTAAAAATTACAG
DK113	GGGGACAACTTTGTATAGAAAAGTTGCGGGAATTCCTTTGATAACG
DK114	GGGGACTGCTTTTTTGTACAAACTTGTTATCAAGAACAAAATGTAA
DK217	TTATACTGTAATTTTTAACTTTCAGCCTAGTTCGCGGACCTTTGTGGGTG
DK218	ATTTGTTATTTTAAAAACGATTCATTTAATATAAACCTTACAGGAACGGAAATAC
DK219	TATTCTTACATTTTGTTCTTGATAACCTAGTTCGCGGACCTTTGTGGGTG
DK220	TTATACTGTAATTTTTAACTTTCAGCCTAGGAGAGCCGACTGAAACTGAA
DK221	ATTTGTTATTTTAAAAACGATTCATTTAATGATTACCGCACACCTTTGCC
DK222	TATTCTTACATTTTGTTCTTGATAACCTAGGAGAGCCGACTGAAACTGAA
DK247	TCGAAGACTAAGGTATGACTCGTTTTCACAGCGAC
DK248	TCGAAGACTAAAGCTCGGTGATTCCATCGCGAAGT
MB100	ATCGGGAGGCGAACCTAACTG
MB106	AGGCAGAATGTGAACAAGACTCG
MB270	CGGGAATTCCTTTGATAACGATAATTGGGTTATTGTGTC
MB271	CGGGAATTCTCATTTATCACACCCTGTTCTT

efl-3 probe (1:50)		*srf-3* probe (1:50)	
agagccgaagatagttgtca	cgggattttctttgttccaa	atcaaaattgccgtcttcat	atccgacgaatggagattca
ccgggtggaatattttcttt	ggttcaggaccacagtaaac	actattctgtagtgttaacc	catgctacgacgacggctac
tctggatctgttgttcgatt	aaacatcgaagcttccggtt	tcacgagctcgagaatatct	tccagcaaatccagaaagac
ctggagatggtcttggaaca	gtgaactactcagaaggcgt	gtactaacaaacatggcgtc	tcctttgagaatcttttcga
gaagcatcggagagttgtga	caattttgtcagtctgtgct	cttcagttaaccaaacagct	tccaaagagatactggtgct
gtcacttctagatcgtcatc	ttttgttctgagagcgttct	ctcttcttgagctactaaga	actgccatttgaacatttct
cccaaactcttttcttttct	tgcgatttttggagtattcc	gcgtggatgaatcttctagg	atcgccgaaaatgatgctgg
ctgtctcttcattgatagca	tggtgatgatctgacagctg	ctctaggatttgagttcgta	cagttttcgaatcctgcata
attcattttccttgcaacgg	ccacctctgcgaacataaac	ctttcaatgtgtcatagggt	ccatacaacaatccatactc
cgtagattcgtcgtttttca	tatctcattttctcagctgc	tgtagatcatggcgggaatg	gtaccaaagtacagtaagcc
ttgttagttttctgcatggc	attgatgctactgatcccaa	ctgccacgtagaaaagatta	caacagagagacctccaaca
aagagactctaatccttgcc	gatgtcattggatgctgtga	gttgcggcgtctaaatgtga	tcggcgtacttgatgcaaac
ctctggaagaccttcttcaa	ggaagttgaagaggagccat	aagctgtgatgttatcatga	ggcaaaattcttggcaatgt
cacgtggtgatgacaactca	ggctttggaatctctggaag	gtgaaaatagccgccgtgaa	agagaatgatggcgactgaa
gagccgacaatatccttgaa	tgtaaactgcttttgagcct	agagatctgcgcaagatgat	aggaaaatggatccgattgt
ggtcggtgttgaagtacatg	attgaatcgaggcctagacg	tgcgaaccattgtgttcgat	aaatgacaagtgaggcgccg
acgagtgtcaactctcgatc	ttgcacaggtgtatagtcag	ctccgacaaataatacagct	caccatcgactgatgagatg
tcaaaaaccgtctgcacagc	agatacgagtgggcgtattg	ccttgaagttgaacgagaga	acggaatctcgccacgtaat
cttttcggatattcttcggg	gttgcgaagattcttgtgga	agatgattcctttgcttttg	agacaaaacgcctcttttgt
aactgtgctagctacatcga	aatgttttggcttgacgtct		
aaaaccttctgtttccggat	cttcgattcgccaagaatgt		
tataagcgtctgcatcgact	tgtgctcgaacgtattctga		
aaccaggacattggcaatgt	cacctggaatgcggaagatg		
acctttttaatgagcccaag	ctccgaagactttttttggt		

Table S5. List of transgenes and injection mixes used in this study.

Extrachromosomal array number	Genetic contents	Composition of injection mix
icbEx121	*[pDK18(dpy-7syn1::mCherry-H2B::unc-54 3'UTR) pBJ36, pRF4]*	pDK18 10 ng/µl, pRF4 35 ng/µl, pBJ36 80 ng/µl
icbEx122	*[pDK18(dpy-7syn1::mCherry-H2B::unc-54 3'UTR) pBJ36, pRF4]*	pDK18 10 ng/µl, pRF4 35 ng/µl, pBJ36 80 ng/µl
icbEx123	*[pDK18(dpy-7syn1::mCherry-H2B::unc-54 3'UTR) pBJ36, pRF4]*	pDK18 10 ng/µl, pRF4 35 ng/µl, pBJ36 80 ng/µl
icbEx261	*[pDK133(srf-3i1::Δpes-10::mir-42-44::p10 3'UTR), myo-2::dsRed, pBJ36]*	pDK133 50 ng/µl, myo-2::dsRed 5 ng/µl, pBJ36 70 ng/µl
icbEx262	*[pDK133(srf-3i1::Δpes-10::mir-42-44::p10 3'UTR), myo-2::dsRed, pBJ36]*	pDK133 50 ng/µl, myo-2::dsRed 5 ng/µl, pBJ36 70 ng/µl
icbEx263	*[pDK133(srf-3i1::Δpes-10:::mir-42-44::p10 3'UTR), myo-2::dsRed, pBJ36]*	pDK133 50 ng/µl, myo-2::dsRed 5 ng/µl, pBJ36 70 ng/µl
icbEx264	*[pDK139(srf-3i1::Δpes-10:::mir-47::p10 3'UTR), myo-2::dsRed, pBJ36]*	pDK139 50 ng/µl, myo-2::dsRed 5 ng/µl, pBJ36 70 ng/µl
icbEx265	*[pDK139(srf-3i1::Δpes-10:::mir-47::p10 3'UTR), myo-2::dsRed, pBJ36]*	pDK139 50 ng/µl, myo-2::dsRed 5 ng/µl, pBJ36 70 ng/µl
icbEx266	*[pDK139(srf-3i1::Δpes-10:::mir-47::p10 3'UTR), myo-2::dsRed, pBJ36]*	pDK139 50 ng/µl, myo-2::dsRed 5 ng/µl, pBJ36 70 ng/µl
icbEx267	*[pDK147(dpy-7syn1::mir-42-44::p10 3UTR), myo-2::dsRed, pBJ36]*	pDK147 50 ng/µl, myo-2::dsRed 5 ng/µl, pBJ36 70 ng/µl
icbEx268	*[pDK148(dpy-7syn1::mir-47::p10 3UTR), myo-2::dsRed, pBJ36]*	pDK148 50 ng/µl, myo-2::dsRed 5 ng/µl, pBJ36 70 ng/µl
icbEx269	*[pDK148(dpy-7syn1::mir-47::p10 3UTR), myo-2::dsRed, pBJ36]*	pDK148 50 ng/µl, myo-2::dsRed 5 ng/µl, pBJ36 70 ng/µl
icbEx292	*[pDK158(srf-3i1-mut:delta-pes-10::outron::>hda-1 fragment>::srf-3a intron5::<hda-1 fragment<::p10 3UTR),myo-2::dsRed, pBJ36]*	pDK158 50 ng/ul, myo-2::dsRed 5 ng/ul, pBJ36 70 ng/ul

Single-copy insertion number	Genetic contents	Composition of injection mix
icbSi25	*[pDK16(srf-3ap::GFPo-H2B::unc-54 3'UTR + cb-unc-119)]*	pDK16 50 ng/µl, pCFJ601 50 ng/µl, pMA122 10 ng/µl, pGH8 10 ng/µl, myo-2;;dsRed 2,5 ng/µl, pCFJ104 5 ng/µl
icbSi26	*[pDK16(srf-3ap::GFPo-H2B::unc-54 3'UTR + cb-unc-119l*	pDK16 50 ng/µl, pCFJ601 50 ng/µl, pMA122 10 ng/µl, pGH8 10 ng/µl, myo-2;;dsRed 2,5 ng/µl, pCFJ104 5 ng/µl
icbSi32	*[pDK26(srf-3bp::GFPo-H2B::unc-54 3'UTR +cb-unc-119)]*	pDK26 50 ng/µl, pCFJ601 50 ng/µl, pMA122 10 ng/µl, pGH8 10 ng/µl, myo-2;;dsRed 2,5 ng/µl, pCFJ104 5 ng/µl
icbSi33	*[pDK26(srf-3bp::GFPo-H2B::unc-54 3'UTR +cb-unc-119)]*	pDK26 50 ng/µl, pCFJ601 50 ng/µl, pMA122 10 ng/µl, pGH8 10 ng/µl, myo-2;;dsRed 2,5 ng/µl, pCFJ104 5 ng/µl
icbSi42	*[pDK32(srf-3i1::pes-10::GFPo-H2B::unc-54 3'UTR + cb-unc-119)]*	pDK32 50 ng/µl, pCFJ601 50 ng/µl, pMA122 10 ng/µl, pGH8 10 ng/µl, myo-2;;dsRed 2,5 ng/µl, pCFJ104 5 ng/µl
icbSi43	*[pDK32(srf-3i1::pes-10::GFPo-H2B::unc-54 3'UTR + cb-unc-119)]*	pDK32 50 ng/µl, pCFJ601 50 ng/µl, pMA122 10 ng/µl, pGH8 10 ng/µl, myo-2;;dsRed 2,5 ng/µl, pCFJ104 5 ng/µl
icbSi44	*[pDK32(srf-3i1::pes-10::GFPo-H2B::unc-54 3'UTR + cb-unc-119)]*	pDK32 50 ng/µl, pCFJ601 50 ng/µl, pMA122 10 ng/µl, pGH8 10 ng/µl, myo-2;;dsRed 2,5 ng/µl, pCFJ104 5 ng/µl
icbSi71	*[pDK62(cb-unc-119 + dpy-7syn1::wormCherry::Dam-myc:ama-1::unc-54 3'UTR)]*	pDK62 50 ng/µl, pCFJ601 50 ng/µl, pMA122 10 ng/µl, pGH8 10 ng/µl, myo-2;;dsRed 2,5 ng/µl, pCFJ104 5 ng/µl
icbSi72	*[pDK65(dpy-7syn1::wormCherry::Dam-myc:rpb-6::unc-54 3'UTR + cb-unc-119)]*	pDK65 50 ng/µl, pCFJ601 50 ng/µl, pMA122 10 ng/µl, pGH8 10 ng/µl, myo-2;;dsRed 2,5 ng/µl, pCFJ104 5 ng/µl
icbSi76	*[pDK54(srf-3i1::pes-10::wormCherry::Dam-myc:NLS-GFP::unc-54 3'UTR + cb-unc-119)]*	pDK54 50 ng/µl, pCFJ601 50 ng/µl, pMA122 10 ng/µl, pGH8 10 ng/µl, myo-2;;dsRed 2,5 ng/µl, pCFJ104 5 ng/µl
icbSi77	*[pDK64(dpy-7syn1::wormCherry::Dam-myc:NLS-GFP::unc-54 3'UTR + cb-unc-119)]*	pDK64 50 ng/µl, pCFJ601 50 ng/µl, pMA122 10 ng/µl, pGH8 10 ng/µl, myo-2;;dsRed 2,5 ng/µl, pCFJ104 5 ng/µl
icbSi78	*[pDK46(cb-unc-119 + srf-3i1::pes-10::wormCherry::Dam-myc:ama-1::unc-54 3'UTR)]*	pDK46 50 ng/µl, pCFJ601 50 ng/µl, pMA122 10 ng/µl, pGH8 10 ng/µl, myo-2;;dsRed 2,5 ng/µl, pCFJ104 5 ng/µl
icbSi82	*[pDK55(srf-3i1::pes-10::wormCherry::Dam-myc:rpb-6::unc-54 3'UTR + cb-unc-119)]*	pDK55 50 ng/µl, pCFJ601 50 ng/µl, pMA122 10 ng/µl, pGH8 10 ng/µl, myo-2;;dsRed 2,5 ng/µl, pCFJ104 5 ng/µl

Table S6. List of RNAi clones used in this study.

WormBase Gene ID	Public Name	Sequence Name	Ahringer RNAi library Geneservice_location
WBGene00009899	*efl-3*	*F49E12.6*	II-6K03
WBGene00000275	*bub-1*	*R06C7.8*	I-3H11
WBGene00001834	*hda-1*	*C53A5.3*	V-9F11
WBGene00009672	*F43G9.12*	*F43G9.12*	I-4C12
WBGene00001974	*hmg-4*	*T20B12.8*	III-3P10
WBGene00010369	*chd-1*	*H06O01.2*	I-3M20
WBGene00003664	*nhr-74*	*C27C7.3*	I-5P17
WBGene00001976	*hmg-11*	*T05A7.4*	II-3N12
WBGene00006554	*tbx-35*	*ZK177.10*	II-4O24
WBGene00001835	*hda-2*	*C08B11.2*	II-5N08
WBGene00001971	*hmg-1.1*	*Y48B6A.14*	II-9G13
WBGene00017757	*bra-2*	*F23H11.1*	III-1E22
WBGene00001470	*baz-2*	*ZK783.4*	III-4E11
WBGene00006970	*zag-1*	*F28F9.1*	IV-1P04
WBGene00003606	*nhr-7*	*F54D1.4*	IV-6C03
WBGene00020062	*nhr-270*	*R13D11.8*	V-1B09
WBGene00003649	*nhr-59*	*T27B7.1*	V-2M07
WBGene00001210	*egl-46*	*K11G9.4*	V-4L02
WBGene00003717	*nhr-127*	*T13F3.3*	V-10J08
WBGene00000482	*chd-3*	*T14G8.1*	X-6G01
WBGene00007433	*swsn-7*	*C08B11.3*	II-5N10
